# DNA glycosylases provide antiviral defence in prokaryotes

**DOI:** 10.1038/s41586-024-07329-9

**Published:** 2024-04-17

**Authors:** Amer A. Hossain, Ying Z. Pigli, Christian F. Baca, Søren Heissel, Alexis Thomas, Vincent K. Libis, Ján Burian, Joshua S. Chappie, Sean F. Brady, Phoebe A. Rice, Luciano A. Marraffini

**Affiliations:** 1 Laboratory of Bacteriology, The Rockefeller University, New York, NY USA; 2 Department of Biochemistry and Molecular Biology, University of Chicago, Chicago, IL USA; 3 Proteomics Resource Center, The Rockefeller University, New York, NY USA; 4 Laboratory of Genetically Encoded Small Molecules, The Rockefeller University, New York, NY USA; 5 Department of Molecular Medicine, Cornell University, Ithaca, NY USA; 6 Howard Hughes Medical Institute, The Rockefeller University, New York, NY USA

**Keywords:** Bacteriophages, Bacterial evolution

## Abstract

Bacteria have adapted to phage predation by evolving a vast assortment of defence systems^1^. Although anti-phage immunity genes can be identified using bioinformatic tools, the discovery of novel systems is restricted to the available prokaryotic sequence data^2^. Here, to overcome this limitation, we infected *Escherichia coli* carrying a soil metagenomic DNA library^3^ with the lytic coliphage T4 to isolate clones carrying protective genes. Following this approach, we identified Brig1, a DNA glycosylase that excises α-glucosyl-hydroxymethylcytosine nucleobases from the bacteriophage T4 genome to generate abasic sites and inhibit viral replication. Brig1 homologues that provide immunity against T-even phages are present in multiple phage defence loci across distinct clades of bacteria. Our study highlights the benefits of screening unsequenced DNA and reveals prokaryotic DNA glycosylases as important players in the bacteria–phage arms race.

## Abstract (web summary)

A screen utilizing an environmental DNA library in *Escherichia coli* is used to identify Brig1, a previously unknown anti-phage defence system with homologues across distinct clades of bacteria.

## Main

Bacteria have evolved a diverse battery of immune systems to counteract infections by viruses and plasmids^1^. Multiple studies have used bioinformatic analyses to identify new antiviral genes and immune systems present within defence islands^4–6^ as well as in pathogenicity islands^7^ and prophage elements^8^. More recently, one study performed a functional selection for antiviral immune systems in the *E. coli* pangenome, identifying immune systems harboured by *E. coli* strains that had gone unnoticed in previous bioinformatic searches^9^. Although these studies have undoubtedly expanded our understanding of the diversity of immune systems present in bacteria, they have all relied on the availability of sequenced genomes. Analyses of 16S ribosomal RNA sequences suggest that uncultivated and unsequenced microorganisms represent the majority of bacterial lineages^2^. This ‘microbial dark matter’, which is beginning to be accessed through single-cell genomics^10^, arguably contains a vast assortment of unknown genetic pathways, including those involved in anti-phage defence. To tap into this uncharted sequence space, we screened a library of environmental DNA (eDNA) constructed in *E. coli* for clones showing resistance or immunity to phage T4 infection. This library was constructed by cloning microbial DNA isolated from an arid soil collected in Arizona into a cosmid vector^3,11^. After challenging this library with the lytic coliphage T4 we isolated Brig1, a DNA glycosylase from an unknown organism that provides immunity through the excision of α-glucosyl-hydroxymethylcytosine (α-glucosyl-hmC) nucleobases present in the T4 genome^12^, thus inhibiting phage replication after infection. Our study illustrates both a powerful method for the discovery of new anti-phage defence systems as well as a unique mechanism of anti-phage immunity.

## Isolation of defence genes from eDNA

**Fig. 1:**
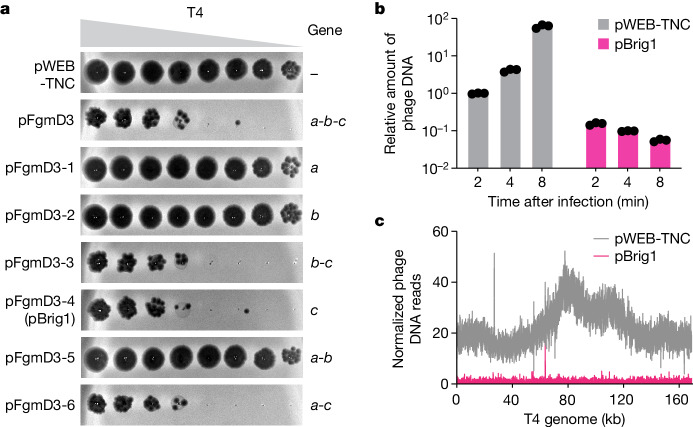
Screening of an eDNA library uncovers an unknown gene that protects *E. coli* against phage T4 infection by preventing viral replication. **a**, Tenfold serial dilutions of phage T4 on lawns of *E. coli* EC100 that harbour the pWEB-TNC cosmid, which carries different genes present in a three-gene operon isolated after the screening of an eDNA library with this phage. Gene *c* encodes Brig1. Data are representative of three independent experiments. **b**, Quantitative PCR analysis of T4 DNA by amplification of the *gp43* gene. Viral DNA was extracted from infected *E. coli* EC100 cells carrying pWEB-TNC or pBrig1 at 2, 4 and 8 min after the addition of phage at a multiplicity of infection (MOI) of 1. Fold-change values were calculated relative to the pWEB-TNC 2 min time point. Data are mean ± s.e.m. for three independent experiments. **c**, Normalized next-generation sequencing reads of T4 DNA, mapped to the viral genome. DNA for sequencing was obtained 8 min after infection of *E. coli* EC100 carrying pWEB-TNC or pBrig1 at an MOI of 5. Source Data

**Extended Data Fig. 1:**
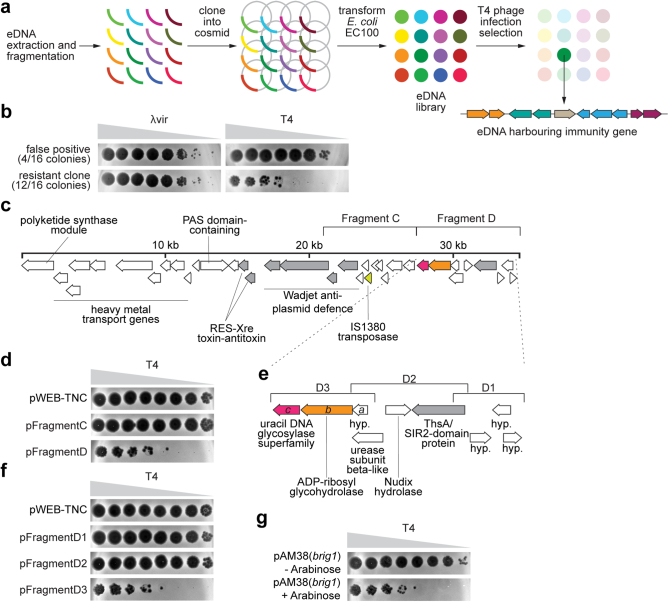
Selection of soil metagenomic DNA fragments that provide immunity against phage T4 in *E. coli.* **a**, Schematic showing the experimental setup for the screening of soil DNA (environmental DNA, eDNA) libraries for the presence of clones resistant to T4 infection. eDNA is extracted from soil samples and cleaved into large fragments of ~40 kb that are cloned into *E. coli* EC100. The library is infected with phage T4 in top agar plates to isolate surviving colonies. The cosmid from the T4-resistant colonies is extracted and further analyzed to identify and confirm immunity genes. **b**, Tenfold serial dilutions of λvir or T4 phage on lawns of *E. coli* EC100 cells generated from one of 16 colonies sampled randomly from the eDNA library population that survived T4 infection. Top row is a representative result for a surviving colony that did not contain a bona fide immunity gene in the eDNA (4/16 false positives). Bottom row is a representative result for a surviving colony that harboured a cosmid carrying immunity genes (12/16 true positives). **c**, Genes present within the 34.5 kb soil metagenomic DNA fragment that provided T4 immunity. Subcloned regions (Fragments C and D) are indicated. Transposases and defence genes are shown in yellow and grey, respectively. **d**, Tenfold serial dilutions of phage T4 on lawns of *E. coli* EC100 carrying pWEB-TNC or cosmids containing Fragments C or D. Data are representative of two independent experiments. **e**, Genes present within Fragment D, showing the subclones generated for further analysis, Fragments D1-3; hyp, hypothetical protein. **f**, Tenfold serial dilutions of phage T4 on lawns of *E. coli* EC100 carrying pWEB-TNC or cosmids containing Fragments D1-3. Data are representative of two independent experiments. **g**, Tenfold serial dilutions of phage T4 on lawns of bacteria expressing Brig1 using an arabinose-inducible promoter, in the presence (+Arabinose) or absence (−Arabinose) of the inducer. Data are representative of three independent experiments.

To uncover novel anti-phage defence systems present in unsequenced bacterial genomes, we screened an eDNA library generated in an earlier study. The library consists of large (approximately 40 kb) DNA fragments, extracted from a soil sample collected in Arizona, that were cloned into pWEB-TNC cosmids, packaged into λ phage and transfected into *E. coli* EC100 cells^3,11^. The library, which contains at least 10 million clones, was infected with phage T4 to select resistant clones that enable colony formation (Extended Data Fig. 1a). We picked 16 random colonies and used a plaque assay to determine that twelve carried immunity to T4 phage infection, but did not provide immunity to the unrelated phage λvir (Extended Data Fig. 1b; see also Supplementary Fig. 1 for unedited images of plaque assays and gel electrophoresis). Sequencing of the selected cosmids revealed a 34.5-kb DNA insert (Extended Data Fig. 1c), that probably belongs to the phylum Actinobacteria (Supplementary Data File 1). Further subcloning of cosmid fragments (Extended Data Fig. 1c–f and Supplementary Fig. 2, which shows replicates for these and all subsequent plaque assays reported in this study) determined that gene *c*, which encodes an unknown protein belonging to the superfamily of uracil DNA glycosylases, was solely responsible for immunity (Fig. 1a and Extended Data Fig. 1g). This gene lies within the vicinity of other putative defence systems (Extended Data Fig. 1c,e), including a Thoeris ThsA-like gene^13^ and Wadjet^14^, a genetic context that suggests that gene *c* is part of a bacterial defence island present in the isolated eDNA.

**Extended Data Fig. 2:**
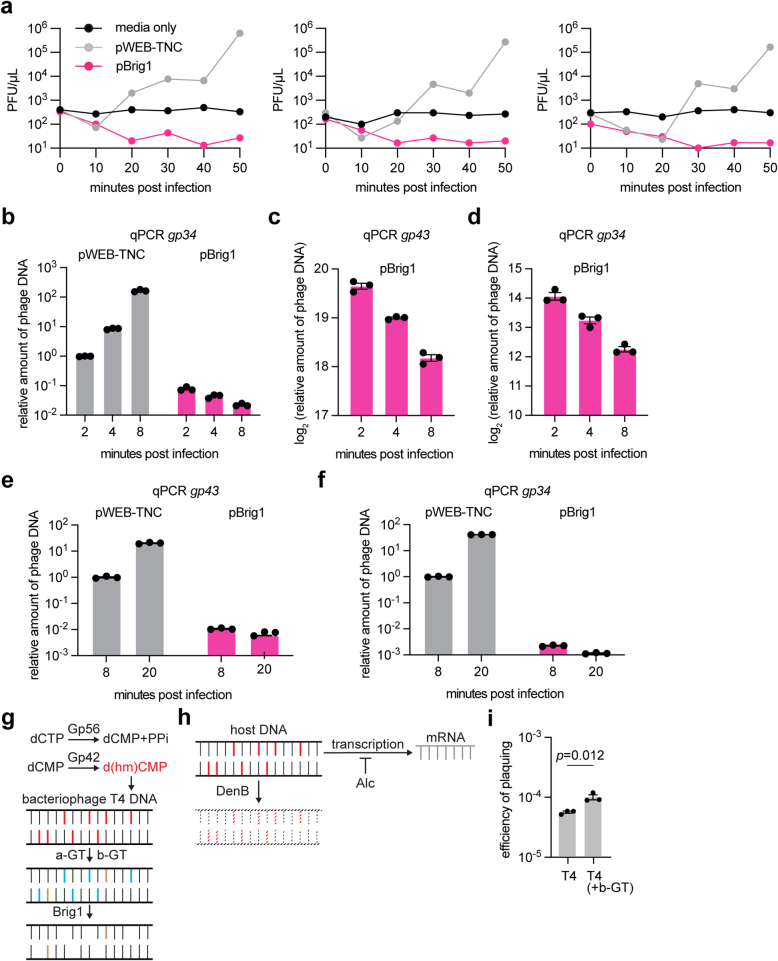
Brig1 inhibits phage T4 DNA replication. **a**, Enumeration of T4 plaque-forming units (PFU) in supernatants of infected cultures at different times after phage addition. Cultures of *E. coli* EC100 carrying pWEB-TNC or pBrig1 were infected at MOI 0.01. Addition of T4 phage to media lacking bacteria was used as a control. **b**, Quantitative PCR analysis of T4 DNA through amplification of the *gp34* gene. Viral DNA was extracted from infected *E. coli* EC100 cells carrying pWEB-TNC or pBrig1 at 2, 4 and 8 min after the addition of phage at an MOI of 1. Fold-change values were calculated relative to the pWEB-TNC 2-minute time point. Mean values are reported for three independent experiments. Error bars report the standard error of the mean (s.e.m.). **c**, Quantitative PCR results from Fig. 1b for DNA extracted from infected *E. coli* EC100 cells carrying pBrig1; plotted using a log_2_ scale. Mean values are reported for three independent experiments. Error bars report the standard error of the mean (s.e.m.). **d**, Same as **c** but for results shown in **b**. **e**, Same as **b** but using DNA extracted at 8- and 20-minutes post infection, amplifying the *gp43* gene. Fold-change values were calculated relative to the pWEB-TNC 8-minute time point. **f**, Same as **b** but using DNA extracted at 8- and 20-minutes post infection. Fold-change values were calculated relative to the pWEB-TNC 8-minute post-infection time point. For all quantitative PCR graphs, mean values are reported for three independent experiments, with error bars reporting the standard error of the mean (s.e.m.). **g**, Schematic of the cytosine modification pathway in phage T4, including a model for Brig1 immunity through excision of α-glucosyl-hydroxymethylcytosine nucleobases from the viral genome to generate abasic sites. Gp42, dCMP hydroxymethylase; Gp56, dCTPase; α-GT, α-glucosyltransferase; β-GT, β-glucosyltransferase. **h**, Model showing the activity of T4-encoded enzymes that affect DNA containing cytosine bases. Alc, dC-specific premature transcriptional terminator; DenB, dC-specific ssDNA endonuclease. **i**, Efficiency of plaquing of T4 and T4 phage passaged through *E. coli* EC100/p(*b-gt*), [T4( + β-GT)], on lawns of *E. coli* EC100 carrying pBrig1. Mean are reported for three independent experiments. Error bars report the standard error of the mean (s.e.m.; *p*-value is reported for an unpaired two-sided Student’s *t*-test. Source Data

To investigate how gene *c* affects T4 infection, we first determined that it does not affect phage adsorption (Extended Data Fig. 2a). We also performed quantitative PCR (qPCR) to measure phage DNA accumulation (at two different loci, *gp43* and *gp34*) in infected cells at 2, 4, 8 and 20 min post-infection (Fig. 1b and Extended Data Fig. 2b–f). We found that, in contrast to susceptible hosts, which showed a steady increase in phage DNA over time, the T4 DNA content decreased in *E. coli* expressing gene *c* (Fig. 1b and Extended Data Fig. 2b–f). This result demonstrates that gene *c* not only inhibits T4 DNA replication but also causes a slight and gradual depletion of the phage DNA within the infected population (Extended Data Fig. 2c,d). Finally, we performed next-generation sequencing of *E. coli* cells infected with T4 for 8 min, which showed that phage DNA reads were severely depleted across the entire T4 genome in cells expressing gene *c* (Fig. 1c). We named this gene bacteriophage replication inhibition DNA glycosylase 1 (*brig1*) (see sequences in Supplementary Information). The cosmid harbouring only gene *c*, pFgmD3-4 (Fig. 1a), was therefore renamed pBrig1.

## Brig1 targets glucosylated DNA bases

**Fig. 2:**
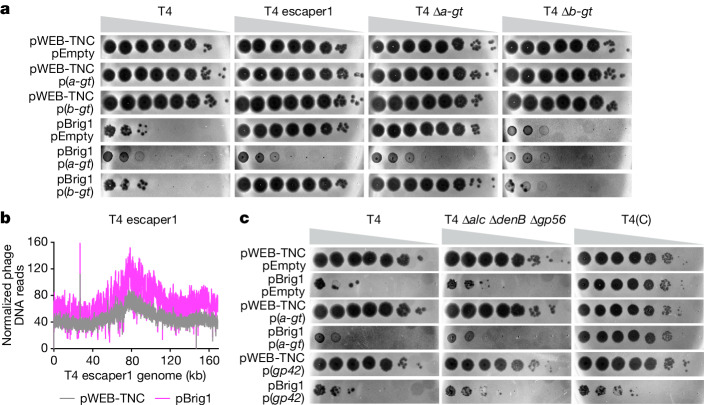
The antiviral DNA glycosylase Brig1 targets α-glucosyl-hmC nucleobases to restrict T4 viral replication. **a**, Tenfold serial dilutions of different T4 phage stocks on lawns of *E. coli* EC100, each lawn carrying cosmid pWEB-TNC or pBrig1, and plasmids pEmpty, p(*a-gt*) or p(*b-gt*). Plaque images of one representative experiment from three independent experiments are shown. **b**, Normalized next-generation sequencing reads of T4 escaper1 DNA, mapped to the viral genome. DNA for sequencing was obtained 8 min after infection of *E. coli* EC100 carrying pWEB-TNC or pBrig1 at an MOI of 5. **c**, Tenfold serial dilutions of different T4 phage stocks on lawns of *E. coli* EC100, each lawn carrying cosmid pWEB-TNC or pBrig1, and plasmid pEmpty, p(*a-gt*) or p(*gp42*). Plaque images of one representative experiment from three independent experiments are shown. Source Data

To understand how *brig1* affects T4 replication, we isolated an ‘escaper’ phage that completely bypassed Brig1 defence (Fig. 2a) and displayed a very similar pattern of DNA reads during infection in the presence or absence of *brig1* expression in *E. coli* hosts (Fig. 2b). Sequencing of the viral DNA revealed a single-base-pair deletion within the T4 α-glucosyltransferase (*a-gt*) gene, resulting in a frameshift (escaper1; Supplementary Table 1). Sequencing of PCR products obtained using DNA isolated from another 18 escaper phages showed additional mutations in *a-gt*, most of them frameshifts (Supplementary Table 1). We also generated an in-frame deletion of this gene, phage T4 Δ*a-gt*, which phenocopied the escaper1 mutation (Fig. 2a). Finally, plasmid-borne expression of *a-gt* rescued *E. coli* from lysis by both T4 escaper1 and T4 Δ*a-gt* (Fig. 2a), a result that demonstrates that this gene is required for Brig1 defence.

α-Glucosyltransferase (α-GT) and β-glucosyltransferase (β-GT) add glucose in α- and β-linkage to around 70% and 30% of the 5-hydroxymethylcytosine (hmC) bases in the T4 genome^12,15^, respectively (Extended Data Fig. 2g–h). Brig1 provided strong immunity upon infection with a mutant phage carrying an in-frame deletion of *b-gt* (T4 Δ*b-gt*) (Fig. 2a). In addition, we constructed a cytosine-containing T4 mutant phage, T4(C), with the genotype Δ*alc* Δ*denB* Δ*gp56* Δ*gp42* (Extended Data Fig. 2h), which has been shown to lack hmC in its genome^16^. This phage was resistant to Brig1 targeting, in contrast to the triple mutant Δ*alc* Δ*denB* Δ*gp56* phage that harbours both *gp42* to synthesize hmC nucleobases and *a-gt* to glycosylate the bases (Fig. 2c). Moreover, overexpression of *gp42*, but not *a-gt* alone, sensitized T4(C) to Brig1 targeting (Fig. 2c). Finally, we passaged T4 on a strain that overexpressed β-GT to investigate the effect of an increase in the fraction of β-glucosylated hmC nucleobases on Brig1 immunity. We found that the resulting phage, T4(+β-GT), displayed a small but significant increase in propagation in the presence of the enzyme (Extended Data Fig. 2i). Together, these data demonstrate that Brig1 targets α-glucosylated hmC nucleobases in the viral DNA to provide defence against T4.

## Brig1 excises α-glucosyl-hmC nucleobases

**Fig. 3:**
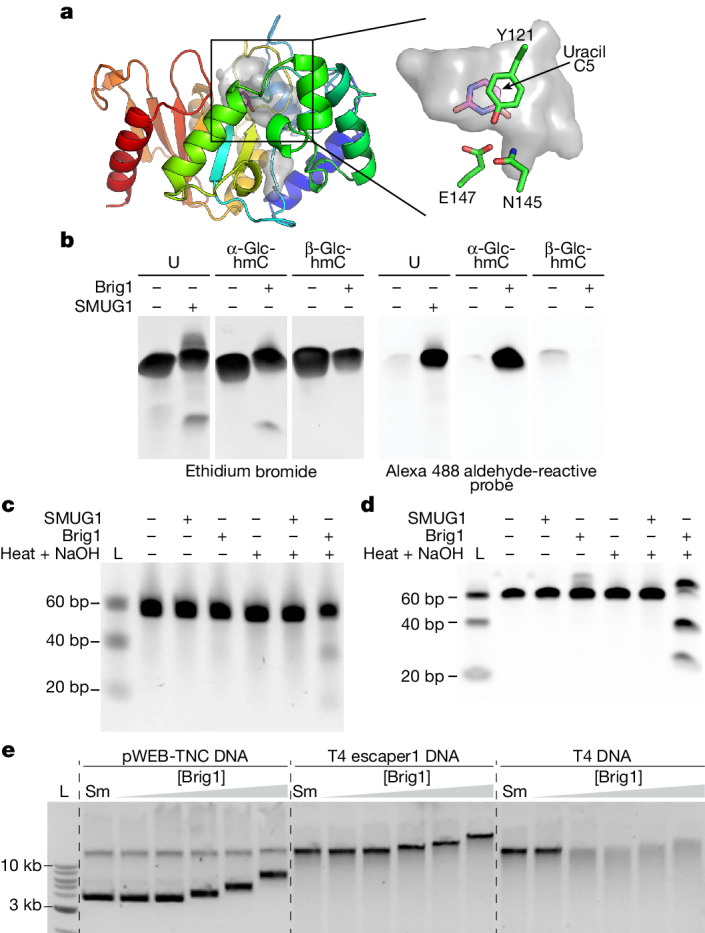
Brig1 excises α-glucosyl-hmC nucleobases. **a**, AlphaFold2 structure of Brig1, coloured by position (N terminus (blue) to C terminus (red)), with cavities shown in translucent grey. Right, zoomed-in view of putative glycosylase pocket, with uracil (pink–purple sticks) from Protein Data Bank (PDB) 4ZBY modelled into it, and showing the amino acid residues that could participate in substrate binding. **b**, Polyacrylamide gel electrophoresis of 60-nt single-stranded oligonucleotides containing a single modified base, incubated with either SMUG1 or Brig1 at 37 °C overnight, and then treated with an aldehyde-reactive Alexa 488 fluorescent probe to label and detect abasic sites. The same gel was stained with ethidium bromide to detect ssDNA. U, uracil; α-Glc-hmC, α-glucosyl-hmC; β-Glc-hmC, β-glucosyl-hmC. Data are representative of one experiment. **c**, Urea-PAGE of dsDNA substrates harbouring α-glucosyl-hmC in the top strand (see Extended Data Fig. 5a) treated either with SMUG1 or Brig1 at 37 °C overnight, with and without heating in the presence of sodium hydroxide for 30 min. Gels were stained with ethidium bromide. L, ssDNA size ladder. Data are representative of two independent experiments. **d**, Native PAGE of dsDNA substrates harbouring α-glucosyl-hmC in both strands (Extended Data Fig. 5a) treated either with SMUG1 or Brig1 at 37 °C overnight, with and without heating in the presence of sodium hydroxide for 30 min. Gels were stained with ethidium bromide. L, dsDNA size ladder. Data are representative of one experiment. **e**, Agarose gel electrophoresis of T4, T4 escaper1 or pWEB-TNC DNA (500 ng) treated with increasing concentrations (2, 20, 200, 400 or 800 nM) of Brig1 or with 10 units of NEB SMUG1 (Sm) for 30 min at 37 °C. Data are representative of three independent experiments.

**Extended Data Fig. 3:**
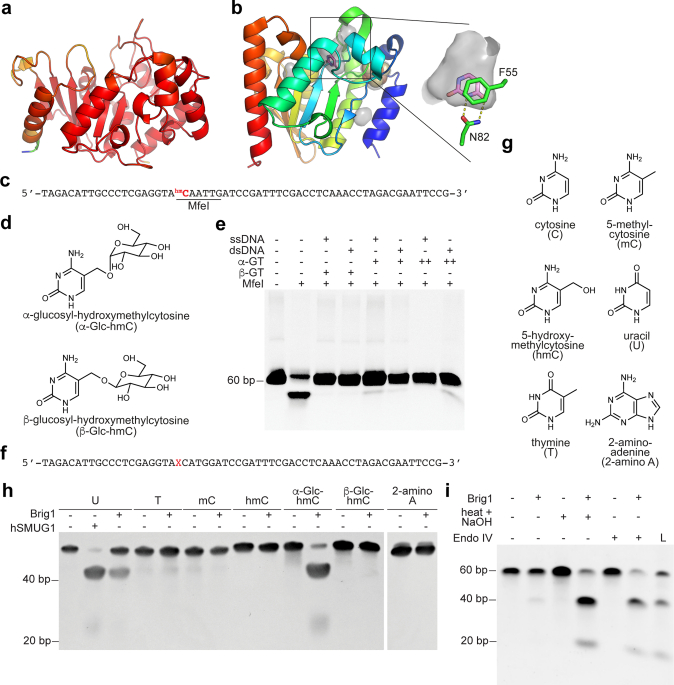
Oligonucleotides and nucleobases used in Brig1 DNA glycosylase assays. **a**, AlphaFold2 structure of Brig1, coloured by pLDDT (a per-residue model confidence score). Red to blue spectrum represents high to low confidence of secondary structure prediction. **b**, Crystal structure of a family 4 uracil DNA glycosylase from *Sulfolobus tokodaii* (PDB 4ZBY, a close structural homologue of Brig1) showing the uracil substrate (pink-purple sticks) in the glycosylase pocket and the Fe-S cluster (yellow and orange spheres). The structure is coloured by position (N-terminal, blue; to C-terminal, red), with cavities shown in translucent grey. Inset, zoomed in view of the glycosylase pocket, showing the uracil substrate and the amino acid residues that participate in uracil binding. **c**, Sequence of the 60-nt single-stranded oligonucleotide used for testing the DNA glycosylase activity of Brig1. The nucleobase in red was synthesized as 5-hydroxymethylcytosine (hmC) and subsequently α- or β-glucosylated. The MfeI restriction site is underlined. MfeI digestion was used to confirm glucosylation after annealing a complementary bottom strand oligonucleotide to create a dsDNA substrate for the MfeI enzyme. **d**, Chemical structures of α- and β-glucosyl-hydroxymethylcytosine nucleobases. **e**, MfeI digestion of dsDNA substrates generated by annealing the oligonucleotide shown in panel **c** containing a 5-hydroxymethylcytosine nucleobase with a complementary bottom strand oligonucleotide. In each case, prior to MfeI digestion of the dsDNA oligonucleotides, the ssDNA (“ssDNA”) or dsDNA (“dsDNA”) was either untreated or treated with a low (+) or high (++) concentration of T4 α- or T4 β-glucosyltransferase (α-GT or β-GT, respectively). Data are representative of one experiment. **f**, Sequence of the 60-nt oligonucleotide used for testing the DNA glycosylase activity of Brig1 or hSMUG1 in Fig. 3b and panel **h** below. The red X was replaced by the nucleobases shown in **g**. **g**, Chemical structures of different nucleobases used to test the specificity of Brig1. **h**, Polyacrylamide gel electrophoresis of 60-nt single-stranded oligonucleotides containing a single modified base, incubated with either hSMUG1 or Brig1 at 37 °C overnight, and then treated with NaOH and heat for 30 min prior to gel electrophoresis. Gels were stained with ethidium bromide to detect ssDNA. U, uracil; T, thymine, mC, 5-methylcytosine; hmC, 5-hydroxymethylcytosine; 2-aminoA, 2-aminoadenine. Data are representative of two independent experiments. **i**, PAGE of the reaction products that resulted from the sequential treatment of the oligonucleotide shown in panel **c**, containing an α-glucosyl-hydroxymethylcytosine nucleobase, with Brig1 at 37 °C overnight followed by 50 units of NEB Endonuclease IV at 37 °C for 4 h or NaOH for 30 min at 90 °C. Gels were stained with ethidium bromide. L, ssDNA size ladder. Data are representative of two independent experiments.

We performed an AlphaFold2 protein structure prediction^17,18^ of Brig1, which generated a high-confidence structural model (Fig. 3a and Extended Data Fig. 3a) that we used to find structural homologues. The top hits were all uracil DNA glycosylases, with the best match being a uracil DNA glycosylase from the archaeon *Sulfolobus tokodaii*^19^ (Dali *Z* score^20^ 9.2) (Extended Data Fig. 3b). Uracil DNA glycosylases recognize uracil bases in DNA (which may result from polymerase error or from cytosine deamination) and initiate base excision repair by hydrolysing the N-glycosidic bond between the base and the deoxyribose sugar^21^. We therefore hypothesized that Brig1 removes α-glucosyl-hmC, but not β-glucosyl-hmC, from the T4 genome. To test this prediction, we purified Brig1 and determined its activity on a 60-nt single-stranded DNA (ssDNA) oligonucleotide substrate containing a single hmC residue within an MfeI restriction site (Extended Data Fig. 3c), to which we introduced α-glucosyl-hmC and β-glucosyl-hmC modifications (Extended Data Fig. 3d) using purified T4 α-GT and β-GT enzymes. Glucosylation was confirmed by annealing a complementary oligonucleotide to generate a double-stranded DNA (dsDNA) substrate for MfeI digestion, a restriction endonuclease that can cleave hmC-containing, but not glucosyl-hmC-containing, target sequences^22^ (Extended Data Fig. 3e). The modified ssDNA oligonucleotides were incubated with Brig1 to determine its DNA glycosylase activity using an aldehyde-reactive fluorescent probe that can detect abasic sites. The primary product of Brig1 was a full-length oligonucleotide containing an abasic site, generated by excision of the α- but not the β-glucosyl-hmC nucleobase (Fig. 3b). As a positive control, we treated an equivalent uracil-containing oligonucleotide (Extended Data Fig. 3f,g) with SMUG1, a previously characterized human uracil DNA glycosylase^23^.

We further tested Brig1 activity by treating reaction products with heat and sodium hydroxide, conditions that accelerate the cleavage of the DNA backbone at abasic sites via β-elimination^24^ and thus enable the detection of these sites as DNA fragments. Using this method, Brig1 exhibited robust base excision activity on the α-glucosyl-hmC-containing substrate but not on ssDNA substrates harbouring β-glucosyl-hmC, hmC, 5-methylcytosine or 2-aminoadenine (Extended Data Fig. 3g,h). Finally, we confirmed Brig1 activity using a third method to detect abasic sites. We incubated the oligonucleotide product with Endonuclease IV, an apurinic/apyrimidinic endonuclease that cleaves the sugar–phosphate backbone adjacent to an abasic site^24,25^. This treatment resulted in the cleavage of the ssDNA substrate at the position where the glucosyl-hmC nucleobase is located (Extended Data Fig. 3i).

**Extended Data Fig. 4:**
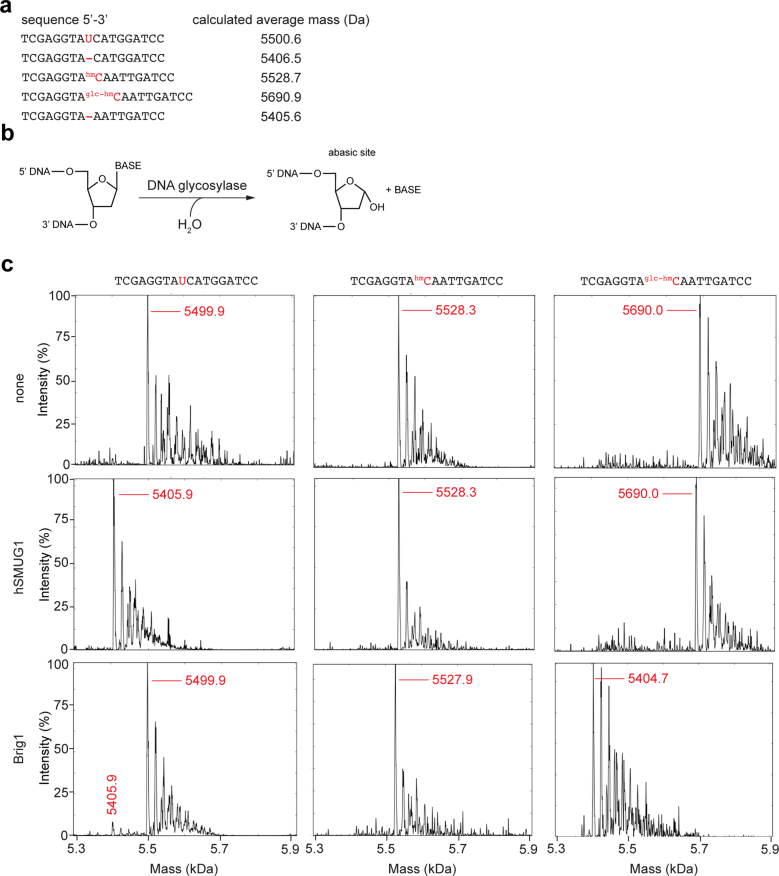
Mass spectrometry of a Brig1-generated abasic site. **a**, Theoretical average masses, in daltons (Da), of the different oligonucleotides used for mass spectrometry. “-” indicates an abasic site; ^hm^C, 5-hydroxymethylcytosine; ^glc-hm^C, α-glucosyl-hydroxymethylcytosine. **c**, Deconvoluted zero-charge mass spectra from high resolution mass spectrometry of the oligonucleotides shown on the top of each column incubated without any enzyme, with hSMUG1 or with Brig1 at 37 °C overnight. Major peaks are indicated in red. Source Data

To obtain direct evidence of the removal of the glucosyl-hmC nucleobase, we performed high-resolution mass spectrometry. We treated an 18-nt ssDNA oligonucleotide containing either a single uracil, hmC or α-glucosyl-hmC nucleobase (Extended Data Fig. 4a) with SMUG1 or Brig1. With SMUG1 and Brig1 treatment of the uracil- and α-glucosyl-hmC-containing oligonucleotides, respectively, we recorded, in each case, strong primary peaks with mass values equivalent to the loss of the excised target nucleobase and the gain of a water molecule that were interpreted as the introduction of an abasic site (Extended Data Figs. 4b,c). Together, these results demonstrate that Brig1 is a DNA glycosylase that excises α-glucosyl-hmC nucleobases from ssDNA to generate abasic sites, with a high level of stereoisomeric specificity.

## Brig1 generates abasic sites in dsDNA

**Extended Data Fig. 5:**
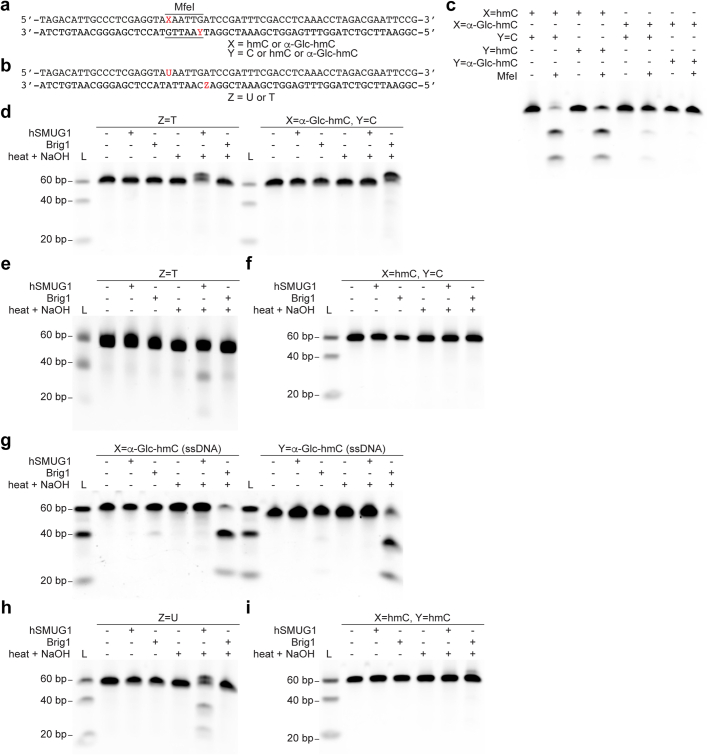
Brig1 activity on dsDNA oligonucleotides. **a**, Sequence of the dsDNA oligonucleotide used for testing the DNA glycosylase activity of Brig1. The base marked as “X” in red was synthesized as 5-hydroxymethylcytosine (hmC) and subsequently α-glucosylated (α-Glc-hmC); “Y” was synthesized as cytosine (C) or as hmC that was subsequently α-glucosylated (α-Glc-hmC). The MfeI restriction site is underlined. MfeI digestion was used to confirm glucosylation. **b**, Sequence of the dsDNA oligonucleotide used for testing the uracil DNA glycosylase activity of hSMUG1 and Brig1. The base marked as “Z” in red was synthesized as uracil (U) or thymine (T). **c**, PAGE of the reaction products obtained after MfeI digestion (40 units) of dsDNA oligonucleotides (500 ng) shown in panel **a** (the nucleotide modifications at X and Y positions are indicated). Data are representative for one experiment. **d**, PAGE of the reaction products obtained after treatment of dsDNA oligonucleotides shown in panels **a** (X in top strand is α-glucosyl-hmC; Y in bottom strand is cytosine) and **b** (Z in bottom strand is thymine), with hSMUG1 or Brig1 at 37 °C overnight, with or without additional heating with NaOH for 30 min. L, dsDNA size ladder. Data are representative of two independent experiments. **e**, Urea-PAGE of the reaction products obtained after treatment of a dsDNA oligonucleotide shown in panel **b** (Z in bottom strand is thymine), with hSMUG1 or Brig1 at 37 °C overnight, with or without additional heating with NaOH for 30 min. L, ssDNA size ladder. Data are representative of two independent experiments. **f**, PAGE of the reaction products obtained after treatment of a dsDNA oligonucleotide shown in panel **a** (X in top strand is hmC; Y in bottom strand is cytosine), with hSMUG1 or Brig1 at 37 °C overnight, with or without additional heating with NaOH for 30 min. L, dsDNA size ladder. Data are representative of one experiment. **g**, PAGE of the reaction products obtained after treatment of ssDNA oligonucleotides shown in panel **a** (X and Y are α-glucosyl-hmC), with hSMUG1 or Brig1 at 37 °C overnight, with or without additional heating with NaOH for 30 min. L, ssDNA size ladder. Data are representative of one experiment. **h**, PAGE of the reaction products obtained after treatment of a dsDNA oligonucleotide shown in panel **b** (Z in bottom strand is uracil), with hSMUG1 or Brig1 at 37 °C overnight, with or without additional heating with NaOH for 30 min. L, dsDNA size ladder. Data are representative of one experiment. **i**, same as panel **f**, but X and Y are hmC. Data are representative of one experiment.

Although the T4 genome can have ssDNA intermediates during replication^26^, most of the viral DNA is in a double-stranded form. We therefore tested whether Brig1 can also introduce abasic sites in dsDNA oligonucleotide substrates (Extended Data Fig. 5a,b) which were glucosylated at hmC sites and subsequently determined to be resistant to MfeI cleavage, which confirmed the presence of the modification (Extended Data Fig. 5c). We first treated a dsDNA substrate harbouring either a single α-glucosyl-hmC or uracil in the top strand with Brig1 or SMUG1. When separated by non-denaturing polyacrylamide gel electrophoresis (PAGE), only the products treated with heat and sodium hydroxide showed a high molecular weight species (Extended Data Fig. 5d). This is most probably owing to the generation of nicked dsDNA that runs slower on a non-denaturing gel^27^. To corroborate this, we separated the products by urea-PAGE, which revealed cleavage products generated by Brig1 and subsequent heat and sodium hydroxide treatment, but not by treatment with the glycosylase alone (Fig. 3c and Extended Data Fig. 5e). By contrast, dsDNA substrates containing hmC in the top strand that were treated with Brig1 and heat and sodium hydroxide did not show cleavage products, that are indicative of the introduction of abasic sites (Extended Data Fig. 5f). Next, after confirming that both top and bottom ssDNA oligonucleotides were subject to glycosylase activity (Extended Data Fig. 5g), we tested DNA duplex molecules containing modified bases in both strands. Brig1 treatment led to the cleavage of both strands after β-elimination, generating a dsDNA break and cleavage products that can be separated by non-denaturing, native PAGE (Fig. 3d). Similar results were obtained for SMUG1 experiments (Extended Data Fig. 5h). Finally, Brig1 did not display any activity on the dsDNA substrate with hmC in both strands (Extended Data Fig. 5i). Together, these results indicate that Brig1 is a monofunctional DNA glycosylase that generates abasic sites, with insignificant lyase activity.

## Brig1 degrades T4 phage DNA

**Extended Data Fig. 6:**
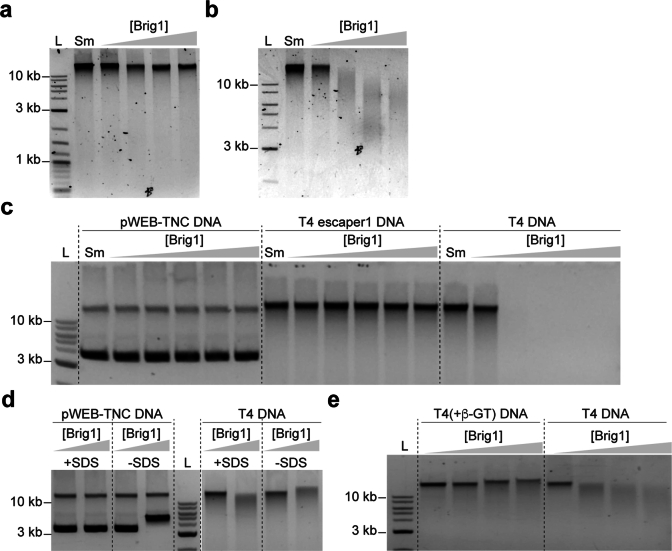
Generation of abasic sites in T4 genomic DNA by Brig1. **a**, Agarose gel electrophoresis of 125 ng of phage T4 genomic DNA treated with 10 units of hSMUG1 (“Sm”) or increasing concentrations of Brig1 (2, 20, 200, 400 nM) for 30 min at 37 °C. Electrophoresis was performed under 40 V for 3 h at 4 °C. L, DNA size ladder. Data are representative for one experiment. **b**, Same gel shown in panel **a** run under higher voltage (85, 150 and 200 V for additional 25, 8 and 8 min, respectively) at room temperature. Data are representative for one experiment. **c**, Agarose gel electrophoresis of T4, T4 escaper1 or pWEB-TNC DNA (500 ng) treated with increasing concentrations (2, 20, 200, 400, 800 nM) of Brig1 or with 10 units of hSMUG1 (“Sm”) for 30 min at 37 °C and followed by heat treatment (20 min at 65 °C) prior to electrophoresis. L, DNA size ladder. Data are representative of three independent experiments. **d**, Agarose gel electrophoresis of T4 or pWEB-TNC DNA (500 ng) treated with increasing concentrations (20, 200 nM) of Brig1 with or without treatment with SDS prior to electrophoresis. L, DNA size ladder. Data are representative of one experiment. **e**, Agarose gel electrophoresis of DNA from T4 or from T4 phage passaged through *E. coli* EC100/p(*b-gt*), which overexpresses T4 β-glucosyltransferase to increase the frequency of β-glucosyl-hydroxymethylcytosine modifications within the T4 genome [T4( + β-GT)], after treatment with increasing concentrations (2, 20, 200, 400 nM) of Brig1 for 30 min at 37 °C. L, DNA size ladder. Data are representative of one experiment.

We also tested the effect of Brig1 on T4 phage DNA in vitro. We incubated wild-type and escaper1 viral DNA with Brig1 for 30 min at 37 °C and visualized the products via agarose gel electrophoresis. Although the T4 DNA treated with Brig1 was not distinguishable from an untreated control DNA when using low voltage and low temperature (4 °C) to separate the reaction products, experiments at higher voltage and temperature resulted in DNA cleavage and degradation via β-elimination at abasic sites caused by the heat generated during electrophoresis (Fig. 3e and Extended Data Fig. 6a,b). In these conditions, increasing concentrations of Brig1 caused a mobility shift, but no degradation, in escaper1 DNA and a cosmid control DNA (pWEB-TNC) (Fig. 3e). Heating to 65 °C, or treatment of the reaction products with SDS, before electrophoresis eliminated the mobility shifts (Extended Data Fig. 6c,d), results that suggest that Brig1 can bind to non-target DNA. Finally, Brig1 treatment of phage T4(+β-GT) DNA, which contains a higher proportion of β-glucosyl-hmC nucleobases than wild-type T4 DNA (Extended Data Fig. 2i), resulted in the generation of a lower number of abasic sites, evidenced by the reduced DNA degradation via heat-promoted β-elimination during electrophoresis (Extended Data Fig. 6e). Together, these data indicate that heat-promoted β-elimination at abasic sites generated by Brig1 results in the degradation of wild-type, α-glucosylated T4 phage DNA.

## Brig1 residues important for activity

**Extended Data Fig. 7:**
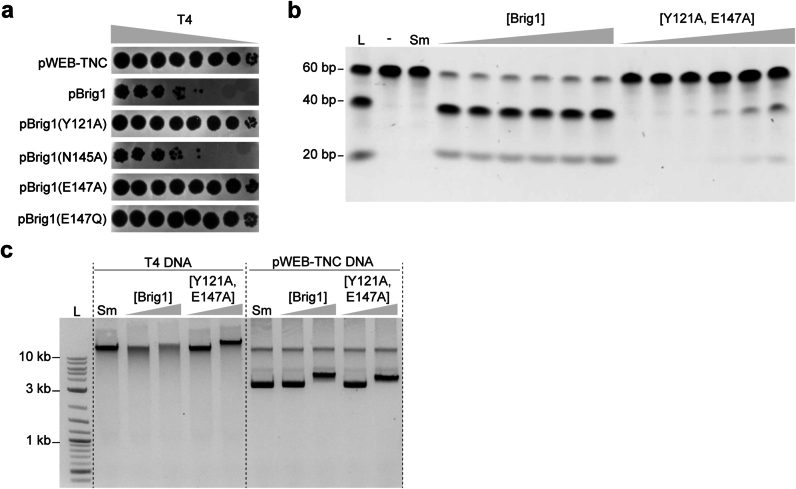
Brig1 amino acid residues involved in base excision. **a**, Tenfold serial dilutions of phage T4 on lawns of *E. coli* EC100 carrying pWEB-TNC, pBrig1 or pBrig1 harbouring substitutions in the amino acids thought to participate in catalysis shown in Fig. 3a. Plaque images of one representative experiment from three independent experiments are shown. **b**, PAGE of the reaction products obtained after treatment of the ssDNA oligonucleotide shown in Extended Data Fig. 3c, harbouring α-glucosyl-hmC, with hSMUG1 (“Sm”; 5 units), Brig1 or the Brig1 Y121A, E147A mutant (50, 100, 200, 400, 800 and 1600 nM) at 37 °C for 30 min, followed by heating with NaOH for 30 min. L, ssDNA size ladder. Data are representative of one experiment. **c**, Agarose gel electrophoresis of T4 or pWEB-TNC DNA (500 ng) treated with increasing concentrations (50, 500 nM) of Brig1, the Y121A, E147A mutant or with 10 units of hSMUG1 (“Sm”) for 30 min at 37 °C. L, DNA size ladder. Data are representative of one experiment.

The nucleotide binding pocket of Brig1 is predicted to be much larger (Fig. 3a) than that of the *S. tokodaii* uracil DNA glycosylase (Extended Data Fig. 3b), with extra space adjacent to the C5 position of the pyrimidine where the additional α-glucosyl-hydroxymethyl group would protrude (Fig. 3a and Extended Data Fig. 3d). To test whether this putative binding pocket is important for Brig1 activity, we mutated amino acids predicted to outline this area: Y121, E147 and N145 (Fig. 3a). On the basis of the structure of other related glycosylases, Y121 would stack against the flipped-out base (as is the case for F55 in *S. tokodaii* uracil DNA glycosylase; Extended Data Fig. 3b), whereas E147 would form hydrogen bonds to its Watson:Crick face. Because this residue is often asparagine rather than glutamate^28^ (for example N82 in *S. tokodaii* uracil DNA glycosylase; Extended Data Fig. 3b), we also considered the N145 residue (Fig. 3a). In vivo, the Y121A, E147A and E147Q, but not the N145A, substitutions affected Brig1-mediated immunity (Extended Data Fig. 7a). In vitro, the Y121A/E147A double mutation abrogated base excision activity on ssDNA oligonucleotides as well as on T4 DNA (Extended Data Fig. 7b,c). These results demonstrate that the putative DNA glycosylase catalytic pocket of Brig1 is important for base excision activity as well as defence against phage T4.

## Involvement of host DNA repair pathways

**Extended Data Fig. 8:**
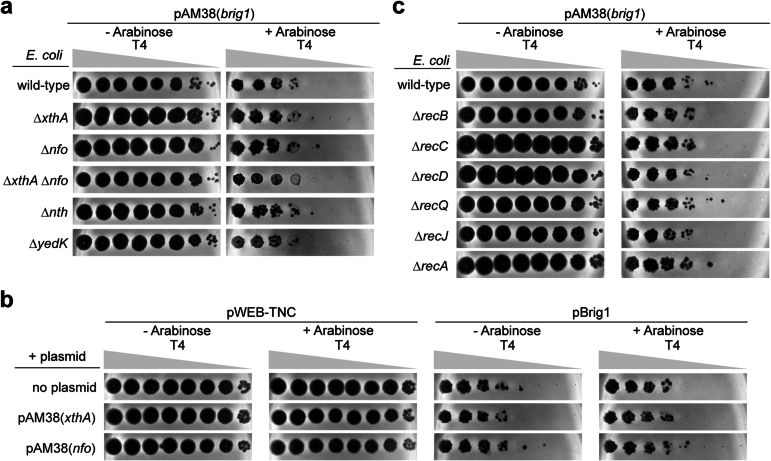
*E. coli* AP endonucleases and DNA repair proteins are not specifically required for Brig1 immunity. **a**, Tenfold serial dilutions of phage T4 on lawns of different *E. coli* BW25113 mutants with deletions of genes involved in base excision repair, carrying the pAM38 vector to express Brig1 using an arabinose-inducible promoter, in the presence (+Arabinose) or absence (−Arabinose) of the inducer. **b**, Tenfold serial dilutions of phage T4 on lawns of *E. coli* EC100, each lawn carrying cosmid pWEB-TNC or pBrig1, and plasmids pAM38(*xthA*) or pAM38(*nfo*), which express the *E. coli* AP endonucleases XthA and Nfo, respectively, using an arabinose-inducible promoter, in the presence (+Arabinose) or absence (−Arabinose) of the inducer. **c**, Same as **a** but using *E. coli* BW25113 mutants with deletions of genes involved in RecABCD or RecJQ DNA repair pathways. Plaque images of one representative experiment from two independent experiments are shown in **a**-**c**.

As endonuclease IV can cleave ssDNA oligonucleotides at the abasic sites generated by Brig1 (Extended Data Fig. 3g), we explored whether other enzymes that participate in base excision repair in *E. coli* could be important for Brig1 immunity in vivo. We tested immunity in hosts lacking either one or both of the two major *E. coli* apurinic/apyrimidinic endonucleases, exonuclease III (XthA) and endonuclease IV^29,30^ (Nfo), the pyrimidine DNA glycosylase-lyase endonuclease III^31^ (Nth) or the abasic site sensor YedK^32^. Deletion of any of the genes encoding these enzymes did not affect T4 plaque-forming unit (PFU) counts in the presence of Brig1 (Extended Data Fig. 8a), suggesting that they are not required for immunity. We also performed the opposite experiment (that is, overexpressing XthA and Nfo to determine whether they enhance Brig1 immunity) and found that neither of the apurinic/apyrimidinic endonucleases provided a further decrease in T4 PFUs (Extended Data Fig. 8b). Finally, we determined that other nucleases, helicases and recombinases involved in recombinational DNA repair—RecBCD, RecQ, RecJ and RecA^33^, which could process DNA ends generated by the sequential activity of Brig1 and host- or phage-encoded apurinic/apyrimidinic endonucleases—did not affect immunity (Extended Data Fig. 8c). Therefore, our data indicate that the major *E. coli* DNA repair enzymes and apurinic/apyrimidinic endonucleases do not have a specialized role in Brig1-mediated anti-phage defence.

## Brig1 immunity against diverse phages

**Fig. 4:**
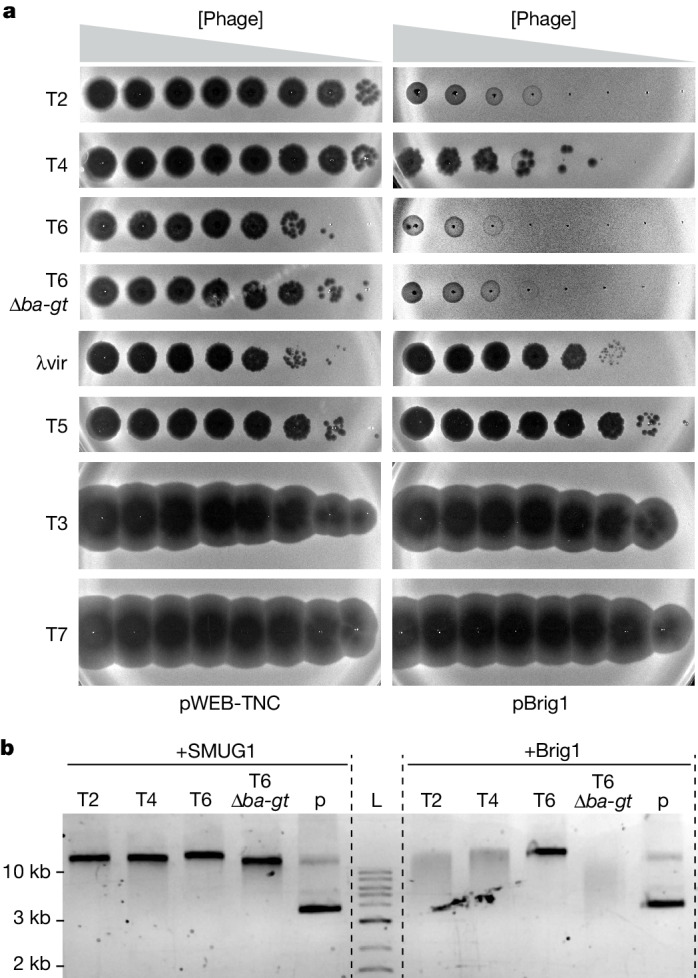
Brig1 provides immunity against diverse phages that carry α-glucosyl-hmC nucleobases. **a**, Tenfold serial dilutions of common coliphages spotted on lawns of *E. coli* EC100 carrying cosmid pWEB-TNC or pBrig1. T6 Δ*ba-gt* lacks the glucosyltransferase that adds the second glucose to α-glucosyl-hmC nucleobases in phage T6. Data are representative of three independent experiments. **b**, Agarose gel electrophoresis of 50 ng of T2, T4, T6, T6 Δ*ba-gt* or pWEB-TNC DNA (p) treated with 10 units of SMUG1 or 100 nM of Brig1 for 30 min at 37 °C. L, DNA size ladder. Data are representative of two independent experiments.

**Extended Data Fig. 9:**
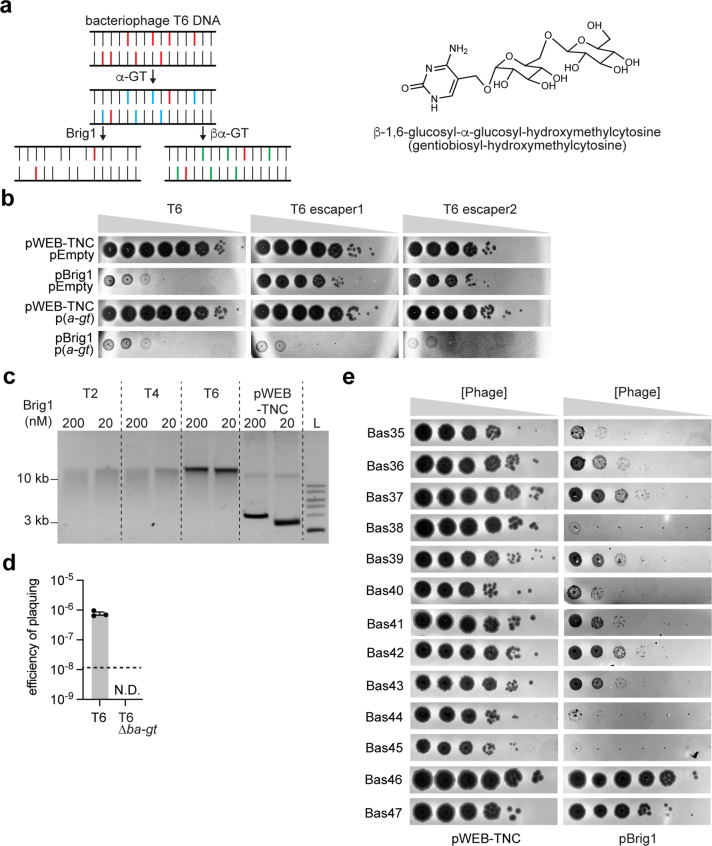
Brig1 immunity against different T-even coliphages. **a**, Schematic of the cytosine modification pathway in phage T6, including a model for Brig1 immunity in which the DNA glycosylase recognizes and excises α-glucosyl-hydroxymethylcytosine nucleobases from the viral genome, before the addition of the second glucosyl group to generate gentiobiosyl-hydroxymethylcytosine. T6 enzymes: Gp42, dCMP hydroxymethylase; Gp56, dCTPase; α-GT, α-glucosyltransferase; βα-GT, β-α glucosyltransferase. **b**, Tenfold serial dilutions of T6, T6 escaper1 and T6 escaper2 phages on lawns of *E. coli* EC100, each lawn carrying cosmid pWEB-TNC or pBrig1, and plasmids pEmpty or p(*a-gt*). **c**, Agarose gel electrophoresis of T2, T4, T6 or pWEB-TNC DNA (125 ng) treated with decreasing concentrations (200, 20 nM) of Brig1 for 30 min at 37 °C. L, DNA size ladder. Data are representative of one experiment. **d**, Efficiency of plaquing of T6 and T6 Δ*ba-gt* phages on lawns of *E. coli* EC100 carrying pBrig1. Mean values are reported for three independent experiments. Error bars report the standard error of the mean (s.e.m.). N.D., no plaques detected; dotted line, limit of detection. **e**, Tenfold serial dilutions of phages from the BASEL collection Bas35-47 spotted on lawns of *E. coli* EC100 carrying pWEB-TNC or pBrig1. Plaque images of one representative experiment from three independent experiments are shown. Source Data

To test the range of phages restricted by Brig1, we infected *E. coli* with seven different coliphages and found that, in addition to T4, phages T2 and T6 were highly sensitive to Brig1 targeting (Fig. 4a). These phages contain α-glucosylated (70% in T2; 3% in T6), but not β-glucosylated hmC sites^12^. In addition, both phage genomes carry β-1,6-glucosyl-α-glucose (gentiobiose; Extended Data Fig. 9a) adducts (T2, 5%; T6, 72%). As the majority of the hmC nucleobases in the T2 genome are α-glucosylated, this phage is, as expected, very sensitive to Brig1 targeting (Fig. 4a). Conversely, as only a small fraction of the T6 genome contains α-glucosyl-hmC, the high susceptibility of this phage to Brig1 is notable (Fig. 4a). To investigate this, we isolated two T6 phages that escaped targeting (Extended Data Fig. 9b) and found that both carried inactivating mutations in the T6 *a-gt* gene (Supplementary Table 1), whose effect was reverted through expression of phage T4 *a-gt* (Extended Data Fig. 9b). We also treated T2, T4 and T6 phage DNA with purified Brig1 (Fig. 4c and Extended Data Fig. 9c). We found that T4 and T2 DNA, but not T6 DNA, was partially degraded during electrophoresis—a result that, as opposed to the in vivo results, correlates with the low fraction of α-glucosyl-hmC nucleobases in the T6 genome. To test this, we deleted the *ba-gt* gene, which encodes β-α glucosyltransferase (βα-GT), the enzyme required to add the second glucose in β-linkage to α-glucosyl-hmC nucleobases and generate gentiobiosyl-hmC (Extended Data Fig. 9a). This phage, T6 Δ*ba-gt*, only carries α-glucosylated hmC nucleobases (presumably in 75% of the cytosines; Extended Data Fig. 9a), and is more susceptible to Brig1 immunity than wild-type T6 (Fig. 4a and Extended Data Fig. 9d). In addition, treatment of T6 Δ*ba-gt* DNA with Brig1 resulted in degradation after running the products on a gel (Fig. 4b). Together, these results suggest that whereas gentiobiose modifications render T6 DNA resistant to Brig1 in vitro, in vivo there is a window during the viral lytic cycle, after the activity of α-GT on newly replicated hmC nucleobases^12,15^ but before the addition of the second glucose by βα-GT, when a large proportion of the hmC nucleobases in T6 are modified only with α-glucose and are therefore susceptible to Brig1 restriction.

Finally, we tested 69 different *E. coli* phages from the BASEL collection^34^ and found that Brig1 provides immunity against Bas35–45, all members of the T-even family that modify their genomes with α-glucosyl-hmC (Extended Data Fig. 9e). By contrast, plaque formation by two other T-even phages within the collection, Bas46 and Bas47, predicted to carry arabinosyl-hmC nucleobases instead of glucosyl-hmC^34,35^, was not affected by Brig1 (Extended Data Fig. 9e). Overall, these data demonstrate that Brig1 restricts a large number of T-even phages that contain α-glucosylated hmC residues in their genomes. Although additional modifications of these nucleobases prevent Brig1 activity, their transient presence during the lytic cycle is sufficient for efficient immunity.

## Homologues of Brig1 also provide immunity

**Fig. 5:**
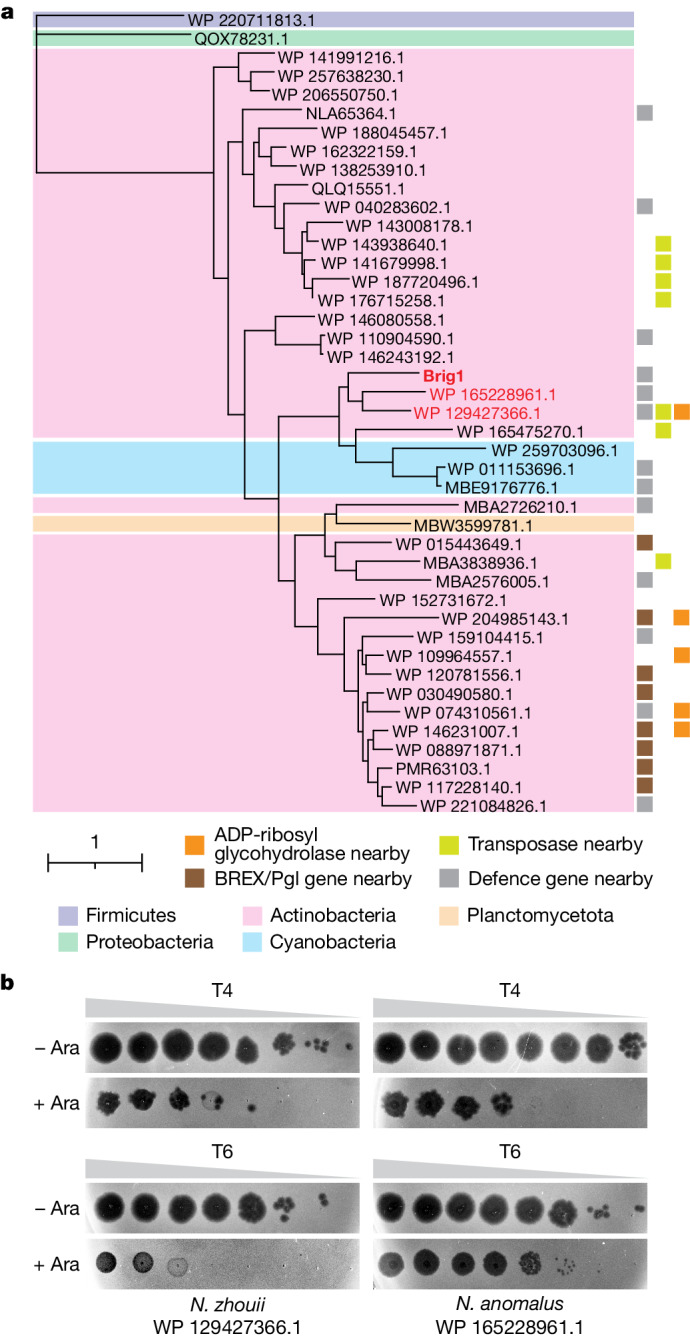
Brig1 homologues provide anti-phage defence. **a**, Maximum likelihood tree of 42 Brig1 homologues (noted by their NCBI protein accession numbers) found in different phyla: Firmicutes (purple background), Proteobacteria (green background), Actinobacteria (pink background), Cyanobacteria (blue background) and Planctomycetota (orange background). Brig1 homologues that provide anti-phage defence against T4 and T6 are indicated in red. Grey squares indicate the presence of putative defence genes in the immediate vicinity (within ten genes upstream and downstream); brown squares indicate BREX/Pgl genes within ten genes upstream and downstream; yellow squares indicate transposases within ten genes upstream and downstream; and orange squares indicate ADP-ribosyl glycohydrolases within ten genes upstream and downstream. **b**, Tenfold serial dilutions of phage T4 or T6 on lawns of *E. coli* EC100 carrying the pAM39 vector to express the Brig1 homologues shown in red in **a** using an arabinose-inducible promoter, in the presence (+Ara) or absence (−Ara) of the inducer. Plaque images of one representative experiment from three independent experiments are shown.

**Extended Data Fig. 10:**
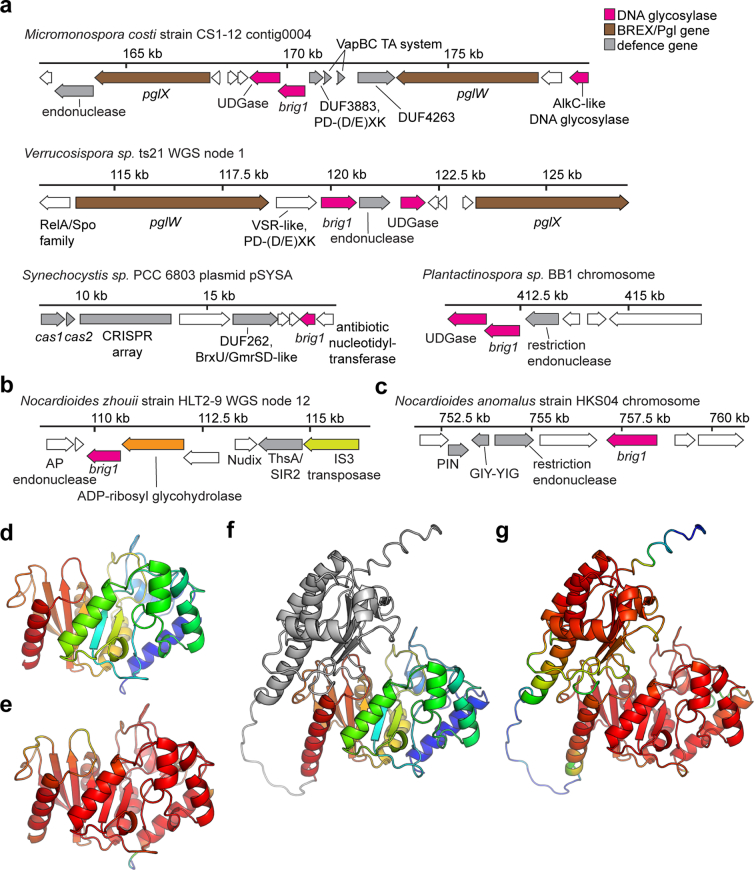
Brig1 homologues. **a**, Gene neighbourhoods of Brig1 homologues found in putative anti-phage defence islands. Brig1 homologues and other DNA glycosylases are shown in magenta, Pgl/BREX genes in brown, and other putative defence genes in grey. **b**, Gene neighbourhood of the Brig1 homologue from *Nocardioides zhouii* showing ADP-ribosyl glycohydrolase, transposase and putative anti-phage defence genes in orange, yellow and grey, respectively. **c**, Same as **b** but for the Brig1 homologue from *Nocardioides anomalus*. **d**, AlphaFold2 structure of the Brig1 homologue from *N. zhouii*, coloured by position (N-terminal, blue; to C-terminal, red). **e**, Same as **d** but coloured by pLDDT (a per-residue model confidence score). Red to blue spectrum represents high to low confidence of secondary structure prediction. **f**, Same as **d** but showing the AlphaFold2 structure of the Brig1 homologue from *N. anomalus*. **g**, Same as **e** but showing the AlphaFold2 structure of the Brig1 homologue from *N. anomalus*.

We used PSI-BLAST to analyse the prevalence of Brig1 in prokaryotic genomes and found 42 non-redundant homologues (annotated on NCBI as hypothetical proteins). Many of these are present within putative anti-phage defence islands (Fig. 5a and Extended Data Fig. 10a), near other annotated anti-phage immunity genes^4^ (Supplementary Data File 1). Most of the Brig1 homologues currently available in genetic databases are found in Actinobacteria (Fig. 5a). We found that two closely related Brig1 homologues, both present in *Nocardioides*, provided immunity against T4 and T6 (Fig. 5a,b). These homologues are present in putative defence islands (Extended Data Fig. 10b,c, and Supplementary Data File 1), with the one harboured by *Nocardioides zhouii* being located in a similar genomic neighbourhood as *brig1*—that is, adjacent to a predicted ADP-ribosyl glycohydrolase and near a ThsA-like SIR2-domain protein (Extended Data Fig. 10b). Both homologues share around 50% amino acid identity with Brig1 (Supplementary Data File 1) and a high level of predicted structural similarity (Extended Data Fig. 10d–g).

## Discussion

Here we have developed a functional screen for the discovery of prokaryotic anti-phage defence systems from eDNA. Many novel defence systems have been uncovered through bioinformatic exploration of deposited DNA sequences^4–6,36^. Although this approach has been highly effective, success depends on the availability of genomic data. One recent study performed a functional screen on genomic libraries of different *E. coli* strains^9^, a method that has the benefit of an almost guaranteed expression of the library inserts. The sequence space screened, however, was limited to the genetic content of this bacterium. The use of eDNA libraries for the isolation of clones with antiviral properties has the caveat that many genes may not be expressed in a heterologous host. However, such eDNA libraries enable exploration of the microbial dark matter^10,37^. They provide access to the genetic information of diverse, yet to be discovered, organisms, that do not need to be cultured and that in principle can be isolated from any environment of our planet^38,39^. Finally, our screen has the advantage of unearthing not only individual antiviral systems, but also entire defence islands and/or mobile genetic elements, which may reveal new insights into prokaryotic host–virus conflicts in nature (see also Supplementary Discussion).

Our eDNA library screen yielded a prokaryotic defence island containing a DNA glycosylase, Brig1, that provides immunity in *E. coli* against T-even bacteriophages by excising α-glucosyl-hmC nucleobases in the viral DNA. Brig1 did not restrict the propagation of T4 Δ*a-gt* nor Bas46-47 phages, whose genomes lack α-glucosyl-hmC and instead contain β-glucosyl-hmC and arabinosyl-hmC nucleobases, respectively. The enzyme also did not degrade T6 phage DNA, which primarily harbours gentiobiosyl-hmC. Therefore, it is conceivable that T-even phages have diversified their hmC modification patterns to avoid restriction by DNA glycosylases involved in anti-phage defence such as Brig1. If so, the arms race between phages that modify their DNA and their hosts most probably resulted in the evolution of a larger family of Brig DNA glycosylases with activity against different hmC nucleobases, which probably includes some of the Brig1 homologues that we found associated with other defence genes but did not provide immunity against T4 and T6 (Extended Data Fig. 10a).

Our assays with oligonucleotide substrates showed that the phosphate backbone of ssDNA and dsDNA substrates containing a single α-glucosyl-hmC nucleobase or dsDNA substrates containing two α-glucosyl-hmC nucleobases on opposite strands remained largely uncleaved despite overnight incubation with large amounts of Brig1 protein (Fig. 3 and Extended Data Fig. 5). We therefore propose that Brig1, like members of the uracil DNA glycosylase superfamily^21^, is primarily a monofunctional DNA glycosylase rather than a bifunctional glycosylase-lyase that would also nick the DNA phosphate backbone upon base excision^21^. We believe that Brig1 activity disrupts the lytic cycle of T-even viruses by generating abasic sites throughout the viral genome that: (1) impede phage transcription and/or replication; (2) lead to spontaneous hydrolysis of the phosphate backbone at the highly reactive abasic sites; and/or (3) result in DNA interstrand crosslinks and DNA–protein crosslinks owing to abasic site reactivity^25^. In addition, given that Brig1 can target ssDNA substrates, it would be possible for this enzyme to attack ssDNA intermediates that form during rolling-circle replication of T-even phages^26^. Notably, we observed weak base excision activity after treating a uracil-containing ssDNA oligonucleotide with high concentrations of Brig1 (Extended Data Fig. 3h and Extended Data Fig. 4c). This weak secondary activity on uracil suggests Brig1 has probably evolved from members of the uracil DNA glycosylase superfamily.

Given that there is no evidence for the misincorporation of α-glucosyl-hmC into bacterial DNA in the absence of phage infection, it is unlikely that Brig1 would participate in a host base excision repair pathway dedicated to the removal of these nucleobases. On the contrary, we believe that Brig1 is a bona fide antiviral effector. Supporting this idea, the *a-gt* gene, responsible for the generation of α-glucosyl-hmC nucleobases, is widespread across phages infecting diverse hosts (Supplementary Data File 1), and we found that Brig1 provided immunity against 11 out of 69 phages from the BASEL collection (Extended Data Fig. 9e). Furthermore, many Brig1 homologues are part of anti-phage defence islands, frequently associated with BREX/Pgl genes, but also with toxin–antitoxin cassettes, restriction endonucleases and CRISPR–Cas systems (Extended Data Fig. 10a–c and Supplementary Data File 1). In these genetic contexts, Brig1 provides an additional layer of immunity against phages containing α-glucosyl-hmC that cannot be targeted by other systems present in the anti-phage defence islands, such as CRISPR–Cas and restriction endonucleases^16,40^ and possibly BREX immunity. Although the exact mechanism used by BREX systems to restrict phage infection is unknown, it was recently shown that wild-type T4, but not an *a-gt*/*b-gt* double mutant phage that lacks hmC glucosylation, evades BREX immunity in *E. coli* HS^41^, suggesting that BREX systems are inhibited by glucosylated nucleobases. In hosts harbouring defence islands with restriction enzymes, CRISPR or BREX in addition to Brig1, phages targeted by the latter will not be able to escape through mutations in *a-gt* that eliminate α-glucosylation of the viral DNA, since they will become susceptible to the defence mechanisms that target non-glucosylated DNA. The next step of the arms race could involve a change in the nucleobase modification of the phage to avoid Brig1 excision and regain virulence. Our study enables exploration of this immunity mechanism with an alternative mode of attacking viral DNA that involves base excision rather than the direct cleavage of sugar–phosphate backbones.

## Methods

### Bacterial strains and growth conditions

Cultivation of *E. coli* EC100 (Lucigen), *E. coli* K-12 MG1655^42^, *E. coli* K-12 BW25113^43^ and all other *E. coli* strains used in this study were carried out in lysogeny broth (LB) at 37 °C with shaking. Overnight cultures were inoculated from single bacterial colonies. Wherever applicable, media were supplemented with chloramphenicol at 12.5 μg ml^−1^ (for cosmids) or 25 μg ml^−1^ (for plasmids), spectinomycin at 50 μg ml^−1^, kanamycin at 50 μg ml^−1^, ampicillin or carbenicillin at 100 μg ml^−1^, and/or tetracycline at 5 μg ml^−1^ to ensure cosmid or plasmid maintenance. *E. coli* Keio knockout strains were obtained from the Coli Genetic Stock Center at Yale University^44^. The type I-E CRISPR interference strain *E. coli* K-12 MG1655 ACT-01 was a gift from C. A. Voight^45^. Miniprepped plasmids (prepared by QIAprep Spin Miniprep Kit, QIAGEN, 27106) were cloned into chemically competent *E. coli* EC100 cells (Lucigen), electrocompetent *E. coli* EC100 cells (Lucigen) or rubidium chloride (RbCl_2_) chemically competent *E. coli* K-12 MG1655 cells. For *E. coli* K-12 BW25113 and Keio knockout strains, protein purification strains, and strains with two plasmid combinations, existing strains were first made electrocompetent and then transformed with plasmid through electroporation (1 mm Bio-Rad Gene Pulser cuvette at 1.8 kV). The list of strains used in this study are available in Supplementary Data File 2.

### Plasmid construction

For plasmid construction, refer to Supplementary Data File 2.

### Gibson assembly

For Gibson assemblies^46^, 25–100 ng of the largest dsDNA fragment was combined with equimolar volumes of the smaller fragment(s) in a total volume of 5 μl in nuclease-free water. Reaction mixtures were prepared on ice and mixed with 15 μl of Gibson assembly master mix, pipette mixed and incubated at 50 °C for 1 h in a thermal cycler. Gibson reactions were transformed into chemically competent *E. coli* EC100 cells (Lucigen) or RbCl_2_ chemically competent *E. coli* K-12 MG1655 cells by mixing 5 μl of Gibson reaction with 50 μl cells and following a standard transformation protocol for chemically competent cells.

### Oligonucleotide cloning

Oligonucleotide cloning was used to create a repeat-spacer-repeat CRISPR array with a desired spacer following a previously described protocol^47^. In brief, we used a BsaI restriction digest cloning approach. Parent type II-A CRISPR array-containing plasmids with a repeat-spacer-repeat carried a 30 bp spacer sequence with two BsaI cut sites at either end (pCas9)^47^. To set up the BsaI plasmid digest, we mixed 42 μl of the parent CRISPR plasmid (40–60 ng μl^−1^) with 6 μl BsaI-HF (NEB, R3535L), 6 μl NEB CutSmart buffer and 6 μl nuclease-free water. The restriction digest reaction was incubated at 37 °C for approximately 6 h. Two IDT oligonucleotides comprised the type II-A CRISPR spacer to be inserted into the BsaI cut plasmid CRISPR array: a ‘top’ strand oligonucleotide with sequence 5′-AAAC-(30 bp spacer)-G-3′ and a ‘bottom’ strand oligonucleotide with sequence 5′-AAAAC-(30 bp spacer reverse complement)−3′. For oligonucleotide cloning of type I-E spacers into pACYC184-TypeIE*spcNT*, the top strand oligonucleotide had sequence 5′-ACCG-(32 bp spacer)−3′ and the bottom strand oligonucleotide had sequence 5′-ACTC-(32 bp spacer reverse complement)−3′. The two oligonucleotides were phosphorylated with T4 polynucleotide kinase (NEB, M0201S) in a 50 μl reaction: 1.5 μl 100 μM top oligonucleotide, 1.5 μl 100 μM bottom oligonucleotide, 41 μl nuclease-free water, 5 μl T4 DNA ligase reaction buffer (NEB, B0202S), 1 μl T4 polynucleotide kinase (NEB, M0201S). The reaction was incubated at 37 °C for 1 h in a thermal cycler. After phosphorylation, oligonucleotides were annealed by adding 2.5 μl of 1 M sodium chloride (Fisher Scientific, S271-3) solution to the 50 μl reaction and incubating for 5 min at 98 °C and then allowing the reaction to gradually cool to room temperature (approximately 2 h). The annealed oligonucleotides were diluted 1:10 in nuclease-free water and ligated into the BsaI-digested plasmid in a 20 μl reaction: 10 μl BsaI-digested plasmid, 6 μl nuclease-free water, 1 μl 1:10 diluted annealed oligonucleotides, 5 μl T4 DNA ligase reaction buffer (NEB, B0202S), 1 μl T4 DNA ligase (NEB, M0202M). The ligation reaction was performed at room temperature overnight. The next day, 5 μl of the ligation reaction was transformed into 50 μl of chemically competent *E. coli* EC100 cells (Lucigen) or electrocompetent ACT-01 cells following dialysis, and colonies were confirmed by PCR the next day. The list of CRISPR spacers generated by BsaI cloning and used in this study are available in Supplementary Data File 2.

### Strain construction

λ Red recombineering was used to generate the *E. coli* K-12 BW25113 Δ*xthA* Δ*nfo* strain. An overnight culture of the *E. coli* K-12 BW25113 Δ*nfo* Keio strain carrying the pAM38(*red*) plasmid with chloramphenicol resistance was diluted and grown to OD_600_ ~ 0.3 and then induced with 0.2% l-arabinose until OD_600_ ~ 1–1.2. Cells were made electrocompetent by washing twice with cold water and electroporated (1 mm Bio-Rad Gene Pulser cuvette at 1.8 kV) with a PCR product carrying a *xthA:tetR* gene replacement matching the *xthA:kanR* gene replacement found in the *E. coli* K-12 BW25113 Δ*xthA* Keio strain, with ~50 bp homology upstream and downstream of the *xthA* locus in the PCR product. After ~2 h of recovery, cells were plated on LB agar plates with kanamycin at 50 μg ml^−1^ and tetracycline at 5 μg ml^−1^ to select for double mutants. Double knockouts were confirmed by PCR. After confirmation, strains were grown overnight in LB with kanamycin at 50 μg ml^−1^ and tetracycline at 5 μg ml^−1^ (but no chloramphenicol which selects for the plasmid) and with 0.2% l-arabinose induction. Without antibiotic selection, induced plasmid was rapidly lost due to toxicity from λ Red overexpression. Strains were frozen at −80 °C (900 μl culture + 100 μl DMSO) and struck out on appropriate antibiotic plates to confirm both double knockouts and loss of the recombineering plasmid.

### Preparation of phage stocks

λvir, T4 and T7 were gifts from B. Levin. T2, T3, T5 and T6 phages were purchased from ATCC. Phages were first grown up in 10 ml cultures of exponentially growing *E. coli* K-12 MG1655 or EC100 cells at OD_600_ ~ 0.3. The phage-added cultures were incubated at 37 °C with shaking overnight. Tubes were then spun down at 15,000*g* for 10 min at 4 °C. Phage-containing supernatants were filtered using Acrodisc 13 mm SUPOR 0.45 μm syringe filters (Pall, 4604) into 15 ml conical tubes and supernatants frozen down as phage stocks at −80 °C (900 μl filtered supernatant + 100 μl DMSO). To grow up a phage stock for plaquing assays and other experiments, a pipette tip was used to scrape off a tiny portion of a frozen phage stock, which was then resuspended in 20 μl LB medium. Serial dilutions were prepared from the resuspended phage and spotted on a fresh LB top agar (LB broth Lennox base, 0.5% agar) lawn of *E. coli* EC100 in LB agar. The plate was incubated at 37 °C overnight after drying at room temperature for 25 min. The next day a single phage plaque was picked from the top agar lawn using a P20 pipette set to 15 μl and resuspended in a 10 ml culture of exponentially growing *E. coli* EC100 at OD_600_ ~ 0.3. The phage-added culture was incubated at 37 °C with shaking overnight. The tube was spun down the next day at 15,000*g* for 10 min at 4 °C. The phage-containing supernatant was filtered using an Acrodisc 13 mm SUPOR 0.45 μm syringe filter (Pall, 4604) into a 15 ml conical tube. All final phage stocks were titred on top agar lawns of *E. coli* EC100 and stored at 4 °C.

To grow phage stocks of Brig1 escaper phages, single plaques formed by T4 or T6 phages on lawns of pBrig1-carrying EC100 cells were picked using a P20 pipette and resuspended in 20 μl LB medium. Serial dilutions were prepared from the resuspended phage and spotted on a fresh LB top agar lawn of *E. coli* EC100 carrying pBrig1 to maintain selection of the escaper phage. The plate was incubated at 37 °C overnight after drying at room temperature for 25 min. The next day a single phage plaque was picked from the top agar lawn using a P20 pipette set to 15 μl and resuspended in a 10 ml culture of exponentially growing OD_600_ ~ 0.3 *E. coli* EC100 carrying pBrig1 for continued selection. The phage-added culture was incubated at 37 °C with shaking overnight and filtered the next day as described earlier to generate the escaper phage stock. Final phage stocks were titred on top agar lawns of *E. coli* EC100 and stored at 4 °C. The list of phages used in this study are available in Supplementary Data File 2.

### Generation of mutant phage stocks

T4 and T6 phage stocks were used to construct T4 Δ*a-gt*, T4 Δ*b-gt*, T4 Δ*alc* Δ*denB* Δ*gp56*, T4(C) and T6 Δ*ba-gt* mutant phage stocks. In each case, a culture of *E. coli* EC100 cells carrying a recombinant pUT18C-based plasmid was grown overnight at 37 °C with shaking in 10 ml LB supplemented with 100 μg ml^−1^ carbenicillin. The pUT18C plasmid contained a cloned segment of phage T4 or T6 DNA with the desired gene deleted and ~750–1000 bp homology arms flanking the deleted genic region on either side. The overnight culture was diluted 1:50 in 10 ml LB medium supplemented with 100 μg ml^−1^ carbenicillin. After approximately 1 h of culture growth, OD_600_ was measured for the culture and confirmed to be between 0.2 and 0.4. The 10 ml culture was then infected with 2 μl of T4 or T6 phage stock and grown overnight at 37 °C with shaking to allow wild-type phages to recombine with the plasmid. The next day, the tube was spun down at 15,000*g* for 10 min at 4 °C. The phage-containing supernatant was filtered using an Acrodisc 13 mm SUPOR 0.45 μm syringe filter (Pall, 4604) into a 15 ml conical tube.

Serial dilutions of recombinant phage were prepared and spotted on a fresh top agar lawn of *E. coli* EC100 containing a pCas9 plasmid in LB agar supplemented with 25 μg ml^−1^ chloramphenicol. The pCas9 plasmid carried a type II-A CRISPR spacer targeting the phage gene that was deleted to select specifically for recombinant phage with the desired deletion. Top agar plates were incubated at 37 °C overnight after drying at room temperature for 25 min. The next day multiple phage plaques were picked from the top agar lawn using a P20 pipette set to 15 μl and resuspended in 20 μl LB medium. Five microlitres of the resuspend phage plaques were boiled in 15 μl colony lysis buffer^48^ at 98 °C for 15 min and then PCR checked to confirm that the desired gene was deleted, either with the deletion carried on the pUT18C recombinant plasmid or a de novo CRISPR-generated deletion that eliminated the appropriate gene. Serial dilutions were prepared for 1–2 correct phage plaques, which were then replaqued onto top agar lawns of pCas9 selection strains and incubated overnight at 37 °C for stringent selection. The next day, a single phage plaque was picked from the top agar lawn using a P20 pipette set to 15 μl and pipetted directly into an OD_600_ ~ 0.2–0.4 exponentially growing culture that maintained the same selection for the mutant phage. The phage-infected culture was grown overnight at 37 °C with shaking. The next day, the tube was spun down at 15,000*g* for 10 min at 4 °C. The phage-containing supernatant was filtered using an Acrodisc 13 mm SUPOR 0.45 μm syringe filter (Pall, 4604) into a 15 ml conical tube. In some cases, an arabinose-inducible type I-E CRISPR–Cas expressing *E. coli* MG1655 strain, ACT-01, with a pACYC184-based plasmid expressing an arabinose-inducible type I-E CRISPR spacer was used to select for the recombinant phage. In these instances, 0.2% l-arabinose was included in all media for proper phage selection through type I-E CRISPR–Cas targeting. To make the T4 Δ*a-gt* phage, instead of CRISPR selection, *E. coli* EC100/pBrig1 was used to select for the pUT18C-recombined phage. PCR and Sanger sequencing confirmed the desired in-frame deletion of *a-gt* in the mutant phage, matching the exact deletion carried on the pUT18C-d*a-gt* recombination plasmid.

To make the T4(+β-GT) phage, which is T4 phage carrying a higher-than-normal fraction of β-glucosyl-hmC nucleobases, wild-type T4 was passaged through *E. coli* EC100 carrying the plasmid p(*b-gt*), which overexpresses T4 β-GT under 1 mM isopropyl-β-d-thiogalactopyranoside (IPTG) induction. An overnight culture of *E. coli* EC100/p(*b-gt*) was diluted 1:50 in 10 ml LB medium supplemented with 50 μg ml^−1^ spectinomycin and 1 mM IPTG. After approximately 1 h 15 min of culture growth, OD_600_ was measured for the culture and confirmed to be between 0.2-0.4. The 10 ml culture was then infected with 2 μl of wild-type T4 phage stock and grown overnight at 37 °C with shaking. The next day, the tube was spun down at 15,000*g* for 10 min at 4 °C. The phage-containing supernatant was filtered using an Acrodisc 13 mm SUPOR 0.45 μm syringe filter (Pall, 4604) into a 15 ml conical tube.

Please refer to Supplementary Data File 2 for the plasmids used to generate each mutant phage. All final phage stocks were titred on top agar lawns of *E. coli* EC100 and stored at 4 °C. The list of phages used in this study are also available in Supplementary Data File 2.

### Plaque assays and efficiency of plaquing analysis

Overnight cultures were launched from single colonies in 3 ml of LB medium supplemented with appropriate antibiotic(s). Top agar lawns of *E. coli* were prepared by mixing 100 μl of overnight culture with 6 ml of LB top agar (LB broth Lennox base, 0.5% agar) supplemented with appropriate antibiotic(s). Top agar mixtures were poured over LB agar in 10 cm plates supplemented with appropriate antibiotic(s). Where necessary, 0.2% l-arabinose was included in the overnight media as well as in the LB top agar and the LB agar plate. Plates were dried at room temperature, partially open by a sterilizing flame, for 25 min for the top agar to solidify. Serial dilutions of phage stock were prepared and spotted on the top agar after drying. For imaging of plaque assays, 2.5 μl of each phage dilution was spotted on top agar using a multichannel pipette. For quantification of phage titres, efficiency of plaquing, and isolation of single phage plaques for phage DNA sequencing, 3–3.5 μl of each phage dilution was spotted on top agar using a multichannel pipette and the plate was tilted to allow phage spots to drip down the plate for easier quantification and isolation of single plaques. In all cases, plates were incubated at 37 °C overnight after drying at room temperature for 25 min or until the plates were completely dry. Overnight plaque assays were imaged the next day (~16–24 h after infection) using the FluorChem HD2 system (ProteinSimple). Plaque assay images were all auto-contrasted using Adobe Photoshop to give clearer images. In some cases, image brightness was enhanced further using Adobe Photoshop for better visualization of phage spots. Plaque assays with BASEL phages reported in Extended Data Fig. 9e were performed in larger 15 cm plates of LB agar supplemented with 12.5 μg ml^−1^ chloramphenicol, to allow for plaquing of up to ten different phages on a single lawn. Here, the protocol was performed exactly as above, except with scaled up volumes: 300 μl of overnight culture was mixed with 15 ml of LB top agar supplemented with 12.5 μg ml^−1^ chloramphenicol. As before, 2.5 μl of each phage dilution was spotted on top agar using a multichannel pipette.

In Extended Data Fig. 2i, efficiency of plaquing was quantified as the number of plaques formed by the phage on an *E. coli* EC100/pBrig1 (targeting) lawn divided by the number of plaques formed by the same phage on an *E. coli* EC100/pWEB-TNC (control) lawn. In Extended Data Fig. 9c, to quantify phage plaques of T6 and T6 Δ*ba-gt* formed on *E. coli* EC100/pBrig1 (targeting) lawns, infections were spread out across the entire top agar lawns to accurately count individual plaque-forming units (PFUs). To this end, 100 μl of phage stock normalized to ~1 × 10^6^ PFU μl^−1^ (so ~10^8^ PFUs total) was mixed with 100 μl of overnight culture and then mixed with 6 ml LB top agar (with 12.5 μg ml^−1^ chloramphenicol) and subsequently poured over an LB agar plate, supplemented with 12.5 μg ml^−1^ chloramphenicol. Top agar plates were incubated at 37 °C overnight after drying at room temperature for 25 min. The next day, single plaques were counted across the entire top agar lawn. To accurately determine the total PFUs added of each phage, plaquing of serial dilutions of the ~1 × 10^6^ PFU μl^−1^ normalized phage stocks was performed following the standard procedure of a plaque assay outlined above, using 3.5 μl drips of each phage dilution to facilitate more precise quantification of phage titres. Efficiency of plaquing was quantified as the total number of plaques formed by the phage across an entire *E. coli* EC100/pBrig1 (targeting) lawn divided by the experimentally estimated total number of PFUs added.

### Functional selection of a T4-resistant clone in the AZ52 soil DNA library in *E. coli*

The DNA library we used was generated in an earlier study using DNA extracted from an arid soil sample collected in Arizona^11^. The library, AZ52, is comprised of large ~40 kb DNA fragments from soil microorganisms cloned into a pWEB-TNC cosmid. The insert-carrying cosmids were transformed into *E. coli* EC100 cells (Lucigen), generating a soil DNA library with approximately 20 million clones, divided into megapools carrying roughly 1.25 million clones each.

Each clone within the library houses a cosmid with a soil DNA insert, which carries genes from soil-derived microorganisms. Soil-derived genes can therefore be expressed heterologously in our library system. We performed our functional screen using the coliphage T4. To grow up libraries, we scraped frozen library stocks of *E. coli* EC100 carrying megapools 3–16 of the AZ52 DNA library into separate tubes with 10 ml LB supplemented with 12.5 μg ml^−1^ chloramphenicol and grew cultures overnight at 37 °C with shaking. The next day, we infected *E. coli* EC100 overnight cultures with T4 at a multiplicity of infection (MOI) of 10, high enough to kill almost all clones without bona fide immunity. Infections were performed in 6 ml LB top agar with 500 μl of overnight stationary culture mixed with phage at MOI 10 on LB agar plates, supplemented with 12.5 μg ml^−1^ chloramphenicol. We incubated plates at 37 °C for 36–48 h and then inspected surviving colonies within top agar infections. We found that only megapool 4 showed an increased number of surviving colonies upon T4 infection compared to an infection of *E. coli* EC100 cells carrying an empty pWEB-TNC cosmid (control).

As cells may survive T4 infection due to mutations within the *E. coli* host that prevent phage infection and not due to immunity genes carried within the soil DNA cosmids, we wanted to enrich for true immunity genes carried on cosmids. To eliminate false positive clones, we extracted pooled cosmid DNA from the surviving colonies on the enriched plate. To do this, we scraped top agar with surviving colonies into a 50 ml conical tube, melted the top agar in a 98 °C heating block for 10–15 min until the top agar was completely melted, and then centrifuged the tube at ~4,000*g* for 5 min at room temperature to collect a cell pellet from which surviving cosmids were isolated using the QIAprep Spin Miniprep Kit (QIAGEN, 27106). The miniprepped cosmid pool was then transformed into 50 μl of electrocompetent *E. coli* EC100 cells (Lucigen) through electroporation (1 mm Bio-Rad Gene Pulser cuvette at 1.8 kV) and recovered in 1 ml SOC medium. After 1.5 h of recovery, cells were assayed for transformation efficiency by pipetting tenfold serial dilutions of the transformation culture on to an LB agar plate supplemented with 12.5 μg ml^−1^ chloramphenicol. While the plate was grown overnight at 37 °C, the remaining transformation culture was stored overnight at 4 °C. The next day, based on the calculated transformation efficiency, the transformation culture was spread onto ten 15 cm LB agar plates supplemented with 12.5 μg ml^−1^ chloramphenicol, plating for ~30,000 colonies on each plate, for a total of ~300,000 colonies. Plates were incubated overnight at 37 °C and the next day colonies from all ten plates were scraped into 20 ml LB, vortexed and inverted to mix, and then diluted to OD_600_ = 10. The OD_600_ = 10 colony mixture was then mixed 1:1 with 50% glycerol to make a −80 °C freezer stock of a 1× phage-enriched DNA library for AZ52 megapool 4. This library was then grown up for re-infection with T4 and the steps described above were repeated two more times to generate a freezer stock of a 3× phage-enriched DNA library for AZ52 megapool 4.

We sampled colonies from the 3×-enriched library for anti-phage immunity by streaking the library to single colonies on an LB agar plate supplemented with 12.5 μg ml^−1^ chloramphenicol. Sixteen single colonies were grown overnight in LB supplemented with 12.5 μg ml^−1^ chloramphenicol at 37 °C with shaking. Colonies were assayed for anti-phage immunity using plaque assays (described above) with phages λvir and T4. Of the sixteen colonies, twelve were found to carry immunity against T4 and none against λvir. Cosmids were isolated from the twelve T4-resistant clones using the QIAprep Spin Miniprep Kit (QIAGEN, 27106) and sent for Sanger sequencing by Genewiz/Azenta using the universal primers T7 and M13F40, which flank the metagenomic DNA insert within the pWEB-TNC cosmid. Sequencing the T4-resistant cosmids revealed they all contained the same metagenomic DNA insert, suggesting that they all originated from the same T4-resistant library clone. One of the twelve T4-resistant clones was frozen at −80 °C (900 μl culture + 100 μl DMSO) for use in future experiments.

### Cosmid sequencing, assembly and gene annotation

Cosmid DNA was extracted using the QIAprep Spin Miniprep Kit (QIAGEN, 27106). DNA was sequenced using the Nextera XT DNA Library Preparation Kit (Illumina, FC-131-1024). Paired-end 2 × 75 bp sequencing was conducted using the 150-cycle MiSeq Reagent Kit v3 (Illumina, MS-102-3001) on the Illumina MiSeq platform. Geneious Prime was used to assemble the cosmid genome, using the Geneious assembler (medium sensitivity/fast) on 100,000 paired-end DNA sequencing reads. The sequence of the cosmid harbouring Brig1 was deposited on GenBank, accession number OR880862. SnapGene was used to predict ORFs with ATG or GTG start codons (minimum length: 50 amino acids) within the metagenomic DNA insert of the assembled cosmid genome. Predicted ORFs were then run through NCBI PSI-BLAST (https://blast.ncbi.nlm.nih.gov/Blast.cgi?PAGE=Proteins) and HHpred^49,50^ (https://toolkit.tuebingen.mpg.de/tools/hhpred) to ascertain protein function where possible. Side-by-side genome annotation was also performed using the Bacterial and Viral Bioinformatics Resource Center (BV-BRC) Genome Annotation Service (https://www.bv-brc.org/app/Annotation), with the annotation recipe for Bacteria/Archaea and the taxonomy name set to *Nocardiodes*, taxonomy ID 1839. Defence genes and systems were identified using DefenseFinder^51,52^ (https://defense-finder.mdmparis-lab.com/) and the Prokaryotic Antiviral Defence LOCator (PADLOC)^53,54^ (https://padloc.otago.ac.nz/padloc/).

### Subcloning of the T4-resistant cosmid to identify the T4 anti-phage system

To identify the immunity gene(s) in our cosmid, we subcloned four DNA fragments (A–D) that span the entire length of the metagenomic insert sequence. DNA fragments were amplified using 10 ng of cosmid DNA as template for PCR amplification using Phusion High-Fidelity DNA Polymerase (Thermo Scientific, F530L) with 1 M betaine (Sigma-Aldrich, B0300) and 1 μl DMSO in a 50 μl PCR reaction. Fragments were cloned into PCR-amplified pWEB-TNC cosmid backbones using NEBuilder HiFi DNA Assembly Master Mix (NEB, E2621L). NEBuilder HiFi DNA assembly was carried out at 50 °C in a thermal cycler for 4 h, and then 5 μl of the assembly reaction was transformed into 50 μl of chemically competent *E. coli* EC100 cells (Lucigen). Cells were incubated on ice for 30 min, heat shocked in a 42 °C water bath for 30 s, placed back on ice for 2 min and then recovered in 250 μl SOC for 2 h. Cells were then plated on LB agar supplemented with 12.5 μg ml^−1^ chloramphenicol and incubated overnight at 37 °C. The next day, 8 colonies were picked, grown overnight in LB supplemented with 12.5 μg ml^−1^ chloramphenicol and their cosmids miniprepped the next day using the QIAprep Spin Miniprep Kit (QIAGEN, 27106). Miniprepped cosmids were sent for Sanger sequencing by Genewiz/Azenta using the universal primers T7 and M13F40, which flank the subcloned DNA fragment inserted into the pWEB-TNC cosmid backbone. Colonies that harboured cosmids with correct insert fragments were then assayed for immunity against phage T4 using plaque assays (see above). Plaque assays identified Fragment D as the fragment harbouring anti-T4 immunity. Fragment D was further subdivided into Fragments D1, D2 and D3, cloned and tested for immunity as described above. Fragment D3, containing a three-gene operon, was identified as the minimal DNA fragment carrying anti-T4 immunity. To determine the gene or genes responsible within the Fragment D3 operon, we generated six cosmid constructs (D3-1 to D3-6) containing different numbers and combinations of the three genes within the operon, each time being driven by the same promoter upstream of the first gene within the operon. These constructs were then tested using plaque assays to identify the gene within the operon that conveyed anti-T4 immunity.

### NCBI blastn of the T4-resistant metagenomic DNA sequence

To identify possible organisms that our metagenomic DNA comes from, we performed a nucleotide BLAST on NCBI using the algorithm for somewhat similar sequences (blastn) (https://blast.ncbi.nlm.nih.gov/Blast.cgi?PROGRAM=blastn&BLAST_SPEC=GeoBlast&PAGE_TYPE=BlastSearch). We performed blastn on the DNA sequences of Fragments C and D (see above and Extended Data Fig. 1c).

### T4 phage adsorption assay

Overnight cultures of *E. coli* EC100 cells carrying pWEB-TNC or pBrig1 were diluted 1:50 in 10 ml LB medium supplemented with 12.5 μg ml^−1^ chloramphenicol. After 1 h 15 min of culture growth, OD_600_ was measured for each culture and normalized to OD_600_ = 0.3. Cultures were then infected with T4 at MOI 0.01 and incubated at 37 °C with shaking for 50 min. A 10 ml bacteria-free, media-only control (LB + 12.5 μg ml^−1^ chloramphenicol) was mixed with the same volume of T4 and incubated alongside the cultures at 37 °C with shaking for 50 min. Phage-infected cultures were sampled at the following time points: 0-, 10-, 20-, 30-, 40- and 50-min post-infection. At each time point, 400 μl was collected from each phage-infected culture and spun down in 1.5 ml Eppendorf tubes at 15,000 rpm for 2 min in a tabletop microcentrifuge at 4 °C. Phage-containing supernatants were filtered using Acrodisc 13 mm SUPOR 0.45 μM syringe filters (Pall, 4604) into fresh 1.5 ml Eppendorf tubes. Filtered phage supernatants were used to prepare two sets of serial dilutions to estimate phage titres on fresh top agar lawns of *E. coli* K-12 MG1655. Top agar plates were incubated at 37 °C. The next day, phage plaques were counted to determine phage titres at each time point. The experiment was repeated another two times in this manner for three independent biological replicates.

### qPCR of phage DNA replication

To quantify phage DNA replication within an infected *E. coli* cell, overnight cultures of *E. coli* EC100 cells carrying pWEB-TNC or pBrig1 were diluted 1:50 in 50 ml of LB medium supplemented with 12.5 μg ml^−1^ chloramphenicol. After 1 h 15 min of growth, OD_600_ was measured, and the culture was normalized to OD_600_ = 0.3. 700 μl of culture was dispensed between multiple 1.5 ml Eppendorf tubes, corresponding to three replicates and multiple time points for each infection being monitored. These 700 μl cultures were infected with phage T4 at MOI 1 and incubated at 37 °C with shaking for specified time points. At each time point, samples were removed from the incubator and tubes spun down at 15,000 rpm for 1 min in a tabletop microcentrifuge at 4 °C. Supernatants were removed and cell pellets immediately frozen down at −80 °C for DNA extraction later. Additionally, 1–3 uninfected tubes for cells carrying pWEB-TNC or pBrig1 were also prepared for DNA extraction as no-phage controls for qPCR.

Total DNA was extracted from frozen *E. coli* cell pellets using the Promega Wizard Genomic DNA Purification Kit (Promega, A1125) following the protocol for Gram-negative bacteria. Extracted DNA was quantified using the Qubit dsDNA HS Assay Kit and each sample was normalized to 4 ng μl^−1^. A total of 32 ng DNA was used as input for qPCR, performed using Fast SYBR Green Master Mix (Applied Biosystems, 4385612) and the QuantStudio 3 Real-Time PCR System (Applied Biosystems) with primer pairs AA870/AA871 (T4 *gp43* target), AA872/AA873 (T4 *gp34* target) and AA387/AA388 (*E. coli* K-12 MG1655 *dxs* control). For qPCR data analysis, ΔΔ*C*_t_ values were calculated for the two T4 qPCR targets for each replicate at each time point. Fold-change values were then calculated for each replicate relative to the mean ΔΔ*C*_t_ value for cells carrying pWEB-TNC infected with T4 phage at the earliest time point post-infection for a given experiment. The mean fold change of three biological replicates was plotted for each time point post-infection.

### Next-generation sequencing of phage DNA in T4-infected *E. coli* cells

Overnight cultures of *E. coli* EC100 cells carrying pWEB-TNC or pBrig1 were diluted 1:50 in 10 ml of LB medium supplemented with 12.5 μg ml^−1^ chloramphenicol. After 1 h 15 min of growth, OD_600_ was measured, and cultures were normalized to OD_600_ = 0.3. Cultures were then infected at MOI 5 with T4 or T4 escaper1 for 8 min at 37 °C with shaking, prior to centrifugation at 15,000*g* for 5 min at 4 °C and subsequent freezing of cell pellets at −80 °C. All cell pellets were stored at −80 °C at least overnight, until ready for genomic DNA purification using the Promega Wizard Genomic DNA Purification Kit (Promega, A1125) following the protocol for Gram-negative bacteria. Purified genomic DNA was sheared using a pre-split snap-cap 6×16 mm Covaris microTUBE (Covaris, 520045) in a Covaris S220 focused-ultrasonicator and prepared for next-generation sequencing using the Illumina TruSeq Nano DNA LT kit (Illumina, 20015964). Paired-end 2 × 75 bp sequencing was conducted using the 150-cycle MiSeq Reagent Kit v3 (Illumina, MS-102-3001) on the Illumina MiSeq platform. Illumina paired-end sequencing reads were aligned to phage genomes using a custom Python script, where the recorded number of phage-derived sequencing reads at a specific base pair position within the phage genome was normalized to the total sequencing reads for each sample.

### Phage DNA extraction

Phage genomic DNA was extracted from capsids using a previously described protocol^55^. In brief, three tubes of 450 μl of a phage stock were first treated with DNase I (Invitrogen, 18068015) and RNase A (Promega, A7973) in DNase I buffer (20 mM Tris-HCl, pH 8, 2 mM MgCl_2_), the reaction stopped with EDTA (Invitrogen, AM9260G), then capsids digested with Proteinase K (NEB, P8107S), and finally phage genomic DNA extracted using the DNeasy Blood & Tissue kit (QIAGEN, 69504). DNA was quantified using the Qubit dsDNA HS Assay Kit and assessed for quality using a nanodrop spectrophotometer.

### T4 and T4 escaper1 genome sequencing and assembly

Phage genomic DNA was sequenced using the Nextera XT DNA Library Preparation Kit (Illumina, FC-131-1024). Paired-end 2 × 75 bp sequencing was conducted using the 150-cycle MiSeq Reagent Kit v3 (Illumina, MS-102-3001) on the Illumina MiSeq platform. Reads were quality-trimmed using Sickle (https://github.com/najoshi/sickle) and assembled into contigs using ABySS (https://github.com/bcgsc/abyss). Finally, contigs were mapped to a reference phage T4 genome (GenBank: AF158101.6) using Medusa (http://combo.dbe.unifi.it/medusa). Automated genome annotation was performed using SnapGene and a reference phage T4 genome from NCBI (GenBank: AF158101.6). Alignment of the T4 and T4 escaper1 genomes to the reference T4 genome revealed differential mutations between the two assembled phage genomes.

### Sanger sequencing of bacteriophage escapers

T4 or T6 phage plaques on lawns of *E. coli* EC100 cells carrying pBrig1 were isolated and resuspended in 20 μl of LB medium. Serial dilutions were prepared from the resuspended phage and spotted on a fresh LB top agar lawn of *E. coli* EC100 carrying pBrig1 to maintain selection of the escaper phage. The plate was incubated at 37 °C overnight. The next day a single phage plaque was picked from the top agar lawn using a P20 pipette set to 15 μl and resuspended in 20 μl of colony lysis buffer^48^. Resuspended phage mixtures were boiled at 98 °C for 15 min in a thermal cycler, and 1 μl of the boiled phage mixture was then used as template for PCR amplification using Phusion High-Fidelity DNA Polymerase (Thermo Scientific, F530L) with primers AA681/AA682 to amplify T4 *a-gt* and primers AA1115/AA1116 to amplify T6 *a-gt*. PCR products were submitted to Sanger sequencing by Genewiz/Azenta to identify mutations in *a-gt*. Wild-type T4 and T6 phage stocks were also PCR-amplified at *a-gt* loci and sent for Sanger sequencing to provide reference sequences for comparison. Snapgene was used to align Sanger sequencing products of the escaper phages to wild-type *a-gt* sequences to identify escape mutations.

### Brig1 structural predictions using AlphaFold2

The structure of the intact (261 amino acid) Brig1 protein was predicted using the colab implementation of AlphaFold2^17,18^ (https://colab.research.google.com/github/sokrypton/ColabFold/blob/main/AlphaFold2.ipynb) using default settings (except that the amber option was turned on to improve side chain rotamers). The highest ranked PDB structure produced by ColabFold (ptm = 0.86) was then visualized using PyMOL (The PyMOL Molecular Graphics System, Version 2.5.5, Schrödinger, LLC; www.pymol.org/pymol.html). Protein structure predictions of the Brig1 homologues from *Nocardioides zhouii* and *Nocardiodes anomalus* were performed in the same way. Cavities and pockets were visualized in PyMOL using default settings for surface calculation and the ‘cavities and pockets only’ option for display.

### Purification of Brig1

The *brig1* gene was recloned into the Nde1 and XhoI sites of pET21a using PCR primers that destroyed the XhoI site and added a His_6_ tag immediately after the native C-terminal glycine of Brig1. The insert was verified by DNA sequencing. *E. coli* strain Rosetta(DE3)pLysS was used for protein expression. Cells were grown in LB medium with 100 μg ml^−1^ ampicillin at 37 °C. 0.5 mM IPTG was added to induce protein expression when OD_600_ ~ 0.7, followed by further growth at 37 °C for 2 h. Cell pellets were resuspended in Ni column buffer A (50 mM phosphate, 1 M NaCl, 5% glycerol, 1 mM DTT, pH 7.5) with complete mini protease inhibitor cocktail (Roche), one tablet per litre culture. After adding lysozyme to a final concentration of 200 mg ml^−1^, the mixture was sonicated 3 times for 1 min each, then centrifuged at 20,000 rpm in an SS-34 rotor for 1 h. The supernatant was filtered and loaded onto a Ni column (Cytiva, HisTrap HP, 17-5248-02), and eluted with a 30-minute gradient of 0 to 100% buffer B (Ni buffer A plus 500 mM imidazole, pH 7.5). Brig1-containing fractions were pooled and diluted with heparin column buffer A (25 mM MES, 0.5 mM EDTA, 5% glycerol, 1 mM DTT, pH 6), loaded on a heparin column (Cytiva, HiTrap Heparin HP, 17-0406-01) and eluted with a gradient from 10% to 70% heparin buffer B (heparin column buffer A + 2 M NaCl, pH 6) over 90 min. The purest fractions were pooled and concentrated, then dialysed into storage buffer (20 mM Tris, 0.5 mM EDTA, 200 mM NaCl, 20% glycerol, 2 mM DTT, pH 8) and flash-frozen in small aliquots.

### Purification of T4 α-glucosyltransferase

A pET15b derivative encoding N-terminally His_6_ tagged phage T4 α-GT was used for protein expression. Rosetta(DE3)pLysS cells harbouring this plasmid were grown and induced as for Brig1, but after induction grown at 20 °C overnight rather than for 2 h at 37 °C. The same purification protocol as for Brig1 was followed except for a change in the pH of the heparin column buffers (A = 25 mM HEPES, 0.5 mM EDTA, 5% glycerol, 1 mM DTT, pH 7 and B = A + 2 M NaCl, pH 7). The purest fractions were pooled and concentrated, then dialysed into storage buffer (20 mM Tris, 0.5 mM EDTA, 200 mM NaCl, 20% glycerol, 2 mM DTT, pH 8) and flash-frozen in small aliquots.

### Purification of Brig1(Y121A/E147A) mutant

The Brig1(Y121A/E147A) mutant protein was purified according to a modified protocol. For consistency, wild-type Brig1 was purified according to this same protocol, side-by-side, and this batch of purified Brig1 protein was used only in experiments where Brig1(Y121A/E147A) was used. Both Brig1 and Brig1(Y121A/E147A) were cloned into a pET21a vector, with a His_6_ tag immediately after the native C-terminal glycine of Brig1. *E. coli* strain BL21(DE3) was used for protein expression. Cells were grown in LB medium with 100 μg ml^−1^ ampicillin at 37 °C overnight. The next day, a 1:100 dilution of the overnight was grown in 1 l of LB medium with 100 μg ml^−1^ ampicillin at 37 °C for 3-4 h. 0.5 mM IPTG was added to induce protein expression when OD_600_ ~ 0.7, followed by overnight growth (~16 h) at 18 °C. Cells were pelleted at 4500 rpm at 4 °C (Eppendorf Centrifuge 5810 R) for 15 min. Cell pellets were resuspended in 20 ml of lysis buffer (50 mM HEPES, pH 7.7, 150 mM NaCl, 10% glycerol, 1 mM TCEP, 30 mM imidazole, 2 Roche mini protease inhibitor tabs EDTA free, 0.5 mg ml^−1^ lysozyme) and incubated on ice for 1 h with shaking. The resuspended pellets were then sonicated using a Qsonica Q500 sonicator (70% amplitude with 10 s on, 30 s off for 2.5 min). The sonicated samples were spun down at 12,000 rpm at 4 °C (Eppendorf Centrifuge 5810 R) for 30 min and the supernatant run through a gravity column loaded with 3 ml of HisPur Ni-NTA Resin (Thermo Scientific, 88222). Before passing supernatant, the column was equilibrated with equilibration buffer (50 mM HEPES, pH 7.7, 150 mM NaCl, 10% glycerol, 1 mM TCEP, 30 mM imidazole). Then, the ~20 ml of sonicated cell pellet supernatant was passed through the column. The column was washed twice with 25 ml wash buffer (50 mM HEPES, pH 7.7, 500 mM NaCl, 10% glycerol, 1 mM TCEP, 30 mM imidazole) and then eluted with 20 ml elution buffer (50 mM HEPES, pH 7.7, 150 mM NaCl, 10% glycerol, 1 mM TCEP, 300 mM imidazole). The eluted protein was concentrated to <500 μl using an Amicon Ultra-4 Centrifugal Filter, 10 kDa MWCO (Millipore, UFC801024), with multiple rounds of centrifugation at 4,300*g* for 10 min at 4 °C (Eppendorf Centrifuge 5810 R), carefully resuspending the mixture between rounds of centrifugation via pipette mixing. The concentrated eluant was run on an ÄKTA pure chromatography system (Cytiva) fitted with a Superdex 75 Increase 10/300 GL column (Cytiva, 29148721) using storage buffer (50 mM HEPES, pH 7.7, 150 mM NaCl, 10% glycerol, 1 mM TCEP). Two peaks, corresponding to fractions 17–20 and 22–27, were collected and separately pooled. Pooled fractions were concentrated to <500 μl using an Amicon Ultra-0.5 Centrifugal Filter, 10 kDa MWCO (Millipore, UFC501096), with multiple rounds of centrifugation at 13,000*g* for 5 min in a tabletop microcentrifuge at 4 °C, carefully resuspending the mixture between rounds of centrifugation via pipette mixing. For both Brig1 and Brig1(Y121A/E147A), the second peak (fractions 22–26 for Brig1 and fractions 23–27 for the mutant) was determined to be free of nucleic acid contamination via nanodrop and found to contain pure protein (~29 kDa) by a Coomassie gel. Concentrated protein was flash-frozen in small aliquots and stored at −80 °C for future use.

### Annealing of ssDNA oligonucleotides

To generate dsDNA substrates for MfeI digestion and for DNA glycosylase assays, complementary ssDNA oligonucleotides were annealed. In brief, 1:1 molar ratios of top and bottom strand complementary ssDNA oligonucleotides (25–50 μM each) were mixed in a 60 μl reaction containing NaCl to a final concentration of 100 mM. The reaction was heated at 80 °C for 20 min in a water bath or thermal cycler and then allowed to cool very slowly to room temperature. Annealed oligonucleotides were purified using an oligonucleotide cleanup kit (Zymo Research, Oligo Clean & Concentrator Kit, D4061) according to the manufacturer’s instructions.

### Generation of glucosylated ssDNA and dsDNA oligonucleotides

We tested the activity of α-GT on both single- and double-stranded DNA as previous studies only tested dsDNA substrates^56^. The ssDNA substrates were hmdC_18, hmdC_60_MfeI and hmdC_60_MfeI_Bot, which are 18mer and 60mer oligonucleotides, each containing a single hmC residue (Supplementary Data File 2). The dsDNA substrate was hmdC_60_MfeI annealed to Bot_MfeI_60 (Supplementary Data File 2). Substrate DNAs (100 μM for ssDNA and 50 μM for dsDNA) were mixed at a 1:1 molar ratio with α-GT in 1× NEBuffer 4 (50 mM potassium acetate, 20 mM Tris-acetate, 10 mM magnesium acetate, 1 mM DTT, pH 7.9) supplemented with 2 mM UDP-glucose (NEB, supplied with NEB T4 β-GT). All samples were incubated at 37 °C overnight, then purified with an oligonucleotide cleanup kit (Zymo Research, Oligo Clean & Concentrator Kit, D4061) according to the manufacturer’s instructions. A subset of the ss- and dsDNA substrates were also treated with β-GT (NEB, M0357S) following the supplier’s instructions and purified as described above.

Modification by α-GT (or β-GT) was monitored by digestion with MfeI-HF (NEB, R3589S), which is blocked by the presence of glucosylated hmC but not by hmC (the modified C in hmdC_60_MfeI and in hmdC_60_MfeI_Bot is within an MfeI site, Supplementary Data File 2). Before digestion, single-stranded α-GT- or β-GT-treated hmdC_60_MfeI oligonucleotides were annealed to Bot_MfeI_60 or to untreated or α-GT-treated hmdC_60_MfeI_Bot. Approximately 1.5 μg (Extended Data Fig. 3e) or 500 ng (Extended Data Fig. 5c) of each sample was digested with MfeI-HF (NEB, R3589S) for 1 h, then electrophoresed on a 10% TBE gel (Invitrogen, EC6275BOX, 10-well or EC62755BOX, 15-well) at 140 V for 35 min. Gels were stained with 2 μg ml^−1^ ethidium bromide for 20 min, extensively rinsed with distilled water (3× for 10 min each), and then scanned using a ChemiDoc MP imager (Bio-Rad) set to UV trans illumination and the machine’s 605/50 filter to detect ethidium bromide (Extended Data Fig. 3e) or using the Amersham ImageQuant 800 set to UV fluorescence (Extended Data Fig. 5c).

### DNA glycosylase assays with ssDNA oligonucleotides

#### Detection of the abasic site with an aldehyde-reactive probe

We used an aldehyde-reactive fluorescent probe, AZDye 488 Hydroxylamine, (fluoroprobes.com) to detect removal of a base from the phosphodiester backbone in the absence of DNA cleavage. The dye was dissolved in distilled water to form a 10 μg μl^−1^ stock solution.

DNA glycosylase reactions were carried out in a reaction buffer containing 45 mM HEPES, pH 7.5, 0.4 mM EDTA, 2% glycerol, 1 mM DTT and 50 mM KCl, in a total reaction volume of 50 μl. The final DNA concentrations were 2 μM. Brig1 was added to single-stranded α-GT- and β-GT-treated hmdC_60_MfeI to a final concentration of 35 μM, while 2 μl (10 units; 5 units per μl) of SMUG1 (NEB, M0336S) was added to dU_60 as a positive control. Reactions were incubated overnight at 37 °C, after which 2 μl AZDye 488 dye was added, followed by incubation at 37 °C for 30 min. 1/10 volume of 10% SDS was then added and incubated for another 30 min and purified by phenol/chloroform extraction. Samples were then treated with an oligonucleotide cleanup kit (Zymo Research, Oligo Clean & Concentrator Kit, D4061) according to the manufacturer’s instructions, eluted with 15 μl nuclease-free water, mixed with loading dye and electrophoresed for 45 min at 180 V on a 10% TBE gel (Invitrogen, EC62755BOX). The gel was stained with 2 μg ml^−1^ ethidium bromide for 20 min, extensively rinsed with distilled water (3× for 10 min each), then scanned using a ChemiDoc MP imager (Bio-Rad) set to UV trans illumination and the machine’s 605/50 filter to detect ethidium bromide and then using blue epi illumination with the 530/28 filter for the AZDye 488 fluorescent probe.

#### Detection by NaOH- or endonuclease IV-mediated cleavage of the abasic site

DNA glycosylase reactions were carried out in a reaction buffer containing 45 mM HEPES, pH 7.5, 0.4 mM EDTA, 2% glycerol, 1 mM DTT and 50 mM KCl, in a total reaction volume of 50 μl. The final ssDNA or dsDNA concentrations were 1 μM. Brig1 or Brig1(Y121A/E147A) was added to a final concentration of 1 μM (unless stated otherwise—for example, range of 50–1,600 nM in Extended Data Fig. 7b), while 1 μl (5 units) of SMUG1 (NEB, M0336S) was added as a positive control. Reactions were incubated at 37 °C overnight, unless stated otherwise (for example, 30 min in Extended Data Fig. 7b). Following enzymatic incubation, one set of samples was directly processed with an oligonucleotide cleanup kit (Zymo Research, Oligo Clean & Concentrator Kit, D4061) according to the manufacturer’s instructions. A second matched set of samples was treated with NaOH before cleanup: 25 μl of 0.5 M NaOH was added to each 50 μl sample and then heated at 90 °C for 30 min before purification with the oligonucleotide cleanup kit (Zymo Research, Oligo Clean & Concentrator Kit, D4061) according to the manufacturer’s instructions. All samples were eluted from the cleanup columns in 15 μl nuclease-free water. Five microlitres of each was mixed with loading dye and loaded onto a 10% TBE gel (Invitrogen, EC62755BOX) and electrophoresed at 140 V for 35 min. Gels were stained with 2 μg ml^−1^ ethidium bromide for 20 min, extensively rinsed with distilled water (3× for 10 min each), and then scanned using a ChemiDoc MP imager (Bio-Rad) set to UV trans illumination and the machine’s 605/50 filter to detect ethidium bromide (Extended Data Fig. 3h) or using the Amersham ImageQuant 800 set to UV fluorescence (all other relevant figures). For Urea-PAGE gels, eluted samples were first denatured by mixing 5 μl of purified sample with 5 μl of 2× TBE-Urea Sample Buffer (Invitrogen, LC6876) and then heated at 70 °C for 3 min. Denatured samples were loaded onto a 6% TBE-Urea gel (Invitrogen, EC68655BOX) and electrophoresed at 140 V for 35 min. Gels were soaked in ethidium bromide and rinsed with distilled water as described above, before imaging with the Amersham ImageQuant 800 set to UV fluorescence. For all gels, DNA ladders were made by mixing 20-, 40- and 60-bp ssDNA or dsDNA oligonucleotides (Supplementary Data File 2) and loading them onto their corresponding gels at ~100 ng each oligonucleotide per load.

For abasic site detection by NEB endonuclease IV (Endo IV), DNA glycosylase reactions were set up as described above and incubated with Brig1 overnight. Three matched sets of reactions were set up. After overnight incubation, one matched set of samples was treated with NaOH as described above and purified using the Zymo Research Oligo Clean & Concentrator Kit (D4061) according to the manufacturer’s instructions. The remaining two matched sets of samples were processed directly using the oligonucleotide cleanup kit. The purified samples were then incubated at 37 °C for 4 h in a 50 μl reaction with 1× NEBuffer 3 (100 mM NaCl, 50 mM Tris-HCl, 10 mM MgCl_2_, 1 mM DTT, pH 7.9), with or without 50 units of NEB Endo IV (5 μl; 10 units per μl; M0304S). After 4 h, reactions were purified using the Zymo Research Oligo Clean & Concentrator Kit according to the manufacturer’s instructions. All the purified samples were then loaded onto a 10% TBE gel (Invitrogen, EC62755BOX), electrophoresed at 140 V for 35 min, stained with ethidium bromide as described above and imaged with the Amersham ImageQuant 800 set to UV fluorescence.

### High-resolution mass spectrometry of SMUG1- and Brig1-treated ssDNA oligonucleotides

DNA glycosylase reactions were carried out in a reaction buffer containing 45 mM HEPES, pH 7.5, 0.4 mM EDTA, 2% glycerol, 1 mM DTT and 50 mM KCl, in a total reaction volume of 50 μl. Reactions were performed with 18mer ssDNA oligonucleotides: dU_18, hmdC_18 and α-GT-treated hmdC_18 (Supplementary Data File 2). The final ssDNA concentration in each reaction was 2 μM. Brig1 was added to a final concentration of 2 μM, while 2 μl (10 units) of SMUG1 (NEB, M0336S) was added as a positive control. A no-enzyme reaction was used as a negative control. 2 ×50 μl reactions were set up for each reaction condition with the dU_18 oligonucleotide, while 8 ×50 μl reactions were set up for each reaction condition with hmdC_18 and α-GT-treated hmdC_18. Reactions were incubated overnight at 37 °C. After overnight incubation, all matched samples were pooled and processed with an oligonucleotide cleanup kit (Zymo Research, Oligo Clean & Concentrator Kit, D4061) according to the manufacturer’s instructions.

For mass spectrometry, purified oligonucleotide samples were dried using vacuum centrifugation and dissolved in 50/50 water/acetonitrile with 0.001% triethylammonium bicarbonate. The pH of the solution was found to be comparable to that of deionized water. The samples were introduced to the mass spectrometer by manual injection using a Hamilton syringe applying pressure by hand at approximately 10 μl min^−1^. Samples were analysed using an orbitrap Ascend tribrid mass spectrometer (Thermo Scientific) operating in negative mode. Spectra were recorded in the mass range 600–1,300 *m/z* at 120,000 resolution. A blank injection was introduced after each sample to eliminate carryover.

Raw data was inspected using the Xcalibur Quality Browser (Thermo Scientific) and spectra were summed as necessary to provide representative spectra with a sufficient signal-to-noise ratio (S/N). Spectra were further processed using UniDec deconvolution software^57^ with the following parameters: sampling resolution and peak FWHM were both set to 0.1, adduct mass was defined as −1.007276 Da, and charge states were defined 4–12 based on observations from the raw data. The *m/z* range was adjusted to fit the data and to exclude singly charged noise. Apart from the mass of the oligonucleotides, additional masses from metal adducts were also observed.

### DNA glycosylase assays with phage and cosmid DNA

All reactions were performed in 50 μl reaction volumes in a reaction buffer containing 45 mM HEPES, pH 7.5, 0.4 mM EDTA, 2% glycerol, 1 mM DTT and 50 mM KCl. Assays were performed by incubating 50–500 ng of extracted phage genomic DNA from capsids or miniprepped pWEB-TNC cosmid DNA with varying concentrations (2–800 nM) of purified Brig1 or Brig1(Y121A/E147A) or with 10 units of NEB SMUG1 (NEB, M0336S) as a negative control. Reactions were incubated in a thermal cycler at 37 °C for 30 min (or at 37 °C for 30 min plus an additional 20 min at 65 °C in Extended Data Fig. 6c, to cleave DNA at abasic sites and denature the glycosylase prior to gel electrophoresis). Reactions were then mixed with 10 μl of purple 6× loading dye with no SDS (NEB, B7025S) and the entire reaction volume was loaded onto a 1% agarose gel containing ethidium bromide. Unless stated otherwise, the gel was run for 70 min at 85 V at room temperature and then imaged using a UV gel imager (Amersham ImageQuant 800 set to UV fluorescence). Where SDS was used for protein denaturation (Extended Data Fig. 6d), all steps were carried out as described above except, before gel loading, a purple 6× gel loading dye containing a final 1× concentration of 0.08% SDS (NEB, B7024S) was used instead of loading dye without SDS.

For the gel in Extended Data Fig. 6a,b, samples were loaded onto the 1% agarose gel in a cold room at 4 °C and run at 40 V for 3 h and then imaged using a UV gel imager (Amersham ImageQuant 800 set to UV fluorescence). The same gel was then allowed to equilibrate to room temperature for 30 min and then run longer by electrophoresis, this time under high voltage and at room temperature (in a room temperature gel box), first at 85 V for 25 min, then at 150 V for 8 min and finally at 200 V for another 8 min before final imaging using the Amersham ImageQuant 800 set to UV fluorescence.

### Brig1 multiple sequence alignment and phylogenetic tree construction

Brig1 homologues were obtained using the NCBI PSI-BLAST protein homology search. Homologues were then subjected to a multiple sequence alignment using MUSCLE v5 with 16 maximum iterations via the Geneious Prime software. A tree was built with the alignment output file via IQ-TREE 1.6.12^58^ using the LG4M model with 1,000 bootstrap alignments. The online tool ITOL^59^ was used for visualization of the resulting tree.

### Brig1 gene neighbourhood analysis

Gene neighbourhoods of the *brig1* homologues from above (10 genes upstream and 10 genes downstream of each homologue) were constructed using a custom Python script. In brief, the script parses a blastp result XML file for accession numbers of each of the hits. For each hit accession, the script obtains the corresponding nucleotide accession from which the protein accession is derived. Finally, all annotated features within the nucleotide accession that are labelled as ‘CDS’ or ‘tRNA’ are built into a list, including their position within the nucleotide entry and their feature name. From this list, neighbours of the initial protein hit (10 genes upstream and 10 genes downstream) are extracted and built into a TSV file for subsequent analysis.

### Statistical analysis

Statistical analyses were performed using GraphPad Prism version 10.1.0. Error bars and number of replicates for each phage experiment are defined in the figure legends. Statistical significance in Extended Data Fig. 2i was determined using a two-tailed Student’s *t*-test (an unpaired parametric test assuming Gaussian distribution and that both populations have the same standard deviation).

### Reporting summary

Further information on research design is available in the Nature Portfolio Reporting Summary linked to this article.

## Online content

Any methods, additional references, Nature Portfolio reporting summaries, source data, extended data, supplementary information, acknowledgements, peer review information; details of author contributions and competing interests; and statements of data and code availability are available at 10.1038/s41586-024-07329-9.

### Supplementary information

Supplementary Information This file contains Supplementary Figs. 1 and 2, discussion, sequences and Table 1.

Reporting Summary

Supplementary Data File 1 Bioinformatic analysis of Brig1 homologues and eDNA fragments isolated in this study.

Supplementary Data File 2 Strains, plasmids, phages and oligonucleotides used in this study.

### Source data

Source Data Fig. 1

Source Data Fig. 2

Source Data Extended Data Fig. 2

Source Data Extended Data Fig. 4

Source Data Extended Data Fig. 9

## Data Availability

Lists of strains, plasmids, bacteriophages, oligonucleotides and CRISPR spacers used in this study are available in Supplementary Data File 2. The raw FASTQ files for the next-generation sequencing experiments can be found at the NCBI Sequence Read Archive (SRA) under BioProject PRJNA1045662. The DNA sequence of the approximately 34.5 kb metagenomic DNA fragment harbouring the *brig1* gene is deposited in NCBI GenBank under accession code OR880862. The NCBI protein accession codes of the *brig1* homologues from *N. zhouii* and *N. anomalus* reported in this study are WP_129427366.1 and WP_165228961.1, respectively. Source data are provided with this paper.

## Code availability

Custom Python scripts used for data analysis are deposited at https://github.com/Marraffini-Lab/Hossain_etal_2024 and also available at Zenodo (10.5281/zenodo.10815574 (ref. ^60^)).
