# Supplementary material for: DNA glycosylases provide antiviral defence in prokaryotes
Source: Nature. 2024 Apr 17;629(8011):410–6. doi: 10.1038/s41586-024-07329-9 (PMC11078745; doi:10.1038/s41586-024-07329-9)
Supplement: Supplementary file 1 — This file contains Supplementary Figs. 1 and 2, discussion, sequences and Table 1. [file 41586_2024_7329_MOESM1_ESM.pdf]

nature portfolio

https://doi.org/10.1038/s41586-024-07329-9

**Supplementary information**

# DNA glycosylases provide antiviral defence in prokaryotes

In the format provided by the authors and unedited

**Supplementary Table 1 Mutations in the *a-gt* gene of T4 and T6 phages that escape Brig1 immunity.**

| escaper | Mutations in *a-gt* gene |
|---|---|
| T4-1 | 1 bp deletion of nt 713 (frameshift) |
| T4-2 | 1 bp insertion (A) after nt 294 (frameshift) |
| T4-3 | nt 1031 G>A mutation (Gly344->Asp344) |
| T4-4 | nt 793 C>G mutation (Pro265->Ala265) + 1 bp insertion (T) after nt 801 (frameshift) |
| T4-5 | 1 bp deletion of nt 294 (frameshift) |
| T4-6 | nt 1072 G>T mutation (Glu358->TAA STOP) |
| T4-7 | 1 bp deletion of nt 294 (frameshift) |
| T4-8 | 1 bp insertion (T) after nt 996 (frameshift) |
| T4-9 | nt 931 G>A mutation (Glu311->Lys311) |
| T4-10 | nt 245 C>T mutation (Ser82->Phe82) |
| T4-11 | 1 bp insertion (A) after nt 294 (frameshift) |
| T4-12 | 1 bp insertion (A) after nt 294 (frameshift) |
| T4-13 | 1 bp insertion (A) after nt 294 (frameshift) |
| T4-14 | 1 bp deletion of nt 182 (frameshift) |
| T4-15 | 1 bp insertion (A) after nt 294 (frameshift) |
| T4-16 | nt 926 A>G mutation (His309->Arg309) |
| T4-17 | nt 977 G>A mutation (Gly326->Asp326) |
| T4-18 | nt 419 A>G mutation (His140->Arg140) |
| T4-19 | 1 bp insertion (A) after nt 294 (frameshift) |
| T6-1 | nt 716 C>T mutation (Ala239->Val239) |
| T6-2 | 1 bp insertion (C) after nt 758 (frameshift) |

## Supplementary Discussion

*Restriction systems with similarities to Brig1*

Functionally, but not structurally, Brig1 is related to the restriction enzymes GmrSD and R.PabI, albeit in different ways. Like Brig1, the *E. coli* type IV restriction enzyme GmrSD targets glucosylated hmC-containing DNA in T-even phages[1]. Unlike Brig1, however, GmrSD targets both alpha- and beta-glucosyl-hmC as well as gentiobiosyl-hmC. GmrSD contains an HNH endonuclease motif that cleaves phosphodiester bonds to introduce double-strand DNA breaks[2]. Brig1 in contrast possesses a pyrimidine DNA glycosylase pocket with base excision activity on both ssDNA and dsDNA substrates. Interestingly, the T-even phages carry a GmrSD inhibitor, IPI*, which enables viral propagation in the presence of this restriction enzyme in *E. coli*[3]. In a similar vein, phages with glucosylated cytosines that encounter Brig1 in their native hosts may have evolved inhibitors of Brig1. In support of this prediction, *Bacillus* phages PBS1/PBS2 and $\Phi$29, which incorporate uracil in their genomes and are subject to restriction by host uracil DNA glycosylases involved in base excision repair, encode inhibitors of this enzyme, UGI[4,5] and p56[6,7], respectively. In contrast to Brig1 and GmrSD, the restriction enzyme R.PabI from the hyperthermophilic archaeon *Pyrococcus abyssi*, targets unmodified DNA and belongs to a family of type II restriction enzymes with a “half-pipe” structural fold[8]. R.PabI and related enzymes possess adenine DNA glycosylase activity that excises unmodified adenines within a specific DNA sequence: 5’-GTAC-3’[8,9]. The resultant abasic site ultimately leads to cleavage of the phosphodiester bond, either through heat-promoted beta-elimination[8], the presence of a secondary AP lyase activity in R.PabI[10,11], and/or by host AP endonucleases[10,11]. Therefore, while R.PabI is not a canonical restriction endonuclease, it provides immunity primarily through the generation of a site-specific double-strand DNA break like other type II restriction enzymes. In contrast, Brig1 appears to lack AP lyase activity during base excision and therefore provides anti-phage defense through a different molecular mechanism than both GmrSD and R.PabI.

*Evolutionary benefits of Brig1 immunity*

While Brig1 has the fundamental limitation that it only protects from infection by phages with alpha-glucosyl-hmC modifications, its distinct molecular mechanism could have several advantages over DNA-cleaving restriction endonucleases. For example, while most restriction enzymes recognize defined sequence motifs, resulting in a limited number of targets in the viral genome, the widespread distribution of alpha-glucosylated hmC residues enables Brig1 to cause extensive DNA damage, virtually across the entire genome of T-even phages. In addition, escape from restriction via methylation of target sites is a relatively common event that does not have major impacts on phage proliferation. In contrast, mutations in the genes encoding DNA-modifying enzymes that promote escape from Brig1 and other DNA glycosylases are in principle infrequent and can decrease viral fitness, as the mutant phages would become vulnerable to attack by nucleases that would otherwise be inhibited by DNA modifications. Another advantage of DNA glycosylases is that the abasic sites they generate are highly reactive[12] and can lead to a variety of deleterious DNA lesions beyond ssDNA and dsDNA cleavage, such

as DNA-DNA interstrand crosslinks and DNA-protein crosslinks that can stall the progression of both DNA and RNA polymerases. Finally, as mentioned above, Brig1's ability to target ssDNA substrates enables the enzyme to attack critical viral replication intermediates that cannot be accessed by restriction enzymes.

*The Brig1 operon*

In the Brig1 operon we isolated (Extended Data Fig. 1c, e), Brig1 is flanked by a protein with predicted ADP-ribosyl glycohydrolase activity. Interestingly, these enzymes remove ADP-ribose groups from proteins and DNA to counter ADP-ribosyltransferase (ART) toxins, commonly involved in bacteria-phage conflicts[13]. Phage T4 harbors three ARTs that ADP-ribosylate host proteins to facilitate the viral lytic cycle[14,15]. Although not required for immunity in our assays, it is possible that in other conditions, or after infection by phages expressing ARTs different than those of T4, the ADP-ribosyl glycohydrolase adjacent to Brig1 is required to de-toxify the host from ADP-ribosylation and facilitate recovery after infection has been cleared by Brig1 or another anti-phage system. Another possibility is that this gene participates in anti-phage defense in a manner similar to Brig1, i.e., by inhibiting the replication of phages carrying ADP-ribosylated genomes. We believe that the many different modified nucleobases present in phage genomes[16-22] could be the substrates of yet undiscovered DNA glycosylases involved in anti-phage defense, such as Brig1.

**References**


1 Bair, C. L. & Black, L. W. A type IV modification dependent restriction nuclease that targets glucosylated hydroxymethyl cytosine modified DNAs. *J. Mol. Biol.* **366**, 768-778, (2007).

2 Machnicka, M. A., Kaminska, K. H., Dunin-Horkawicz, S. & Bujnicki, J. M. Phylogenomics and sequence-structure-function relationships in the GmrSD family of Type IV restriction enzymes. *BMC Bioinformatics* **16**, 336, (2015).

3 Bair, C. L., Rifat, D. & Black, L. W. Exclusion of glucosyl-hydroxymethylcytosine DNA containing bacteriophages is overcome by the injected protein inhibitor IPI*. *J. Mol. Biol.* **366**, 779-789, (2007).

4 Wang, Z. & Mosbaugh, D. W. Uracil-DNA glycosylase inhibitor gene of bacteriophage PBS2 encodes a binding protein specific for uracil-DNA glycosylase. *J. Biol. Chem.* **264**, 1163-1171, (1989).

5 Savva, R. & Pearl, L. H. Cloning and expression of the uracil-DNA glycosylase inhibitor (UGI) from bacteriophage PBS-1 and crystallization of a uracil-DNA glycosylase-UGI complex. *Proteins* **22**, 287-289, (1995).

6 Serrano-Heras, G. *et al.* Protein p56 from the Bacillus subtilis phage phi29 inhibits DNA-binding ability of uracil-DNA glycosylase. *Nucleic Acids Res.* **35**, 5393-5401, (2007).

7 Serrano-Heras, G., Bravo, A. & Salas, M. Phage phi29 protein p56 prevents viral DNA replication impairment caused by uracil excision activity of uracil-DNA glycosylase. *Proc Natl Acad Sci U S A* **105**, 19044-19049, (2008).

8 Miyazono, K. *et al.* A sequence-specific DNA glycosylase mediates restriction-modification in Pyrococcus abyssi. *Nat Commun* **5**, 3178, (2014).
9 Ishikawa, K. *et al.* Discovery of a novel restriction endonuclease by genome comparison and application of a wheat-germ-based cell-free translation assay: PabI (5'-GTA/C) from the hyperthermophilic archaeon Pyrococcus abyssi. *Nucleic Acids Res.* **33**, e112, (2005).
10 Fukuyo, M. *et al.* Restriction-modification system with methyl-inhibited base excision and abasic-site cleavage activities. *Nucleic Acids Res.* **43**, 2841-2852, (2015).
11 Zhang, Y. *et al.* Restriction glycosylases: involvement of endonuclease activities in the restriction process. *Nucleic Acids Res.* **45**, 1392-1403, (2017).
12 Thompson, P. S. & Cortez, D. New insights into abasic site repair and tolerance. *DNA Repair (Amst)* **90**, 102866, (2020).
13 Aravind, L., Zhang, D., de Souza, R. F., Anand, S. & Iyer, L. M. The natural history of ADP-ribosyltransferases and the ADP-ribosylation system. *Curr. Top. Microbiol. Immunol.* **384**, 3-32, (2015).
14 Tiemann, B. *et al.* ModA and ModB, two ADP-ribosyltransferases encoded by bacteriophage T4: catalytic properties and mutation analysis. *J. Bacteriol.* **186**, 7262-7272, (2004).
15 Alawneh, A. M., Qi, D., Yonesaki, T. & Otsuka, Y. An ADP-ribosyltransferase Alt of bacteriophage T4 negatively regulates the Escherichia coli MazF toxin of a toxin-antitoxin module. *Mol. Microbiol.* **99**, 188-198, (2016).
16 Khudyakov, I. Y., Kirnos, M. D., Alexandrushkina, N. I. & Vanyushin, B. F. Cyanophage S-2L contains DNA with 2,6-diaminopurine substituted for adenine. *Virology* **88**, 8-18, (1978).
17 Swinton, D., Hattman, S., Benzinger, R., Buchanan-Wollaston, V. & Beringer, J. Replacement of the deoxycytidine residues in Rhizobium bacteriophage RL38JI DNA. *FEBS Lett.* **184**, 294-298, (1985).
18 Thomas, J. A., Orwenyo, J., Wang, L. X. & Black, L. W. The Odd "RB" Phage-Identification of Arabinosylation as a New Epigenetic Modification of DNA in T4-Like Phage RB69. *Viruses* **10**, (2018).
19 Hutinet, G. *et al.* 7-Deazaguanine modifications protect phage DNA from host restriction systems. *Nat Commun* **10**, 5442, (2019).
20 Kropinski, A. M. *et al.* The Sequence of Two Bacteriophages with Hypermodified Bases Reveals Novel Phage-Host Interactions. *Viruses* **10**, (2018).
21 Korn, A. M., Hillhouse, A. E., Sun, L. & Gill, J. J. Comparative Genomics of Three Novel Jumbo Bacteriophages Infecting Staphylococcus aureus. *J. Virol.* **95**, e0239120, (2021).
22 Weigele, P. & Raleigh, E. A. Biosynthesis and Function of Modified Bases in Bacteria and Their Viruses. *Chem Rev* **116**, 12655-12687, (2016).

## SUPPLEMENTARY FIGURE 1

pWEB-TNC/Phage T4

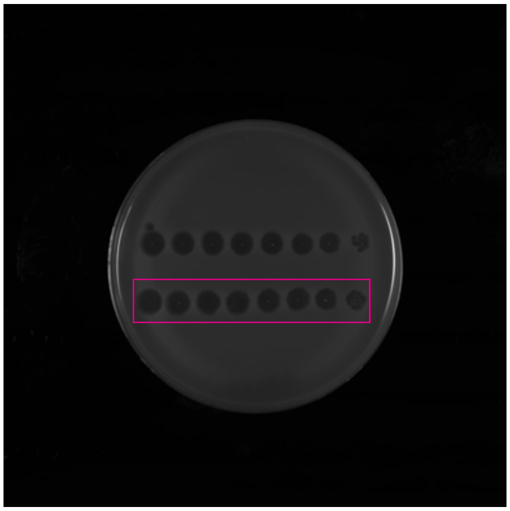

pFragmentD3/Phage T4

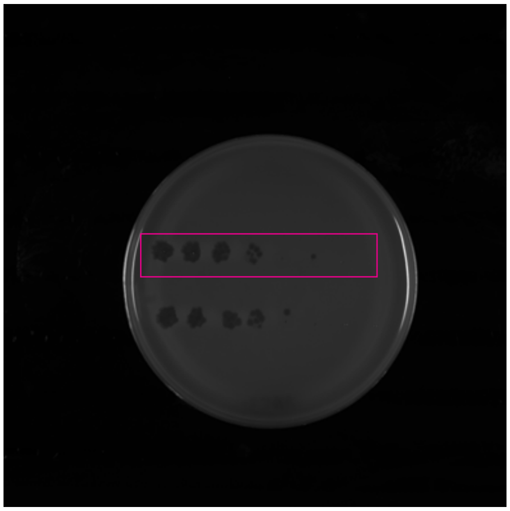

pFragmentD3-1/Phage T4

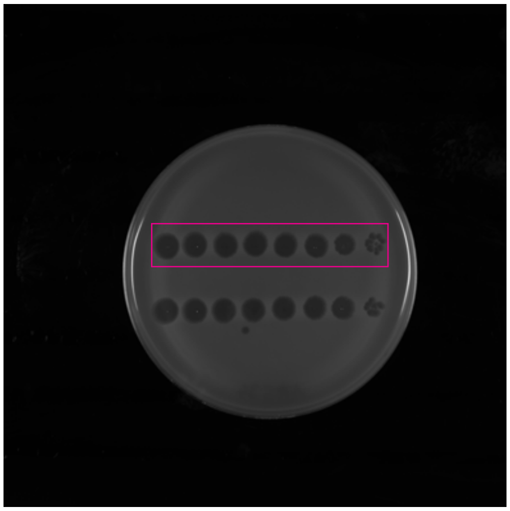

pFragmentD3-2/Phage T4

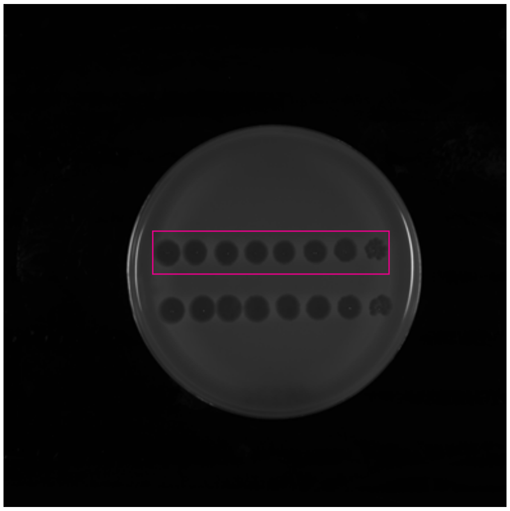

pFragmentD3-3/Phage T4

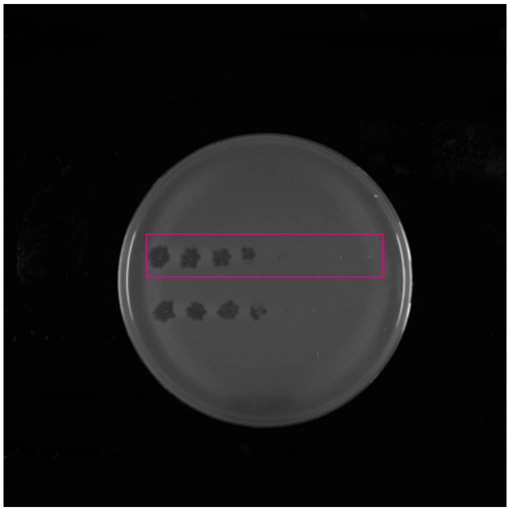

pFragmentD3-4/Phage T4

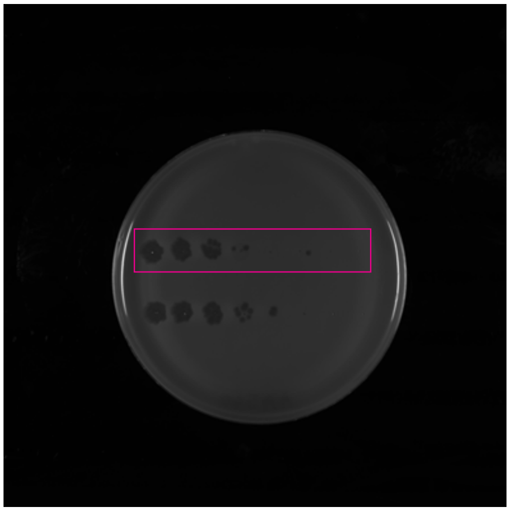

pFragmentD3-5/Phage T4

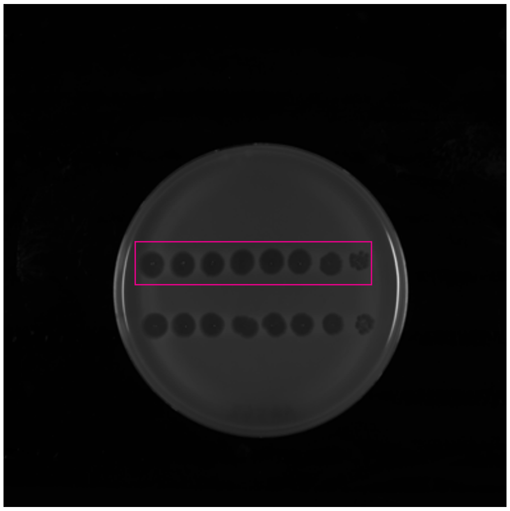

pFragmentD3-6/Phage T4

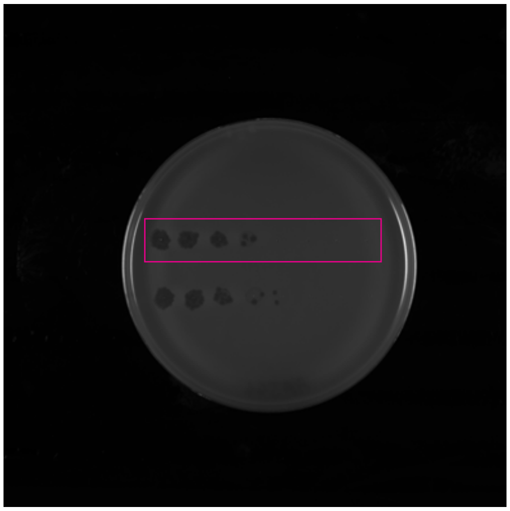

These are plate images for the representative images shown in Fig 1a. Images were cropped as shown and then auto contrasted on Adobe Photoshop.

**Fig. 1a**

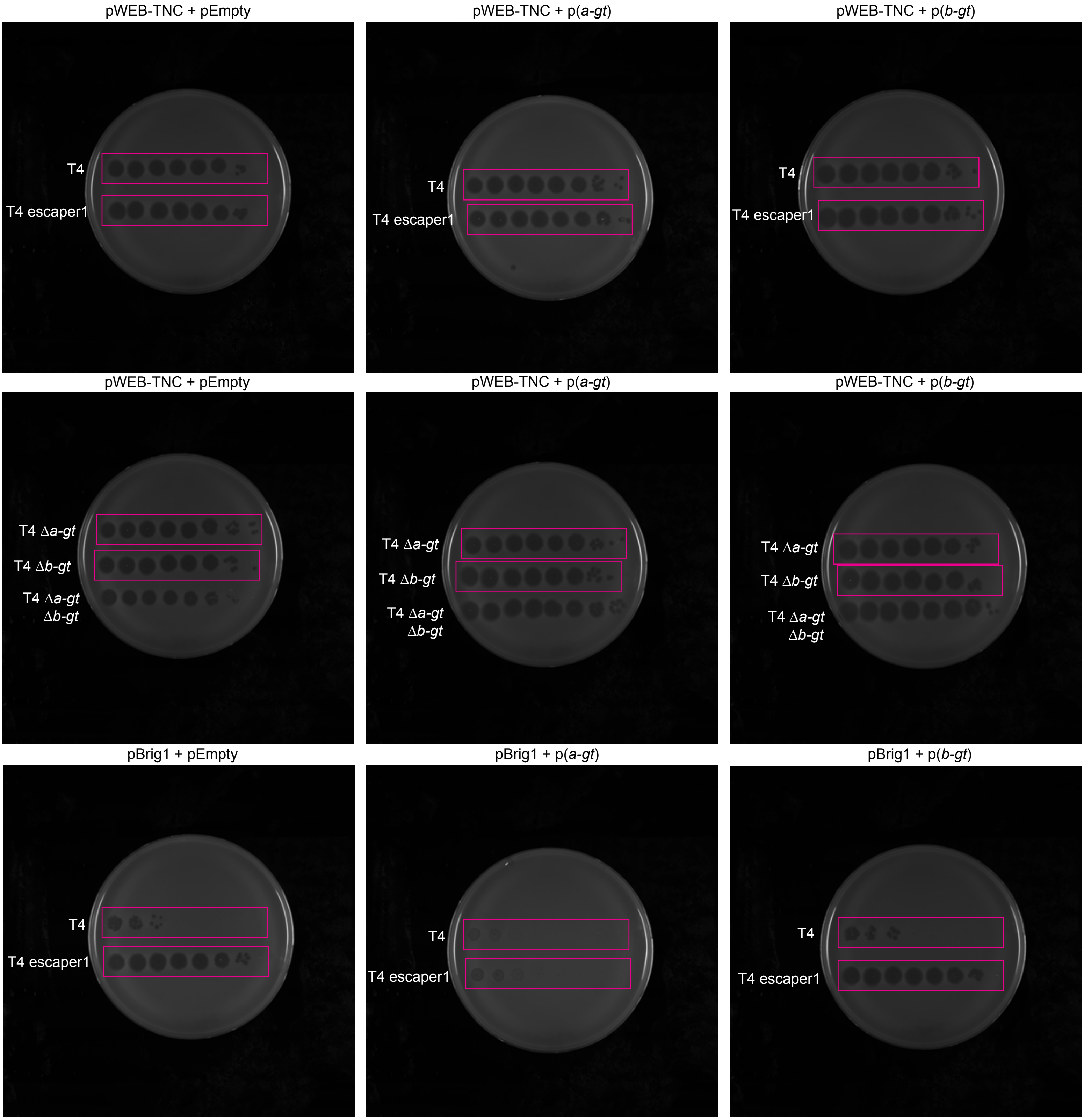


Continued on next page.
These are plate images for the representative images shown in Fig 2a. Images were cropped as shown and then auto contrasted on Adobe Photoshop; pBrig1 + p(*a-gt*) rows were further enhanced for brightness to enable better visibility.

**Fig. 2a**

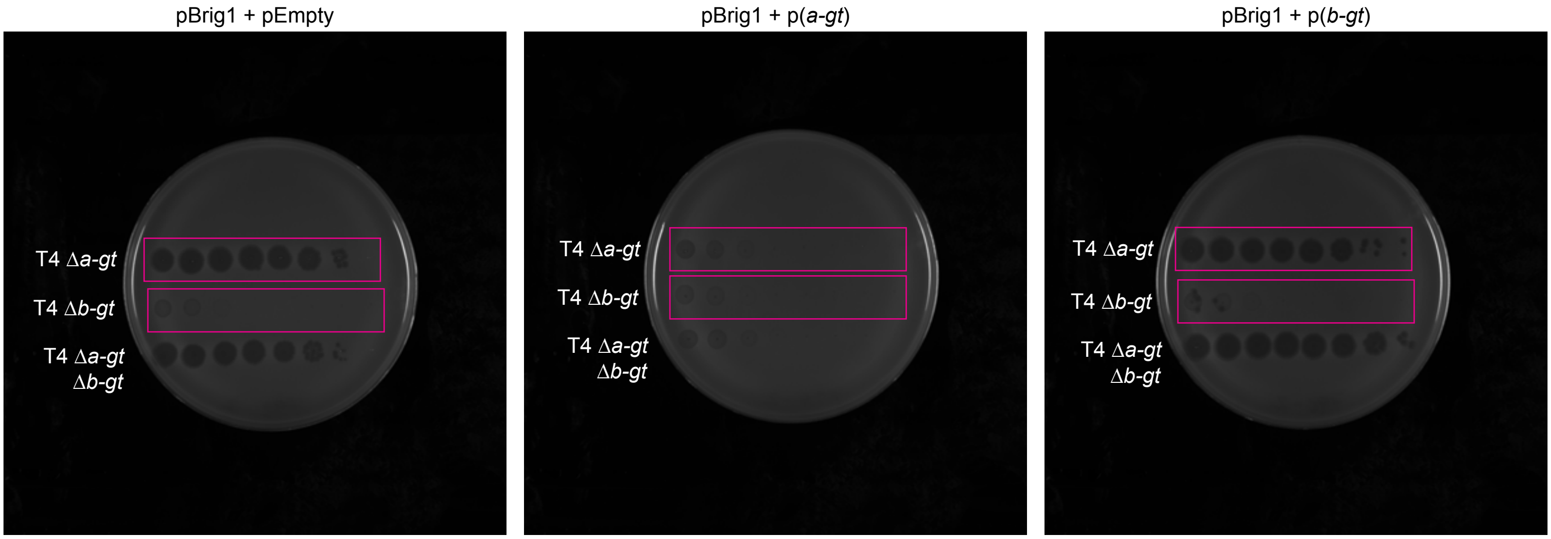


Continued from previous page.
These are plate images for the representative images shown in Fig 2a. Images were cropped as shown and then auto contrasted on Adobe Photoshop; pBrig1 + pEmpty row for phage T4 Δ*b-gt* and the pBrig1 + p(*a-gt*) rows were further enhanced for brightness to enable better visibility.

**Fig. 2a**

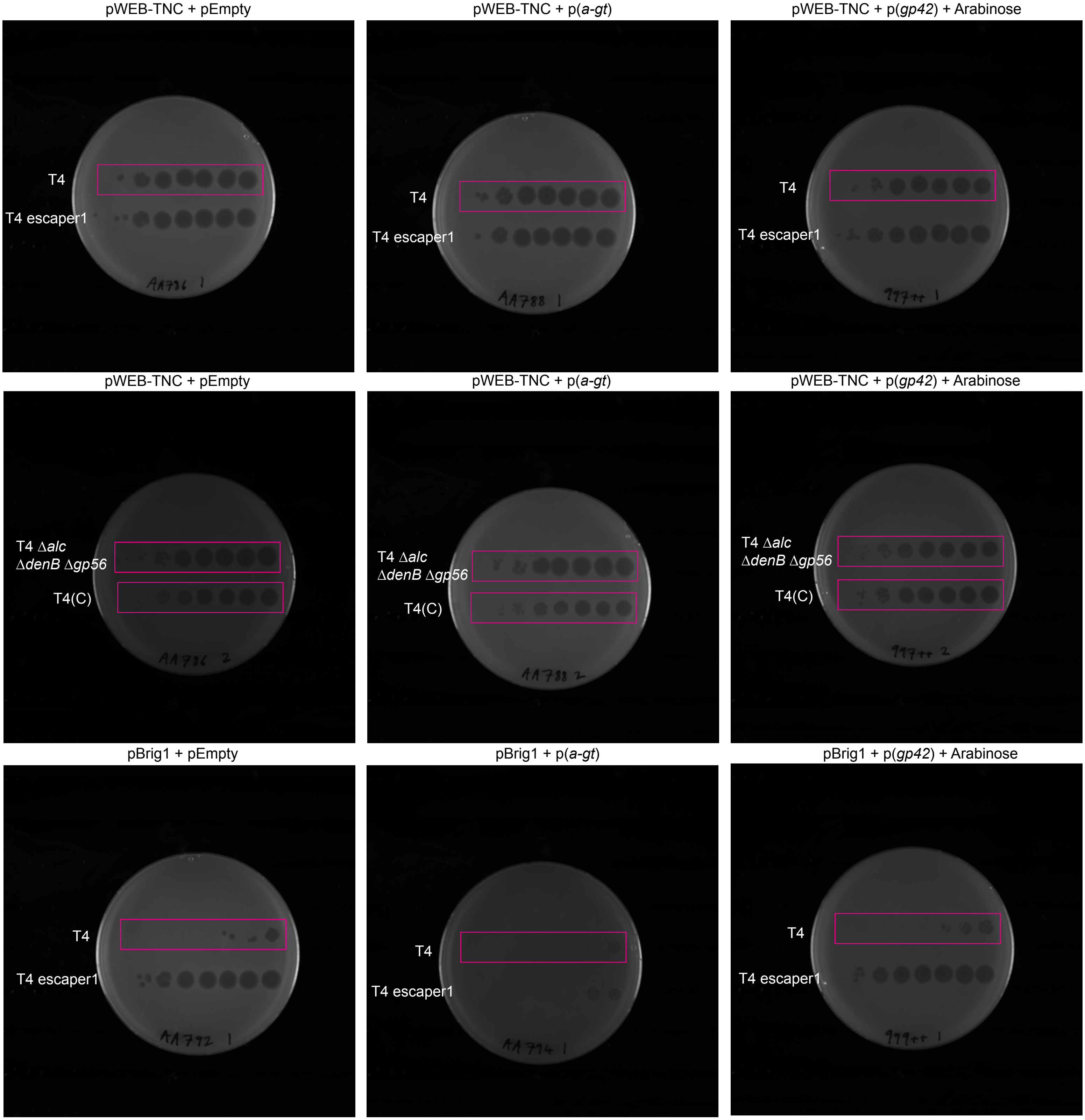


Continued on next page.

These are plate images for the representative images shown in Fig 2c. Images were cropped as shown and then auto contrasted on Adobe Photoshop; pBrig1 + p(*a-gt*) row for phage T4 was further enhanced for brightness to enable better visibility. Image reflected vertically in figure.

**Fig. 2c**

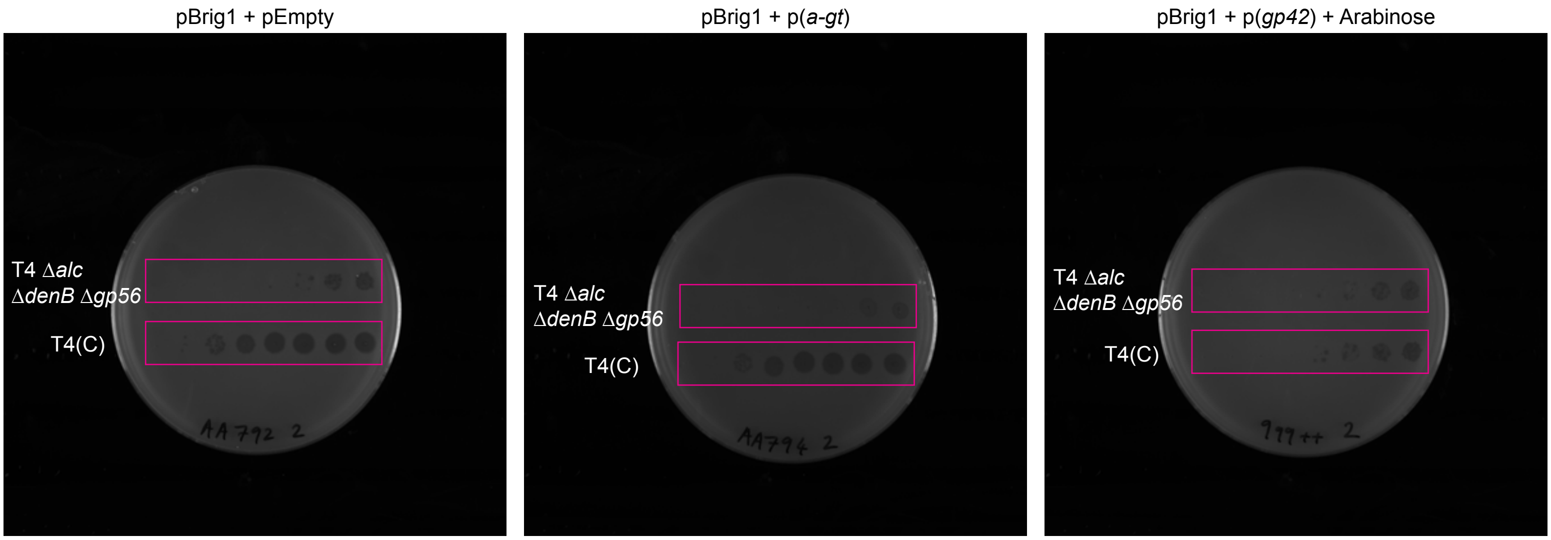


Continued from previous page.
These are plate images for the representative images shown in Fig 2c. Images were cropped as shown and then auto contrasted on Adobe Photoshop; pBrig1 + p(*a-gt*) row for phage T4 Δ*alc* Δ*denB* Δ*gp56* was further enhanced for brightness to enable better visibility. Image reflected vertically in figure.

**Fig. 2c**

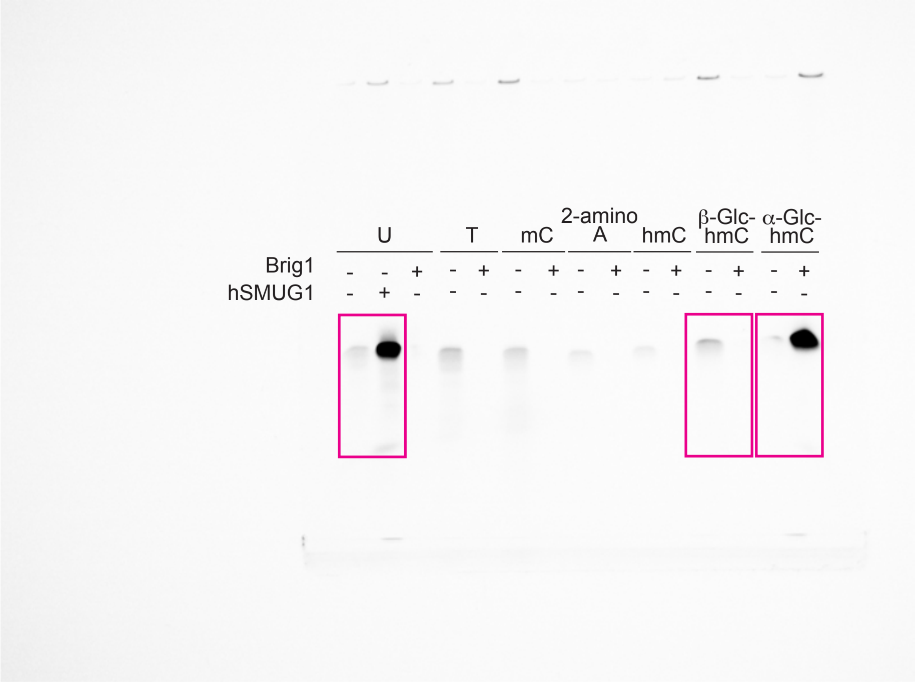


10% TBE gel
Imaged using ChemiDoc MP Imager (BioRad)
530/28 filter for AZDye 488 fluorescent probe, i.e. Alexa 488 aldehyde-reactive probe

Image cropped as shown

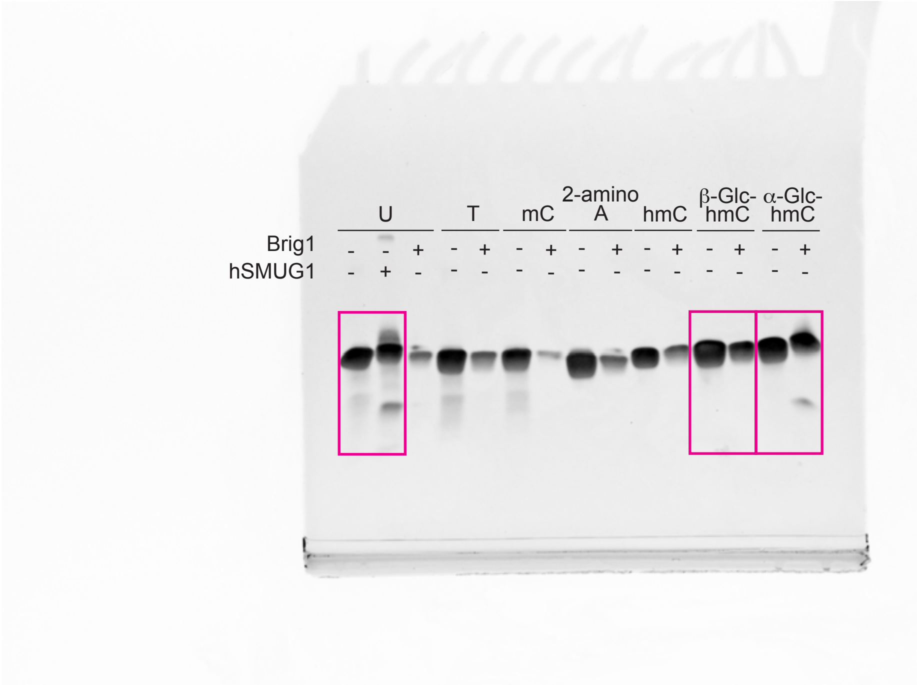


Same gel imaged using ChemiDoc MP Imager (BioRad)
605/50 filter for UV fluorescence (ethidium bromide)

Image cropped as shown

**Fig. 3b**

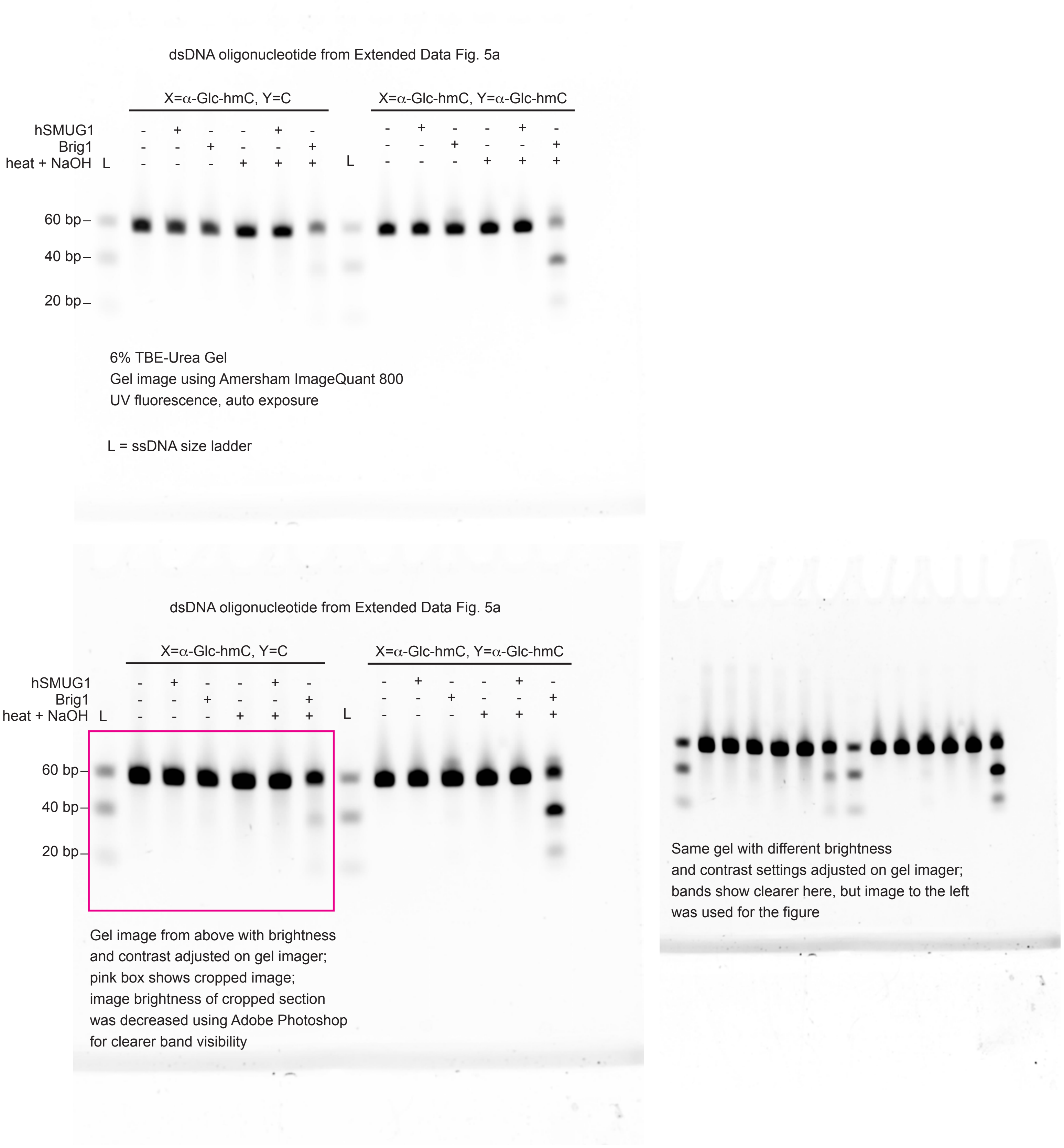
dsDNA oligonucleotide from Extended Data Fig. 5a
X=α-Glc-hmC, Y=C
X=α-Glc-hmC, Y=α-Glc-hmC
hSMUG1
Brig1
heat + NaOH
L
60 bp
40 bp
20 bp
6% TBE-Urea Gel
Gel image using Amersham ImageQuant 800
UV fluorescence, auto exposure
L = ssDNA size ladder
dsDNA oligonucleotide from Extended Data Fig. 5a
X=α-Glc-hmC, Y=C
X=α-Glc-hmC, Y=α-Glc-hmC
hSMUG1
Brig1
heat + NaOH
L
60 bp
40 bp
20 bp
Gel image from above with brightness and contrast adjusted on gel imager; pink box shows cropped image; image brightness of cropped section was decreased using Adobe Photoshop for clearer band visibility
Same gel with different brightness and contrast settings adjusted on gel imager; bands show clearer here, but image to the left was used for the figure

**Fig. 3c**

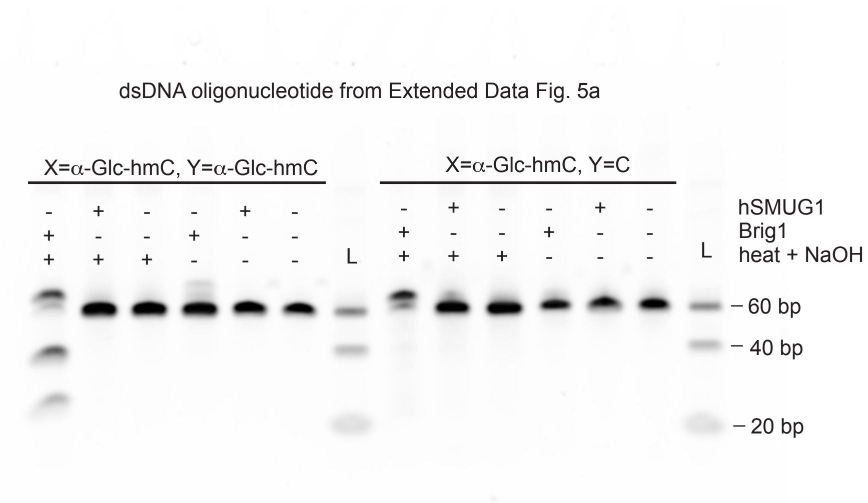


10% TBE Gel
Gel image using Amersham ImageQuant 800
UV fluorescence, auto exposure

L = dsDNA size ladder

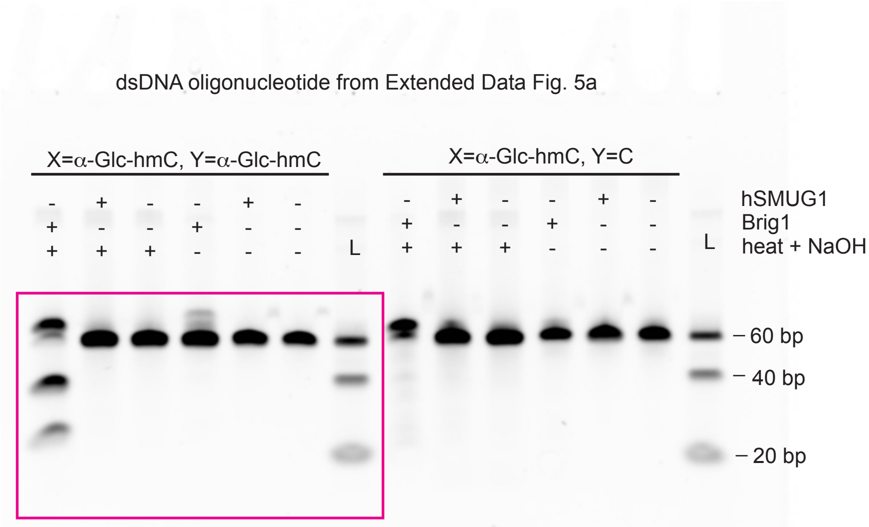


Gel image from above with brightness and contrast adjusted on gel imager; pink box shows cropped image; cropped image reflected vertically in final figure

**Fig. 3d**

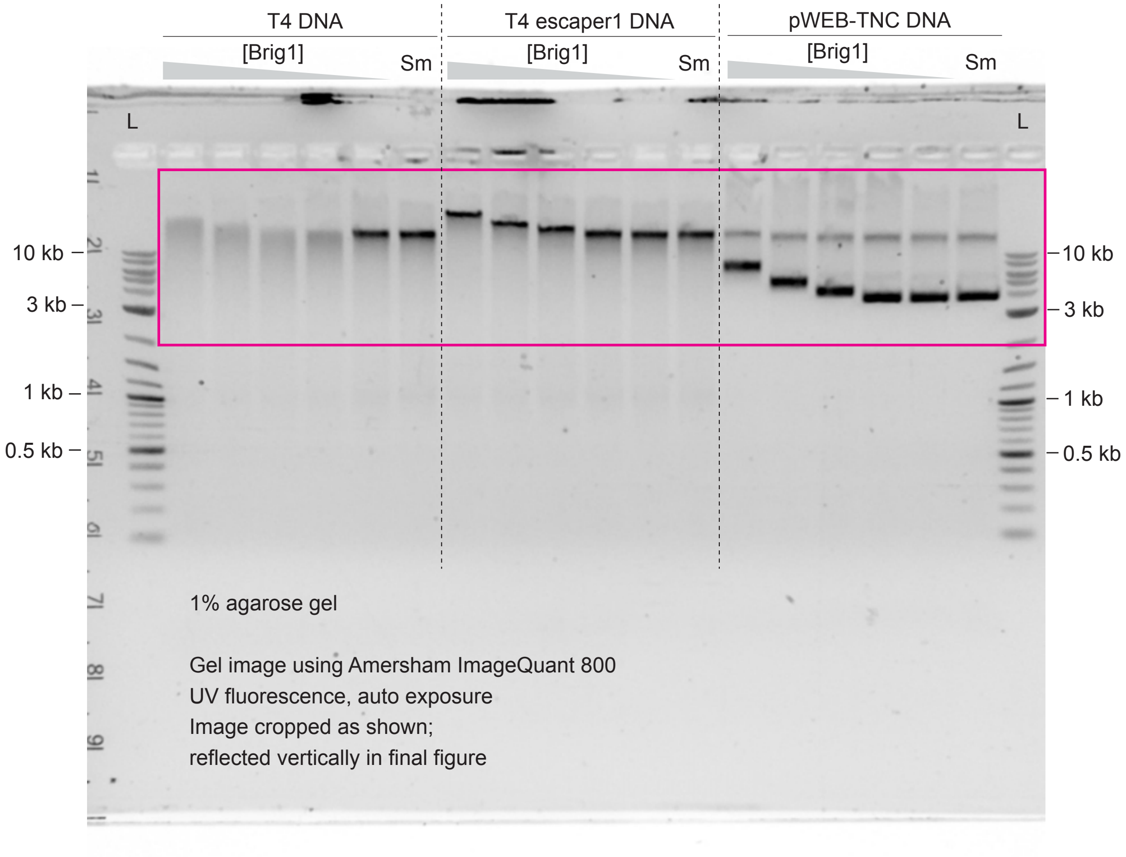


Brig1 concentrations: 800, 400, 200, 20, 2 nM
L = NEB 1 kb Plus DNA Ladder

**Fig. 3e**

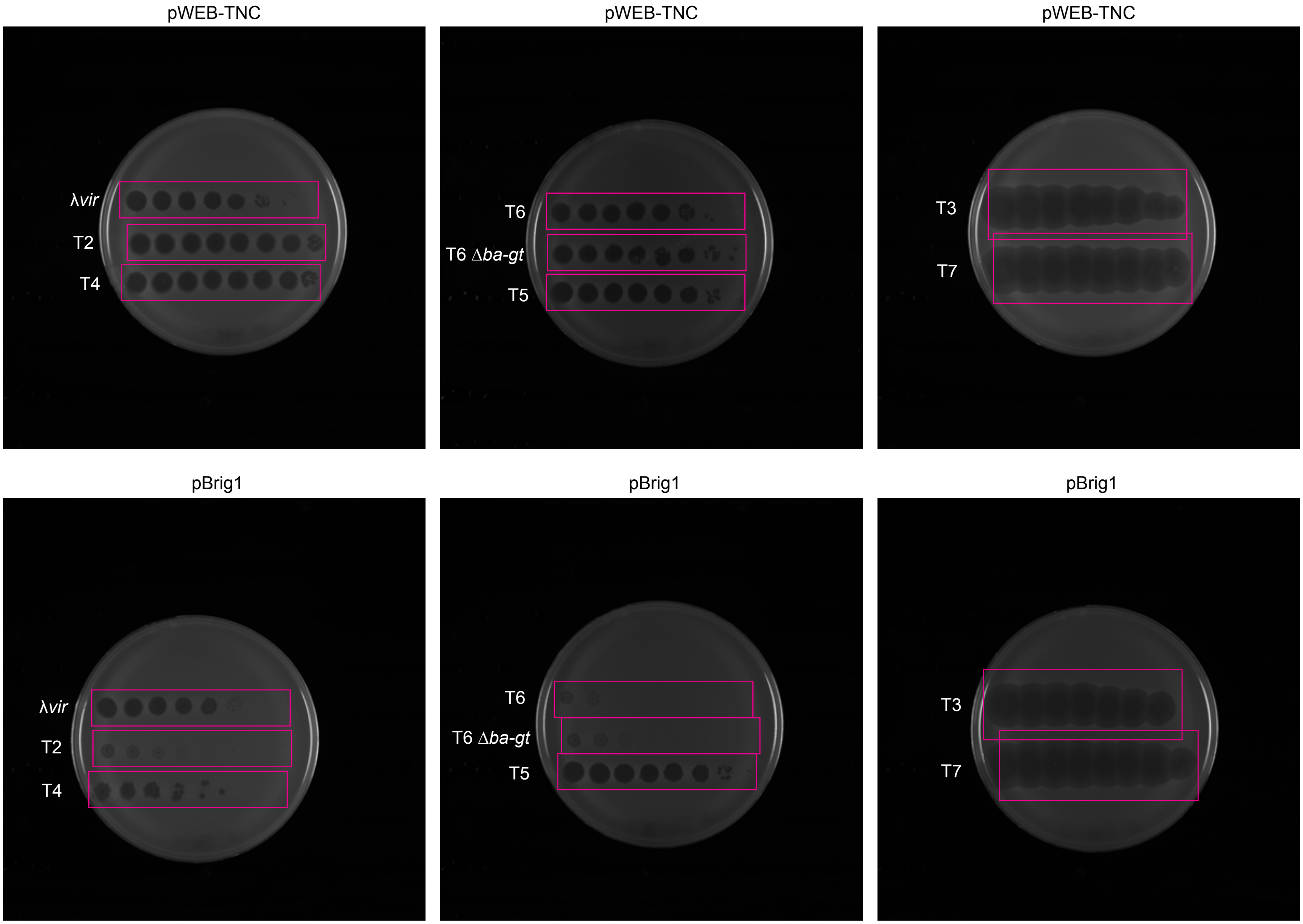


These are plate images for the representative images shown in Fig 4*a*. Images were cropped as shown and then auto contrasted on Adobe Photoshop; pBrig1 rows for phages T2, T6 and T6 Δ*ba-gt* were further enhanced for brightness to enable better visibility. T3 and T7 images were manually adjusted for brightness and contrast using Adobe Photoshop (same settings in each case).

**Fig. 4a**

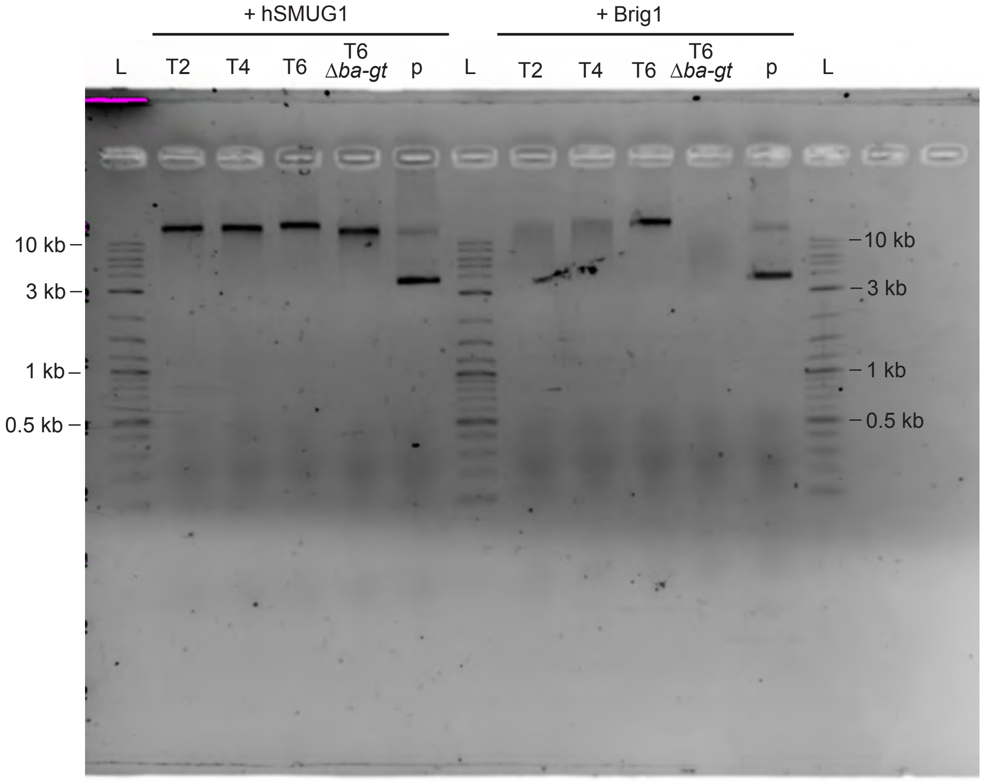


1% agarose gel

Gel image using Amersham ImageQuant 800 UV fluorescence, auto exposure

Brig1 concentration: 100 nM
L = NEB 1 kb Plus DNA Ladder

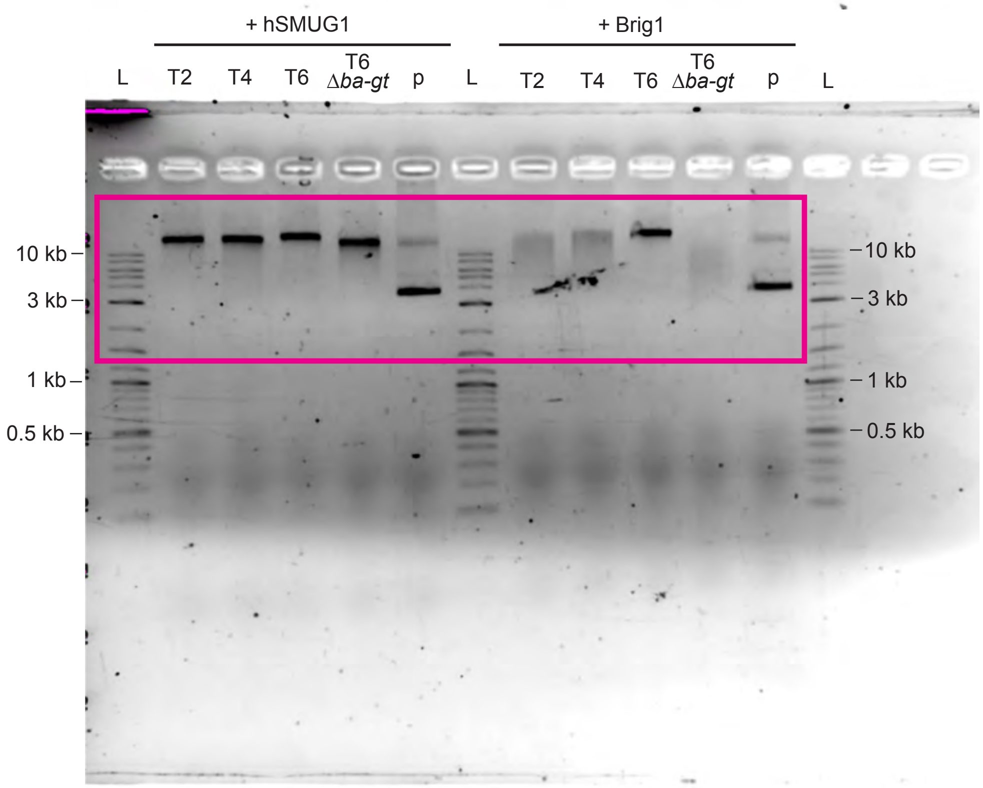


Gel image from above with brightness and contrast adjusted on gel imager; pink box shows cropped image; cropped image auto contrasted on Adobe Photoshop

**Fig. 4b**

*Nocardioides zhouii*
WP 129427366.1
pAM38(*brig1 homolog*) - Arabinose

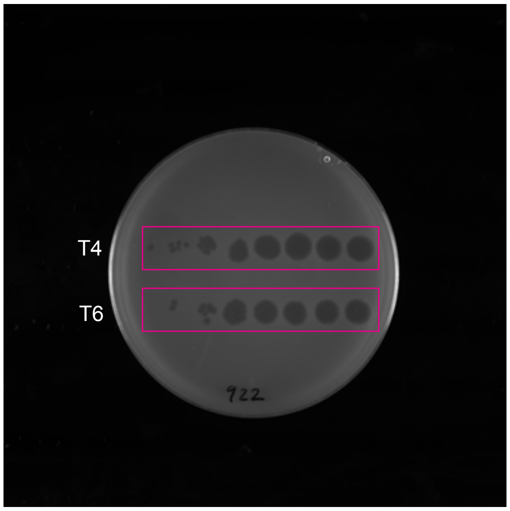


*Nocardioides zhouii*
WP 129427366.1
pAM38(*brig1 homolog*) + Arabinose

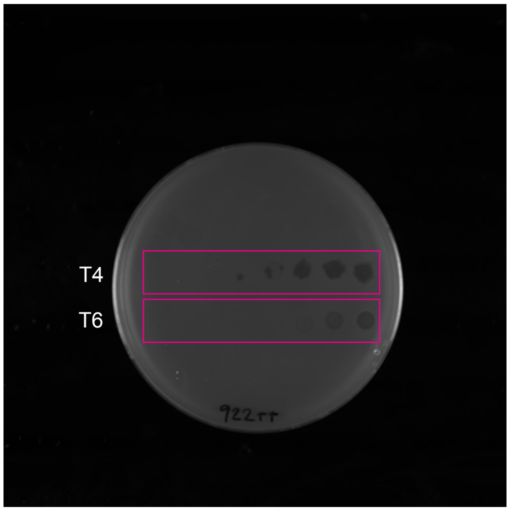


*Nocardioides anomalus*
WP 165228961.1
pAM38(*brig1 homolog*) - Arabinose

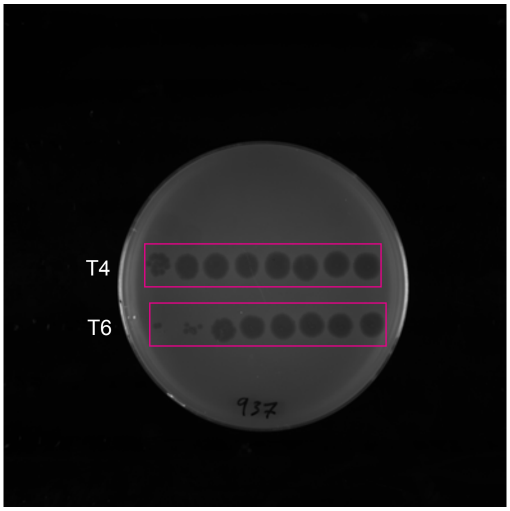


*Nocardioides anomalus*
WP 165228961.1
pAM38(*brig1 homolog*) + Arabinose

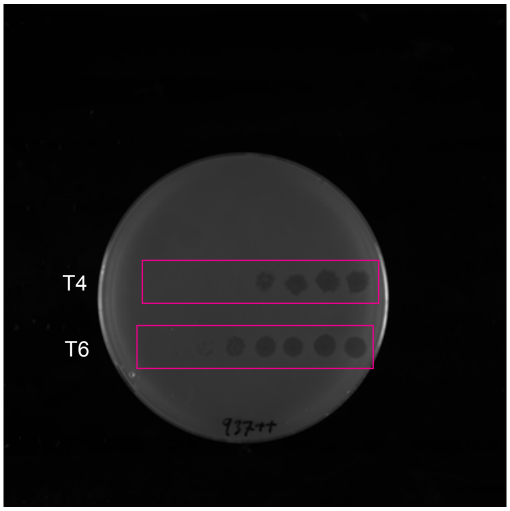


These are plate images for the representative images shown in Fig 5b. Images were cropped as shown and then auto contrasted on Adobe Photoshop; The *N. zhouii* Brig1 homolog overexpression row for phage T6 was further enhanced for brightness to enable better visibility. Images reflected vertically in figure.

**Fig. 5b**

Representative false positive colony plaque assay | Representative resistant clone plaque assay

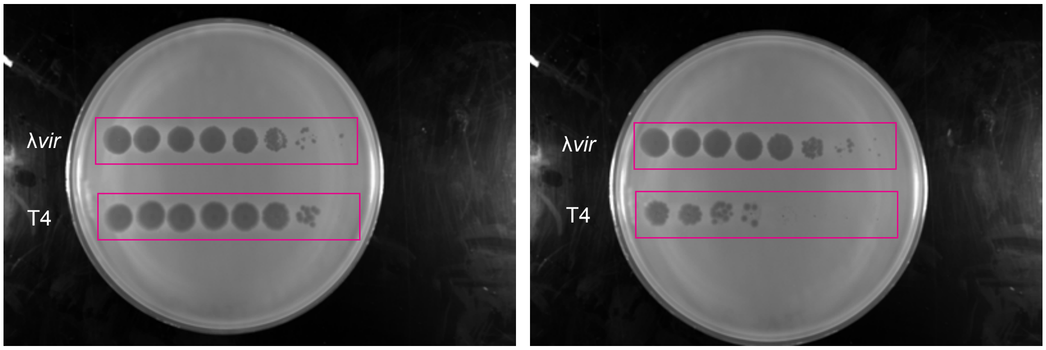


These are plate images for the representative images shown in Extended Data Fig 1b. Images were cropped as shown and then auto contrasted on Adobe Photoshop.

## Extended Data Fig. 1b

pWEB-TNC/Phage T4

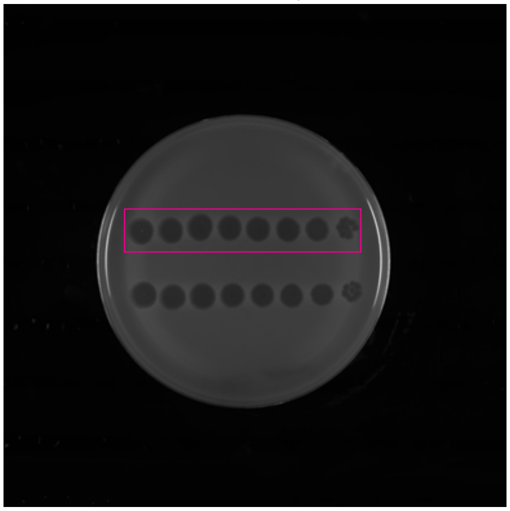

pFragmentC/Phage T4

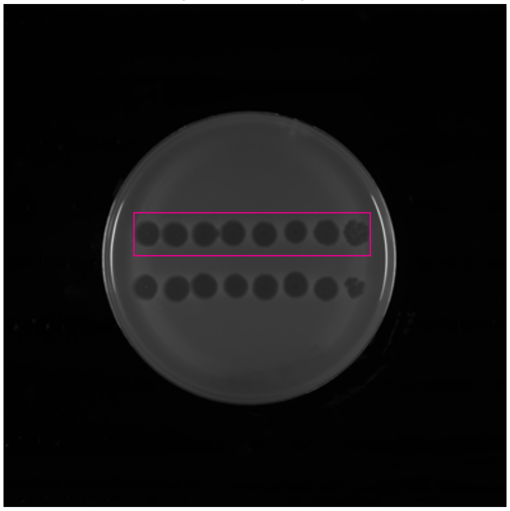

pFragmentD/Phage T4

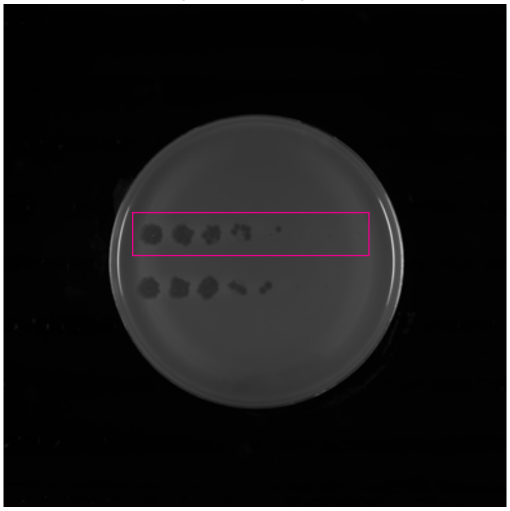

These are plate images for the representative images shown in Extended Data Fig 1d. Images were cropped as shown and then auto contrasted on Adobe Photoshop.

## Extended Data Fig. 1d

pWEB-TNC/Phage T4

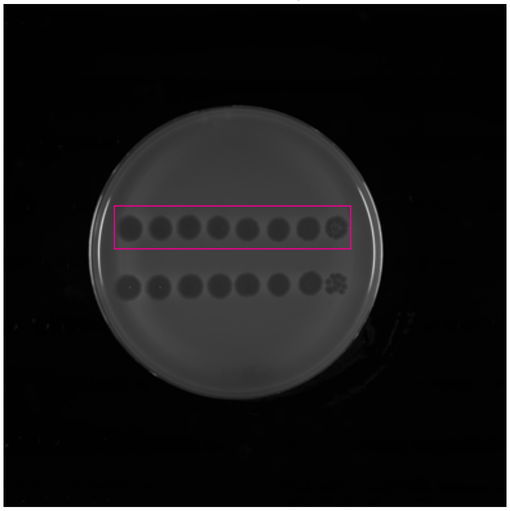

pFragmentD1/Phage T4

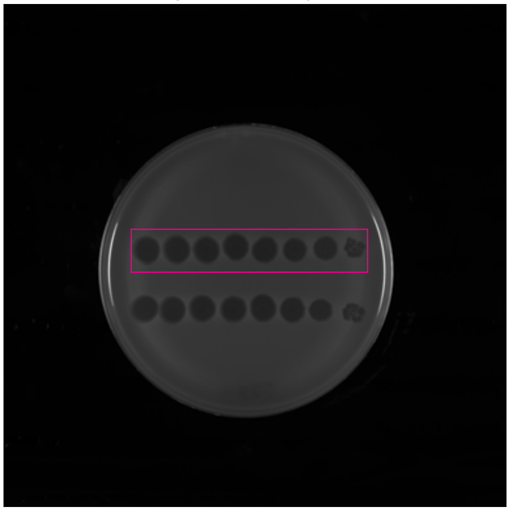

pFragmentD2/Phage T4

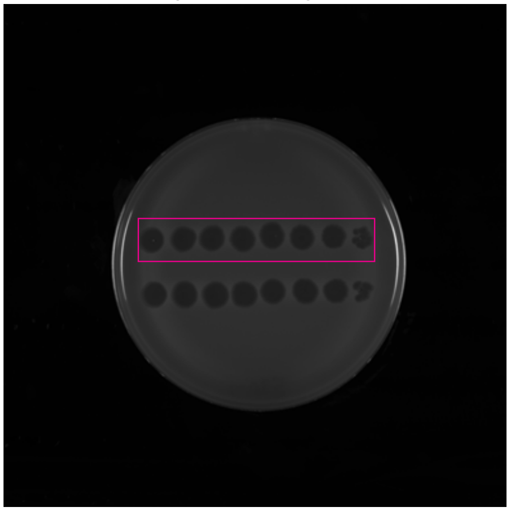

pFragmentD3/Phage T4

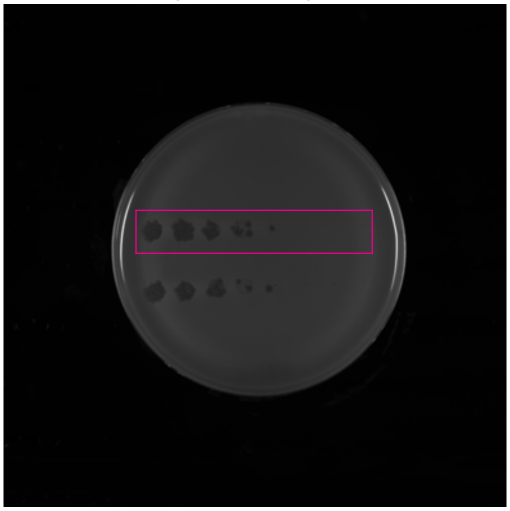

These are plate images for the representative images shown in Extended Data Fig 1f. Images were cropped as shown and then auto contrasted on Adobe Photoshop.

**Extended Data Fig. 1f**

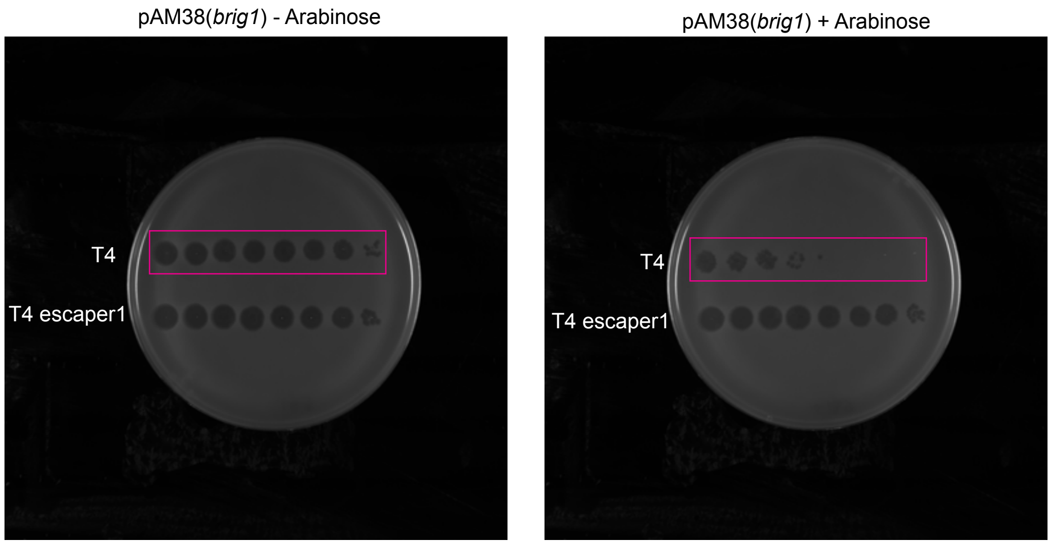


These are plate images for the representative images shown in Extended Data Fig 1g. Images were cropped as shown and then auto contrasted on Adobe Photoshop.

**Extended Data Fig. 1g**

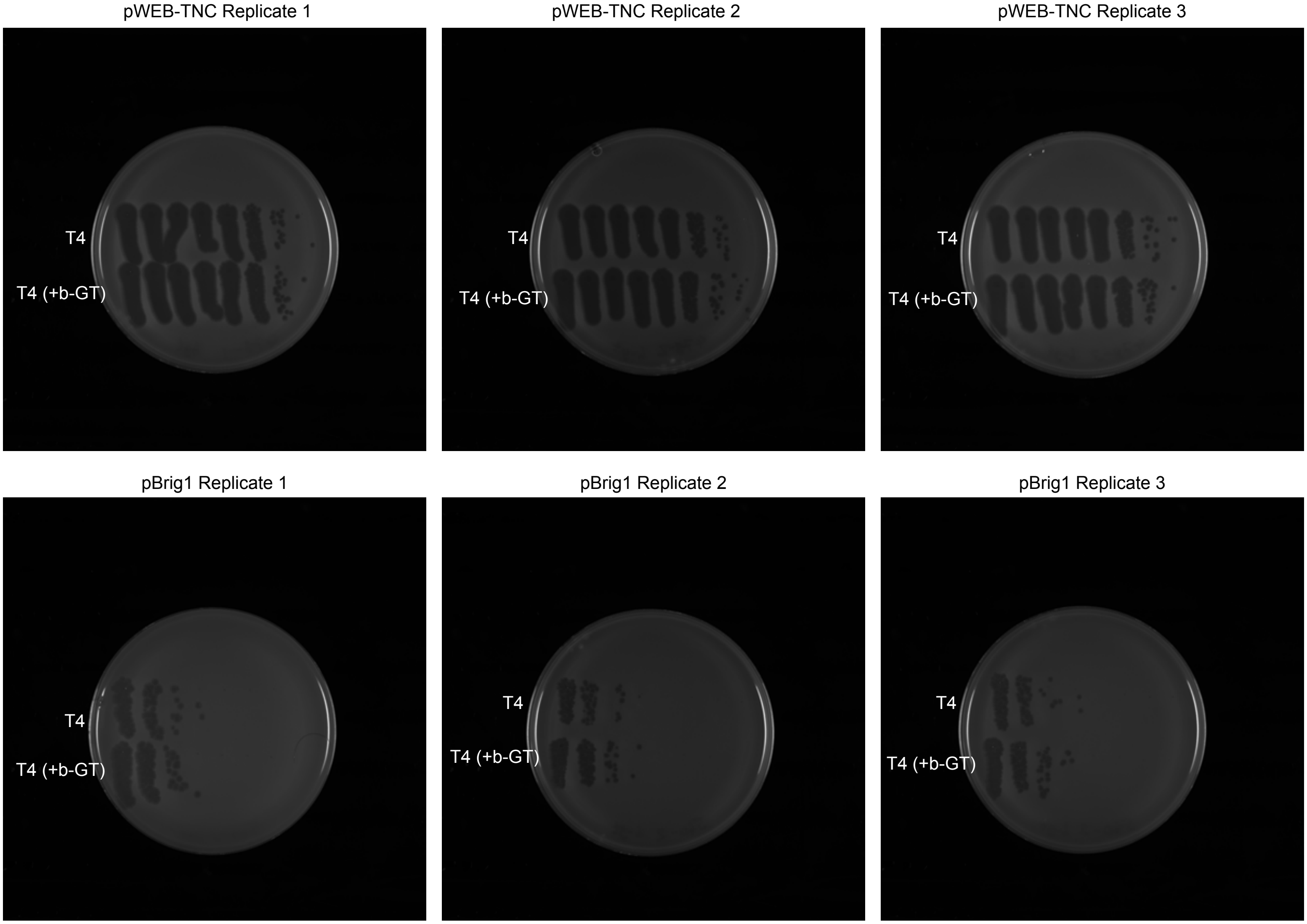


These are plate images for the quantification shown in Extended Data Fig 2i. Plaques were quantified using the counting tool in FIJI (ImageJ).

# Extended Data Fig. 2i

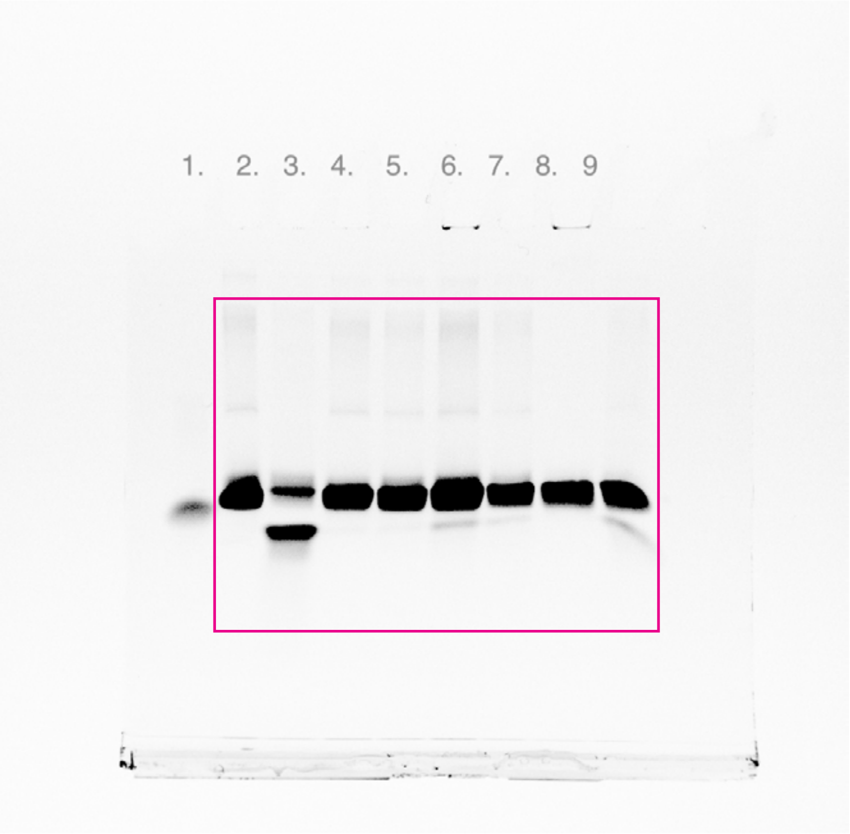


10% TBE gel
Imaged using ChemiDoc MP Imager (BioRad)
605/50 filter for UV fluorescence
(ethidium bromide)

Image cropped as shown

Annealed b-GT or a-GT treated ssDNA with complementary bottom strand ssDNA oligonucleotide.
Then incubated all samples overnight with MfeI.

Samples as below,
using oligonucleotide from Extended Data Fig. 3c:
1: ssDNA with hmC, no MfeI enzyme
2: dsDNA with hmC in top strand, no MfeI enzyme
From 3-9, each sample digested with MfeI enzyme
3: dsDNA with hmC in top strand
4: dsDNA with hmC in top strand; top strand ssDNA glucosylated with b-GT
5: dsDNA with hmC in top strand; dsDNA glucosylated with b-GT
6: dsDNA with hmC in top strand; top strand ssDNA glucosylated with a-GT (low conc.)
7: dsDNA with hmC in top strand; dsDNA glucosylated with a-GT (low conc.)
8: dsDNA with hmC in top strand; top strand ssDNA glucosylated with a-GT (high conc.)
9: dsDNA with hmC in top strand; dsDNA glucosylated with a-GT (high conc.)

**Extended Data Fig. 3e**

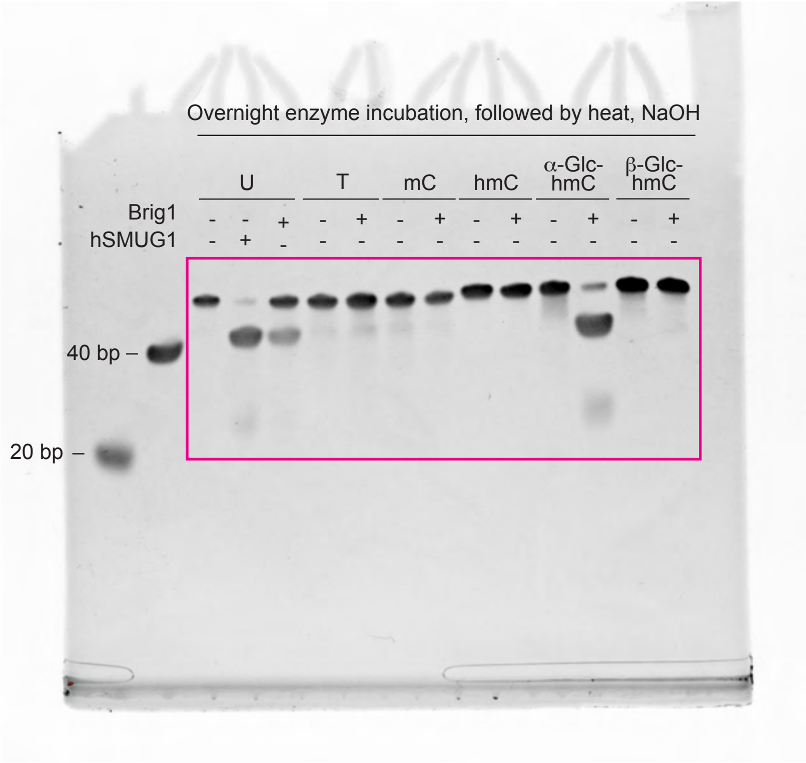


10% TBE gel
Imaged using ChemiDoc MP Imager (BioRad)
605/50 filter for UV fluorescence
(ethidium bromide)

40 and 20 bp oligos used as molecular weight markers

Image cropped as shown

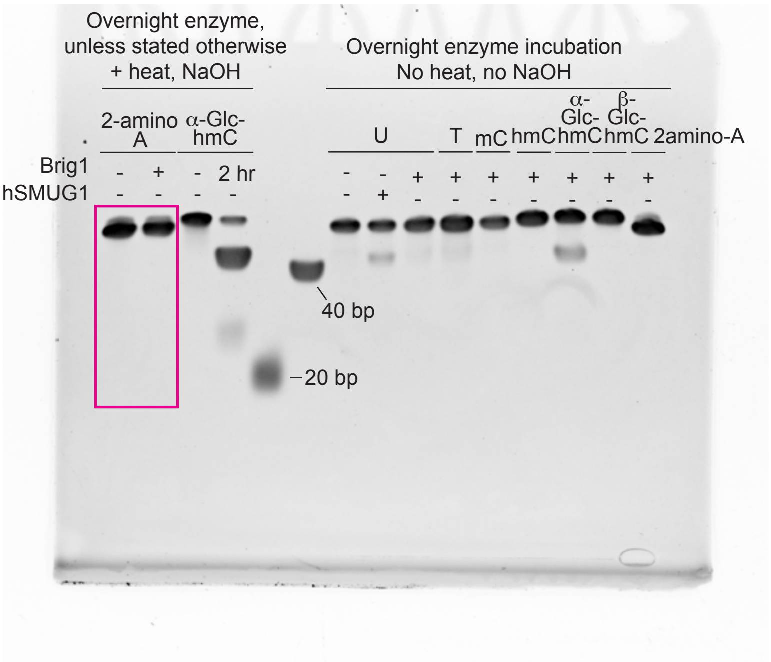


10% TBE gel
Imaged using ChemiDoc MP Imager (BioRad)
605/50 filter for UV fluorescence
(ethidium bromide)

40 and 20 bp oligos used as molecular weight markers

Image cropped as shown

# Extended Data Fig. 3h

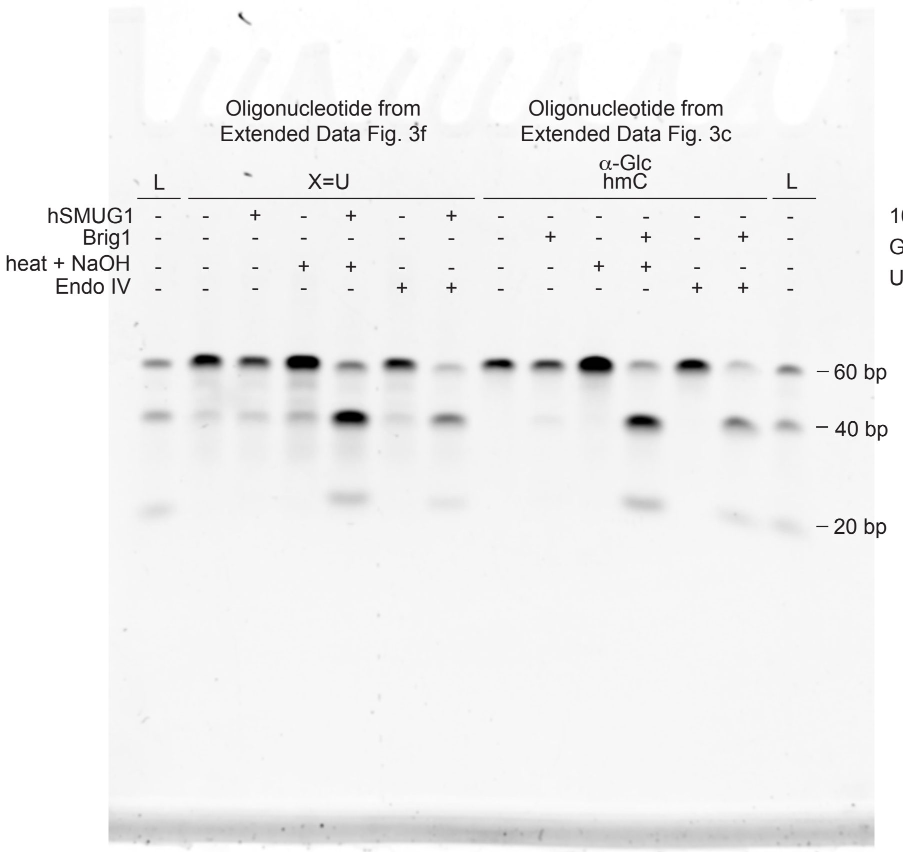


10% TBE Gel

Gel image using Amersham ImageQuant 800

UV fluorescence, auto exposure

L = ssDNA size ladder

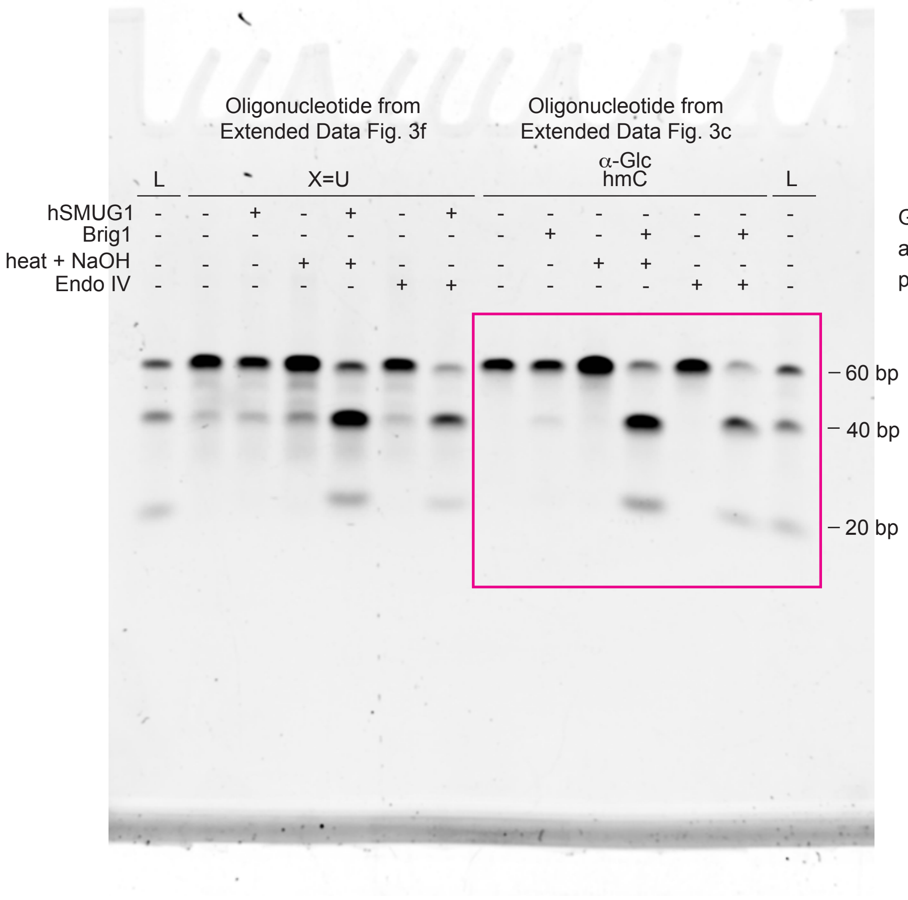


Gel image from above with brightness and contrast adjusted on gel imager; pink box shows cropped image

**Extended Data Fig. 3i**

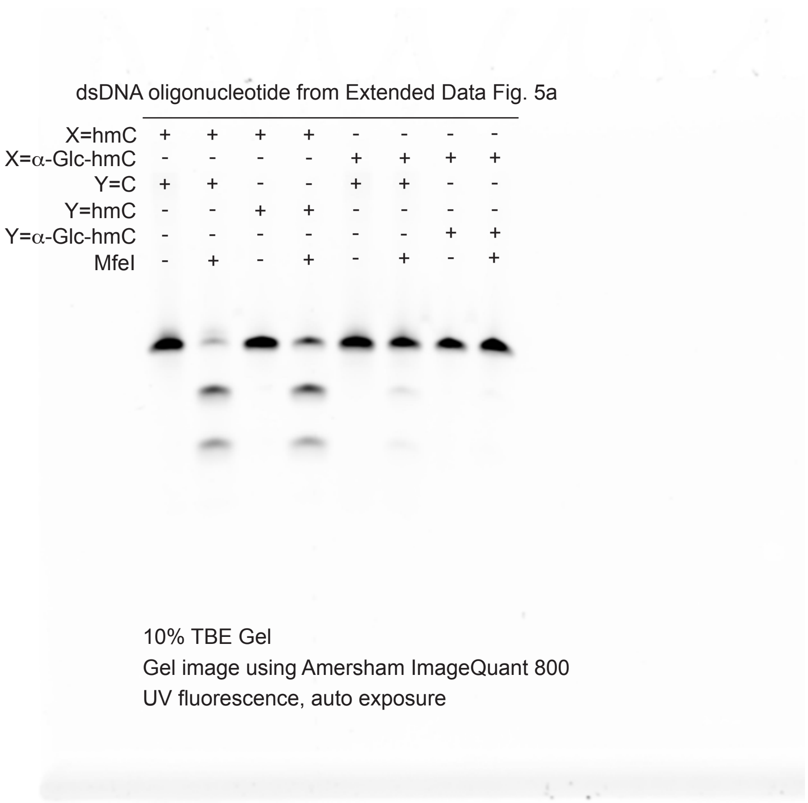


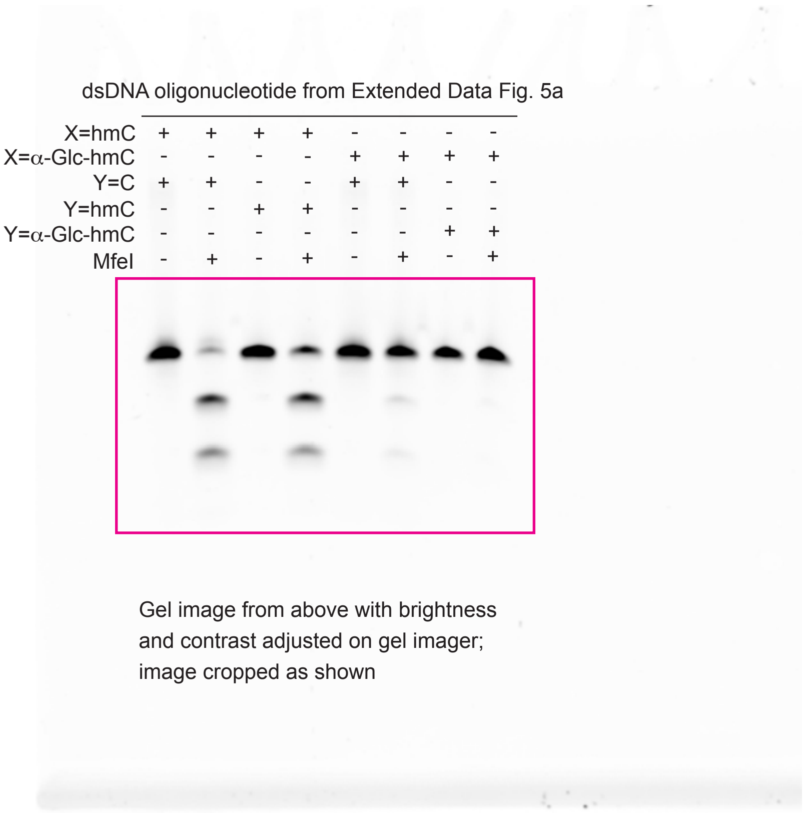


**Extended Data Fig. 5c**

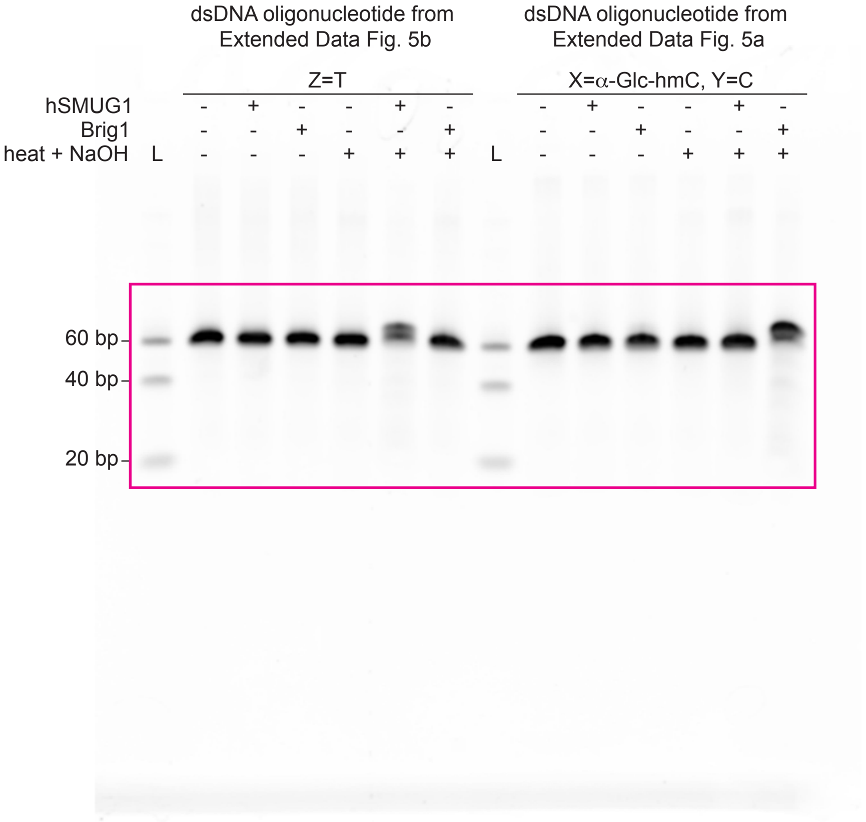


10% TBE Gel
Gel image using Amersham ImageQuant 800
UV fluorescence, auto exposure
Image cropped as shown

L = dsDNA size ladder

**Extended Data Fig. 5d**

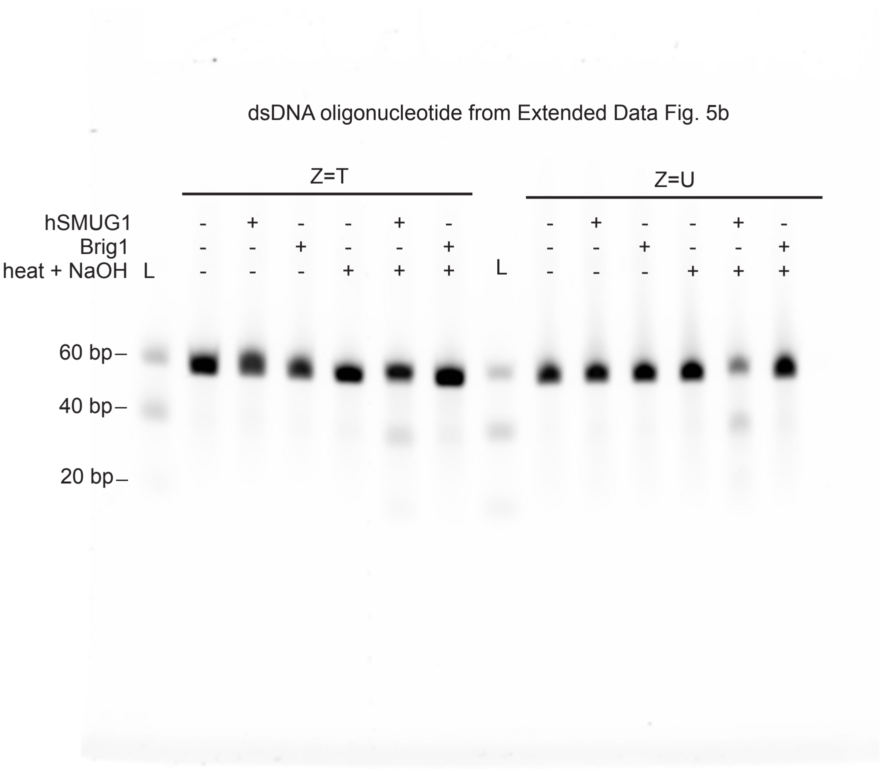


6% TBE-Urea Gel
Gel image using Amersham ImageQuant 800
UV fluorescence, auto exposure

L = ssDNA size ladder

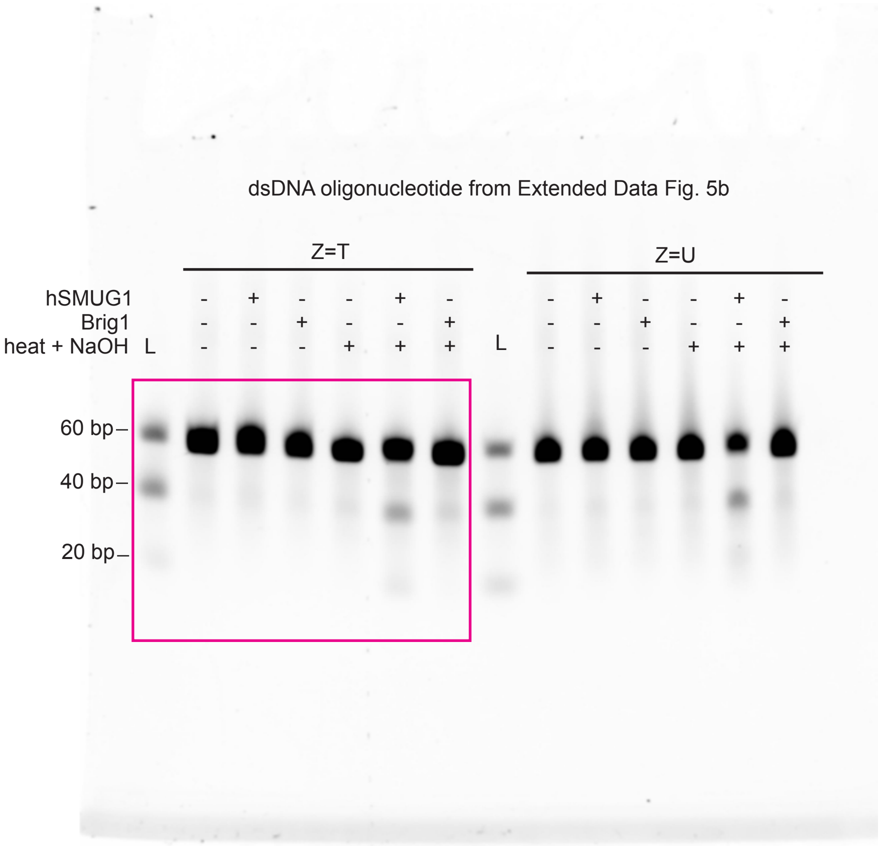


Gel image from above with brightness and contrast adjusted on gel imager; pink box shows cropped image; image brightness of cropped section was decreased using Adobe Photoshop for clearer band visibility

**Extended Data Fig. 5e**

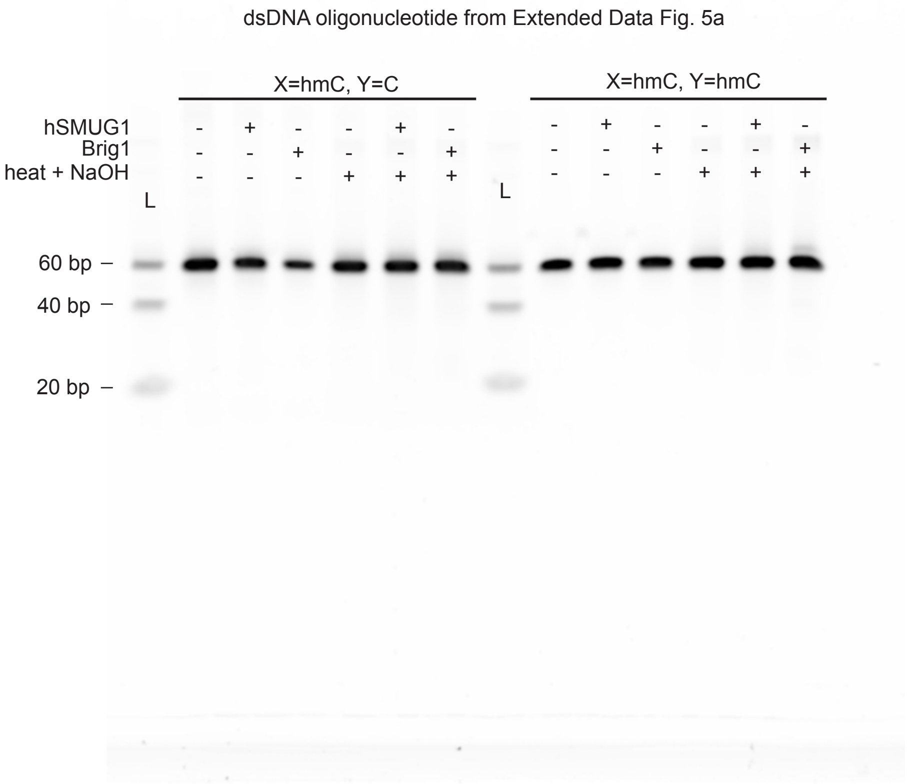


10% TBE Gel
Gel image using Amersham ImageQuant 800
UV fluorescence, auto exposure

L = dsDNA size ladder

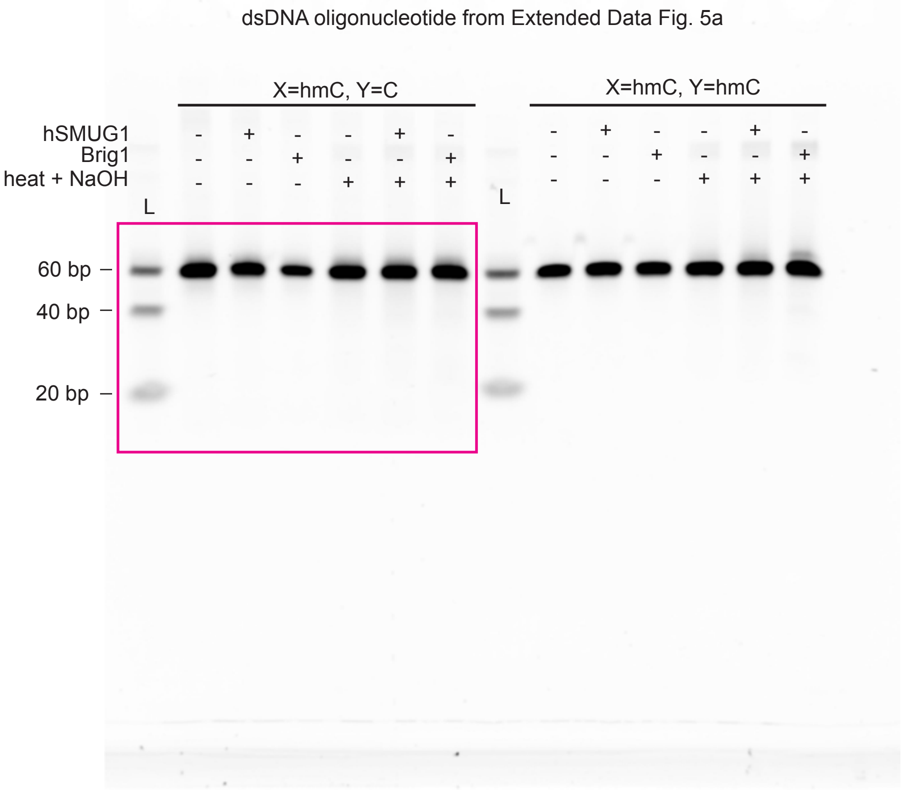


Gel image from above with brightness and contrast adjusted on gel imager; image cropped as shown

**Extended Data Fig. 5f**

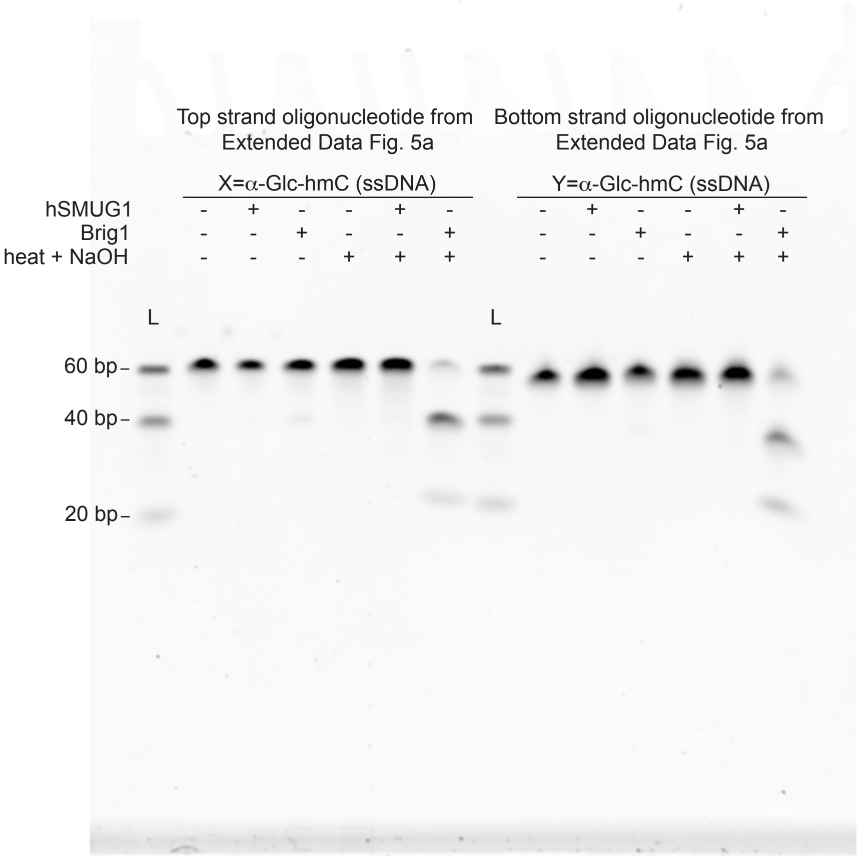


10% TBE Gel
Gel image using Amersham ImageQuant 800
UV fluorescence, auto exposure

L = ssDNA size ladder

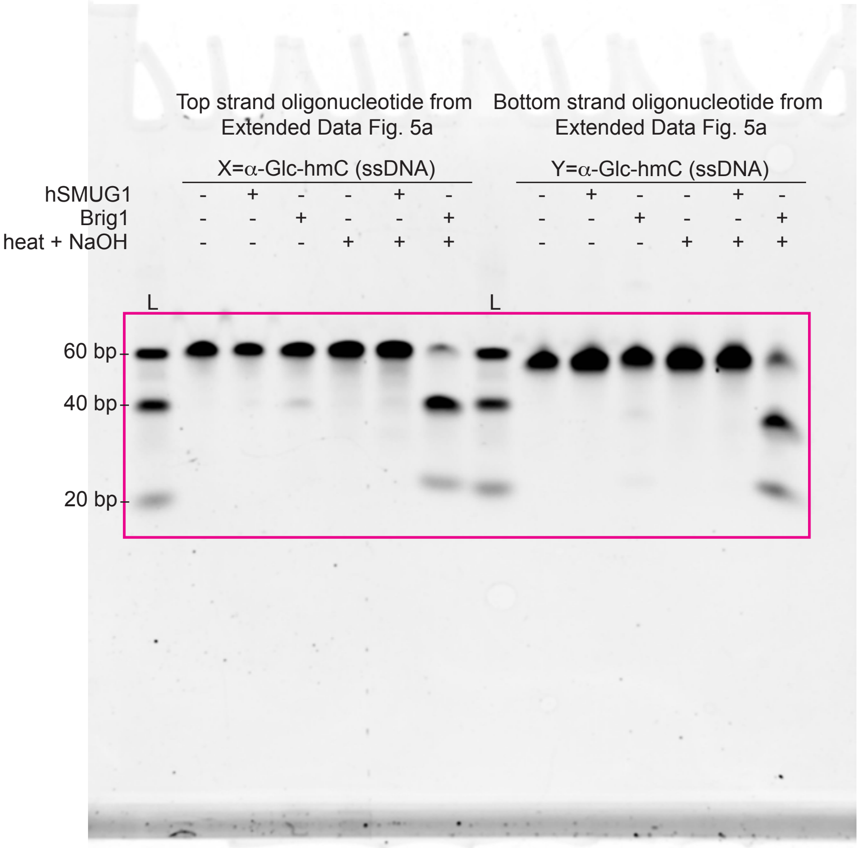


Gel image from above with brightness and contrast adjusted on gel imager; image cropped as shown; image auto contrasted on Adobe Photoshop before pasting in final figure

**Extended Data Fig. 5g**

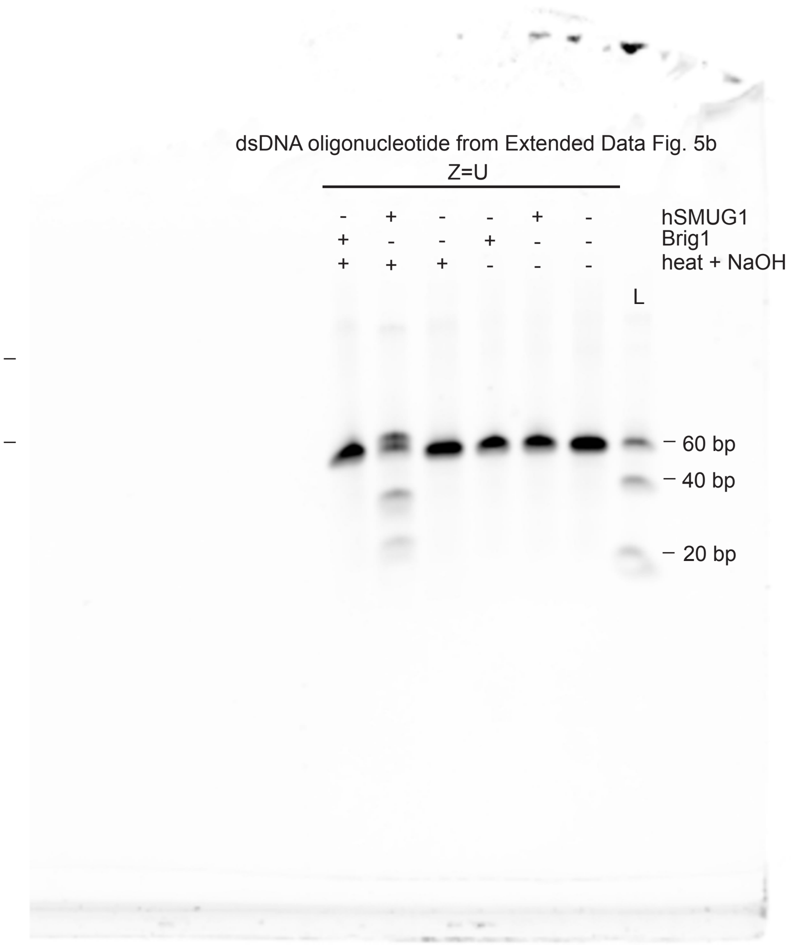


10% TBE Gel
Gel image using Amersham ImageQuant 800
UV fluorescence, auto exposure

L = dsDNA size ladder

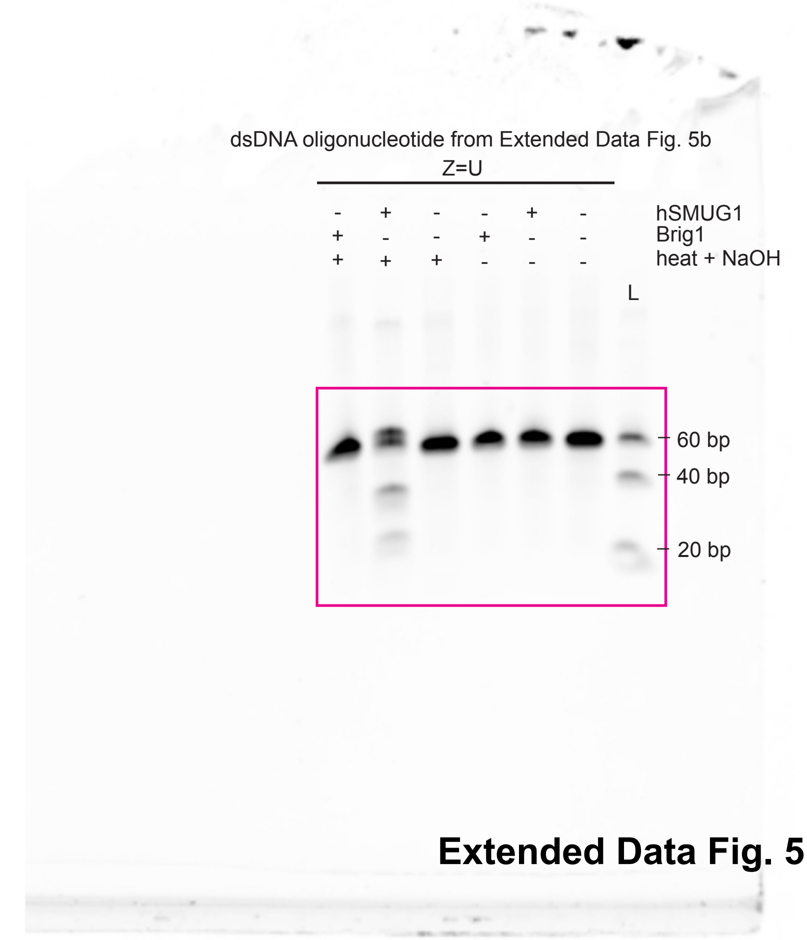


Gel image from above with brightness and contrast adjusted on gel imager; image cropped as shown; cropped image reflected vertically in final figure

**Extended Data Fig. 5h**

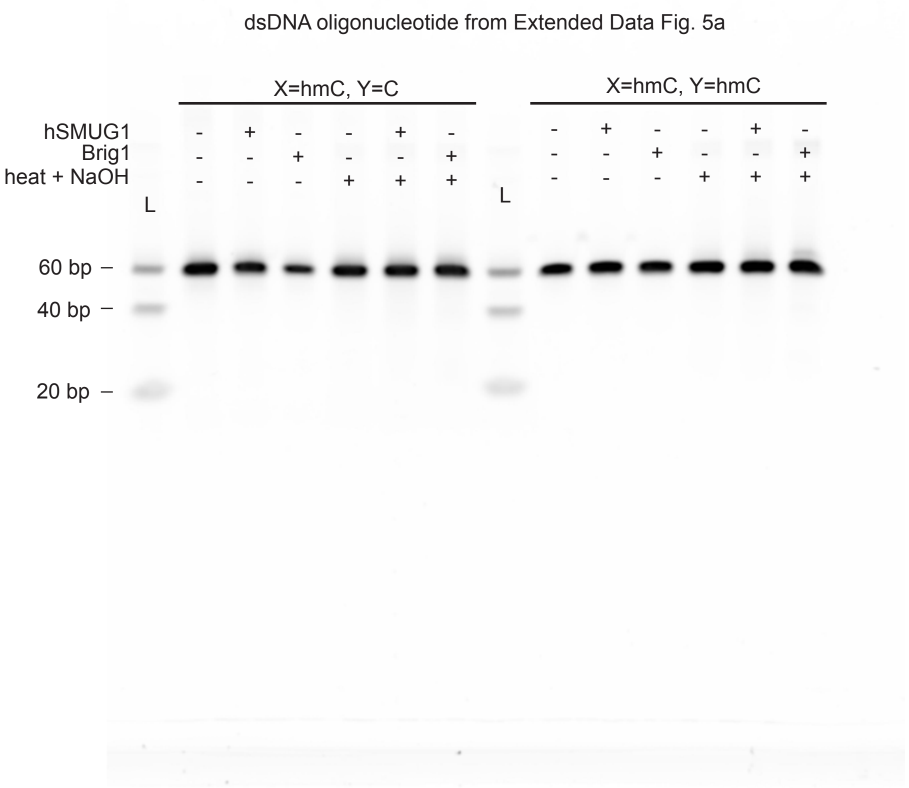


Exact same gel as Extended Data Fig. 5f
10% TBE Gel
Gel image using Amersham ImageQuant 800
UV fluorescence, auto exposure

L = dsDNA size ladder

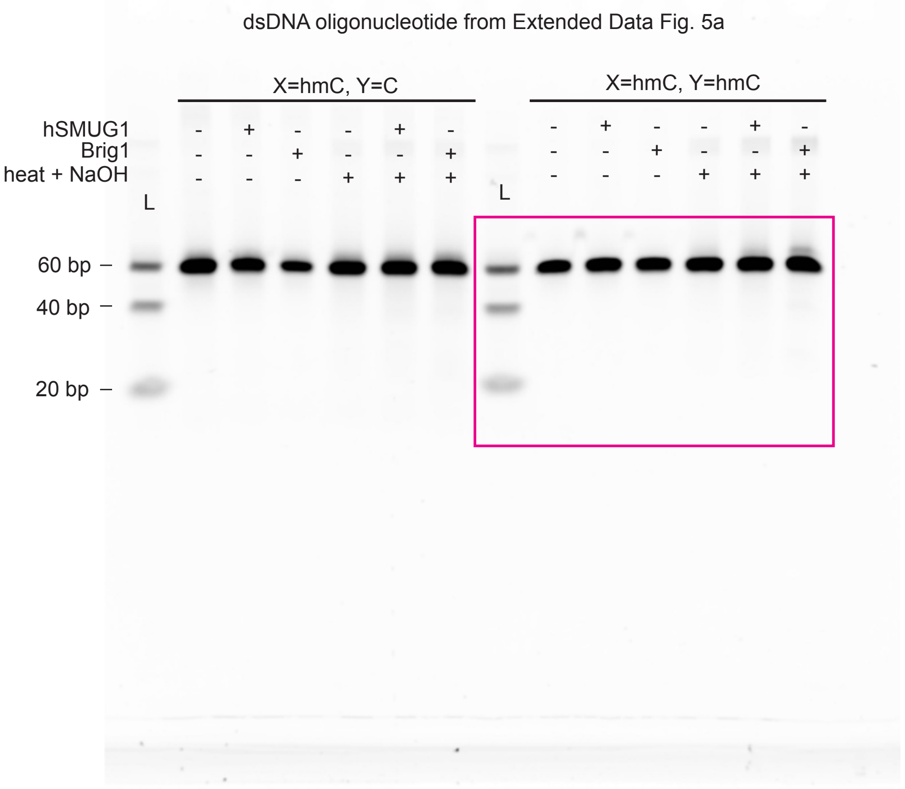


Gel image from above with brightness and contrast adjusted on gel imager;
Exact same image as used for Extended Data Fig. 5f but with different cropped segment;
image cropped as shown

**Extended Data Fig. 5i**

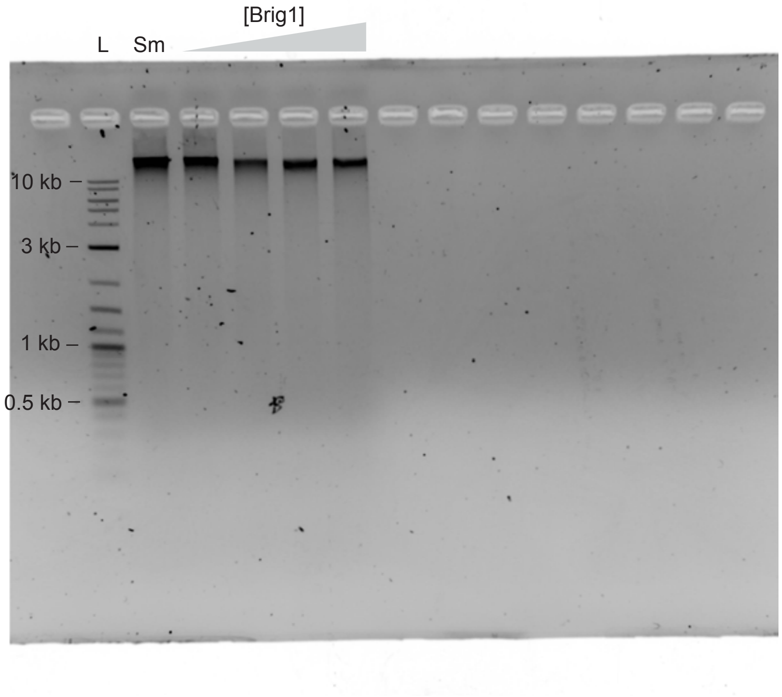


1% agarose gel
Electrophoresis at 40 V for 3 hours at 4°C

Gel image using Amersham ImageQuant 800
UV fluorescence, auto exposure

Brig1 concentrations: 2, 20, 200, 400 nM
L = NEB 1 kb Plus DNA Ladder

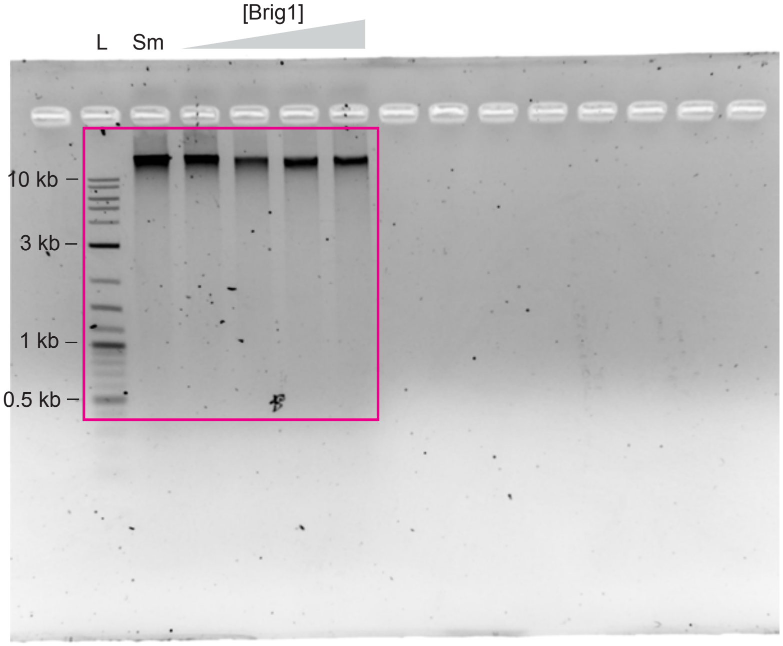


Gel image from above with brightness
and contrast adjusted on gel imager;
pink box shows cropped image

**Extended Data Fig. 6a**

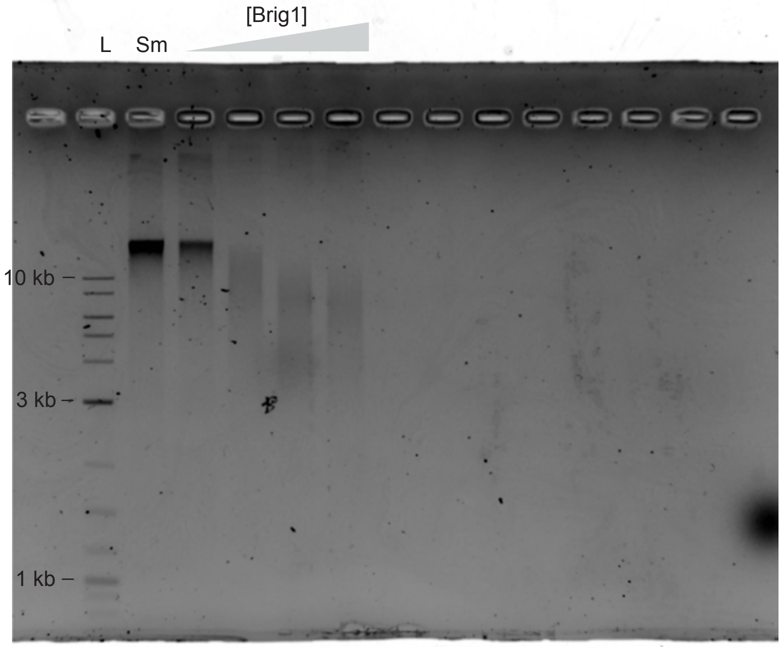


1% agarose gel
Same gel as previous page;
run under higher voltage
(85, 150 and 200 V for additional
25, 8 and 8 minutes, respectively)
at room temperature.

Gel image using Amersham ImageQuant 800
UV fluorescence, auto exposure

Brig1 concentrations: 2, 20, 200, 400 nM
L = NEB 1 kb Plus DNA Ladder

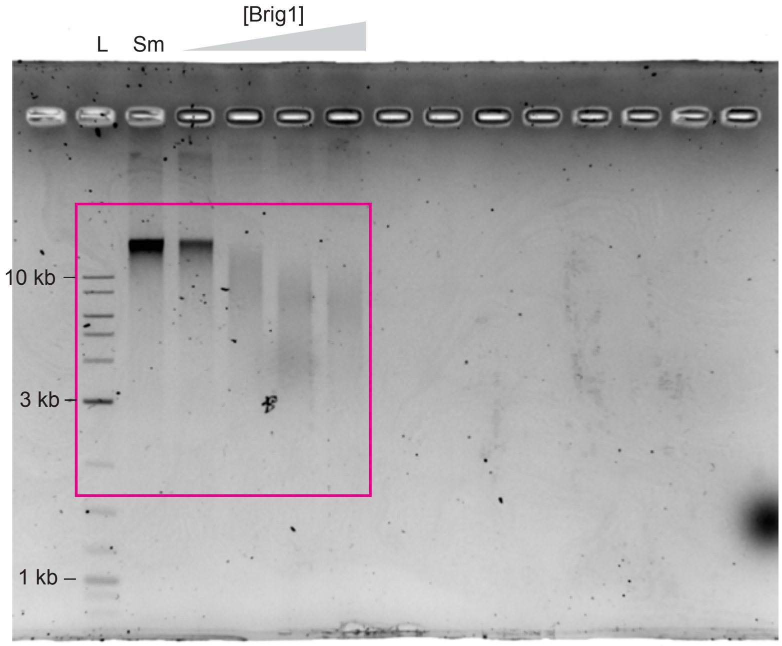


Gel image from above with brightness
and contrast adjusted on gel imager;
pink box shows cropped image

**Extended Data Fig. 6b**

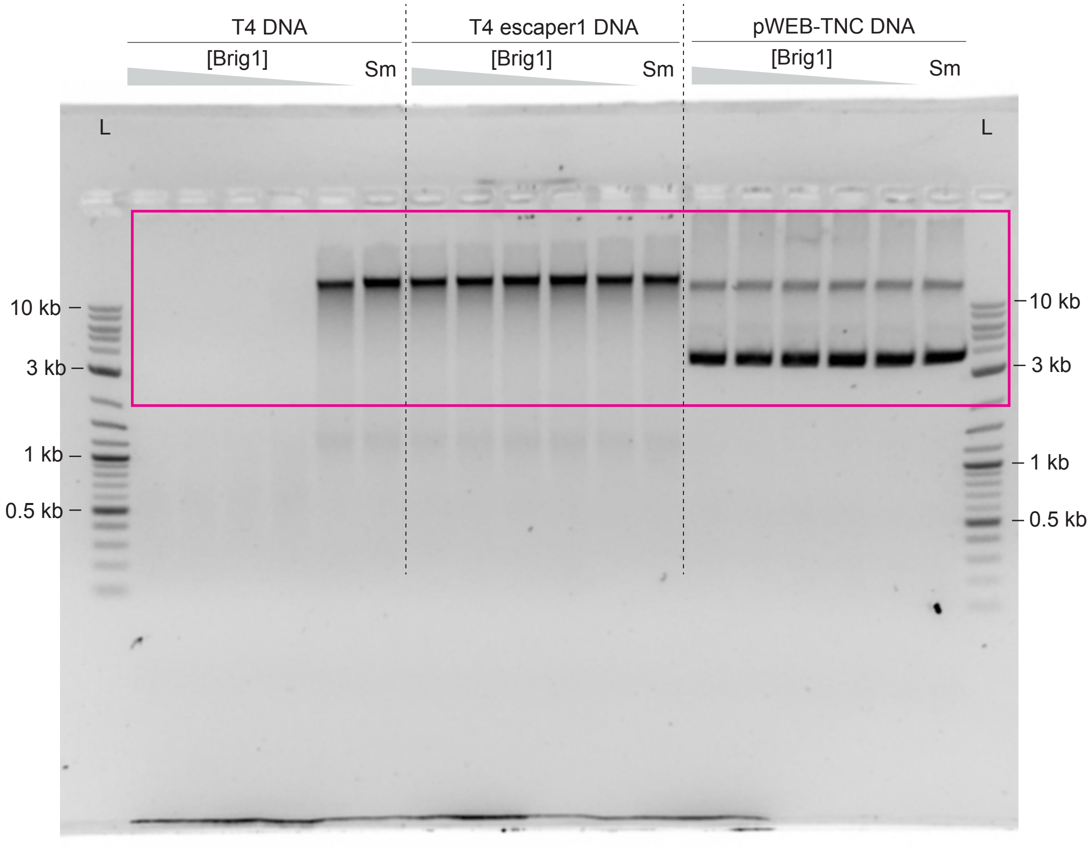


1% agarose gel

Gel image using Amersham ImageQuant 800
UV fluorescence, auto exposure
Image cropped as shown;
reflected vertically in final figure

Brig1 concentrations: 800, 400, 200, 20, 2 nM
L = NEB 1 kb Plus DNA Ladder

## Extended Data Fig. 6c

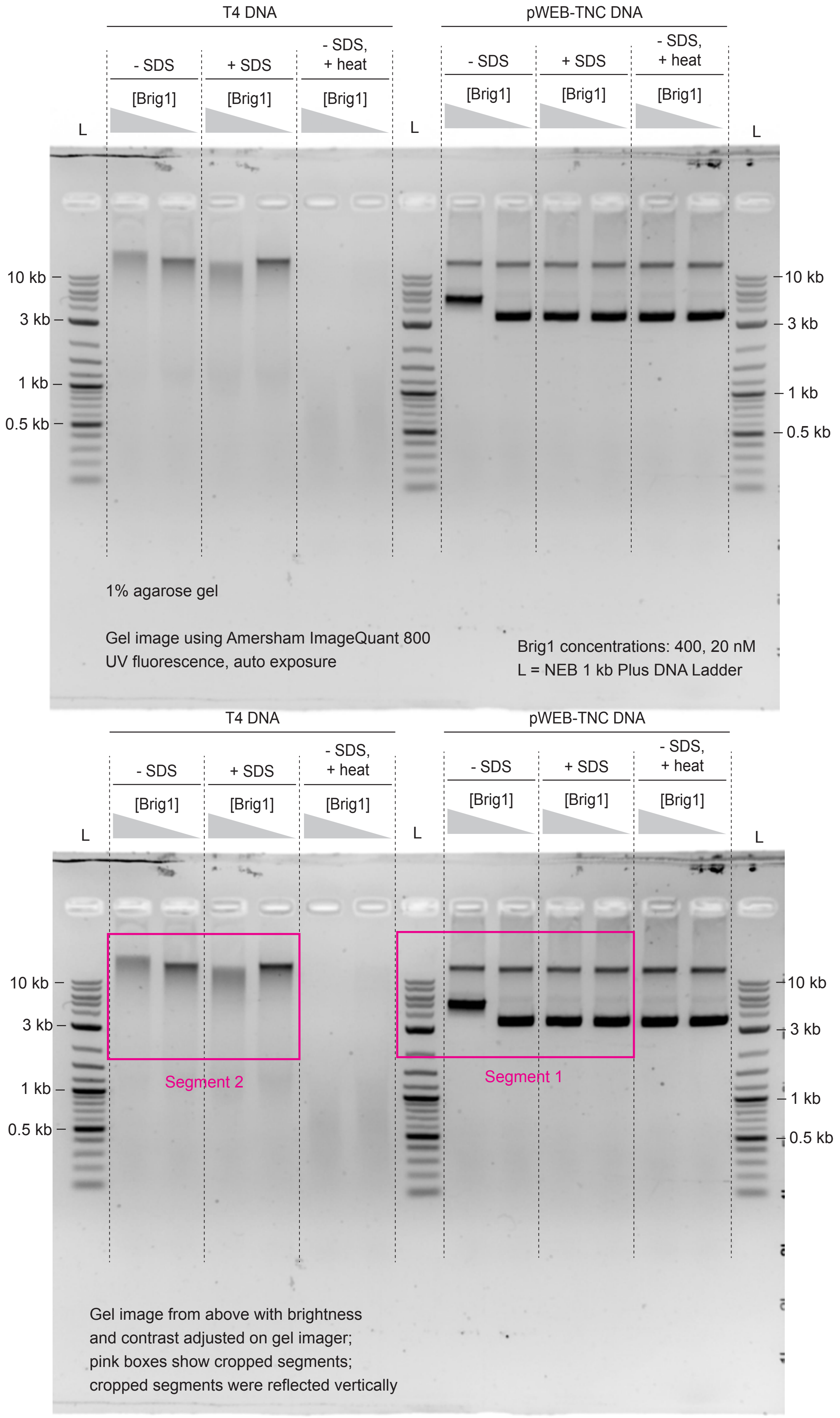


**Extended Data Fig. 6d**

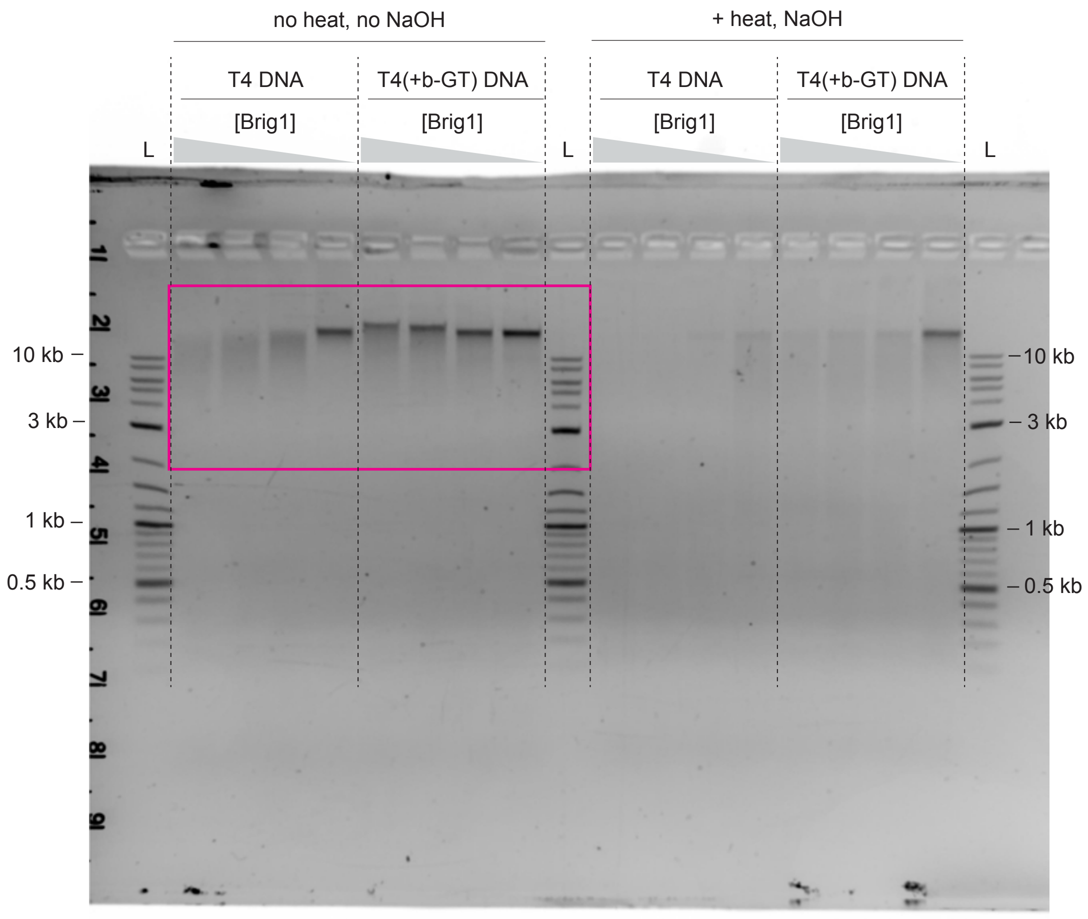


1% agarose gel

Gel image using Amersham ImageQuant 800
UV fluorescence, auto exposure
Image cropped as shown;
reflected vertically in final figure

Brig1 concentrations: 400, 200, 20, 2 nM
L = NEB 1 kb Plus DNA Ladder

**Extended Data Fig. 6e**

pWEB-TNC

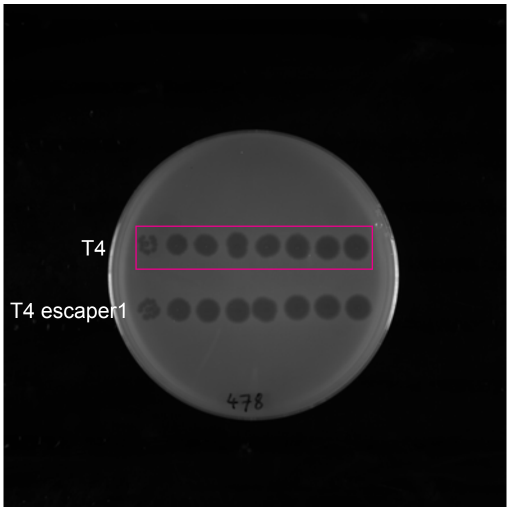


pBrig1

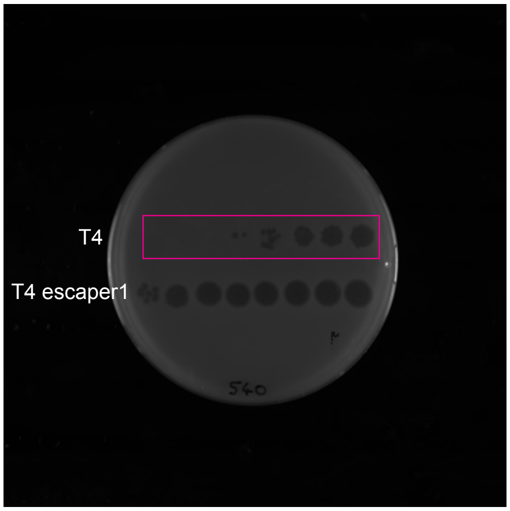


pBrig1(Y121A)

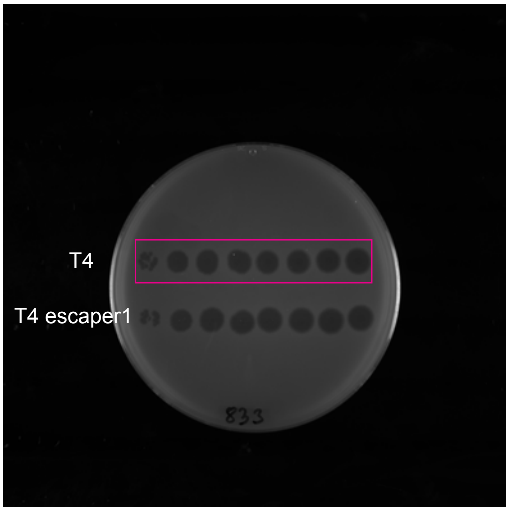


pBrig1(N145A)

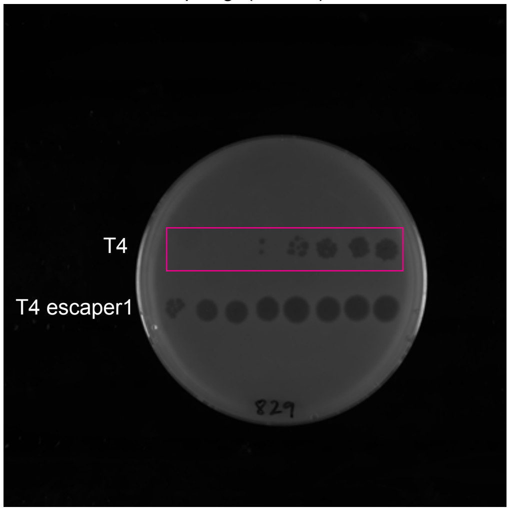


pBrig1(E147A)

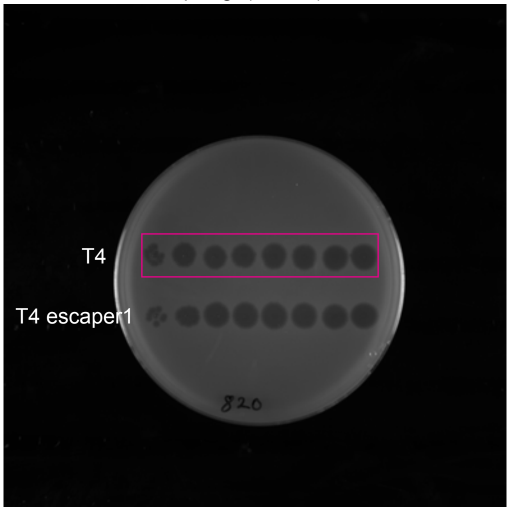


pBrig1(E147Q)

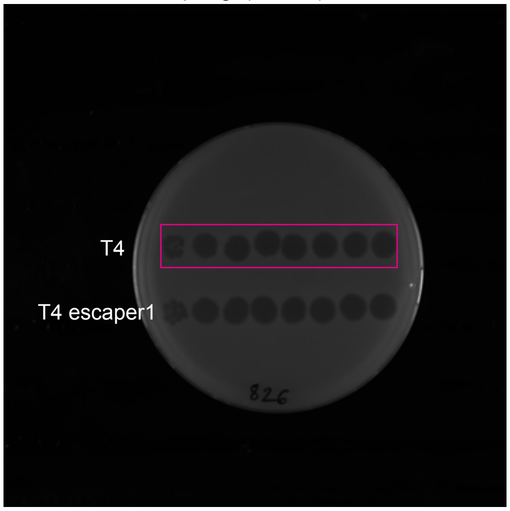


These are plate images for the representative images shown in Extended Data Fig 7a. Images were cropped as shown and then auto contrasted on Adobe Photoshop; image reflected vertically for final figure.

# Extended Data Fig. 7a

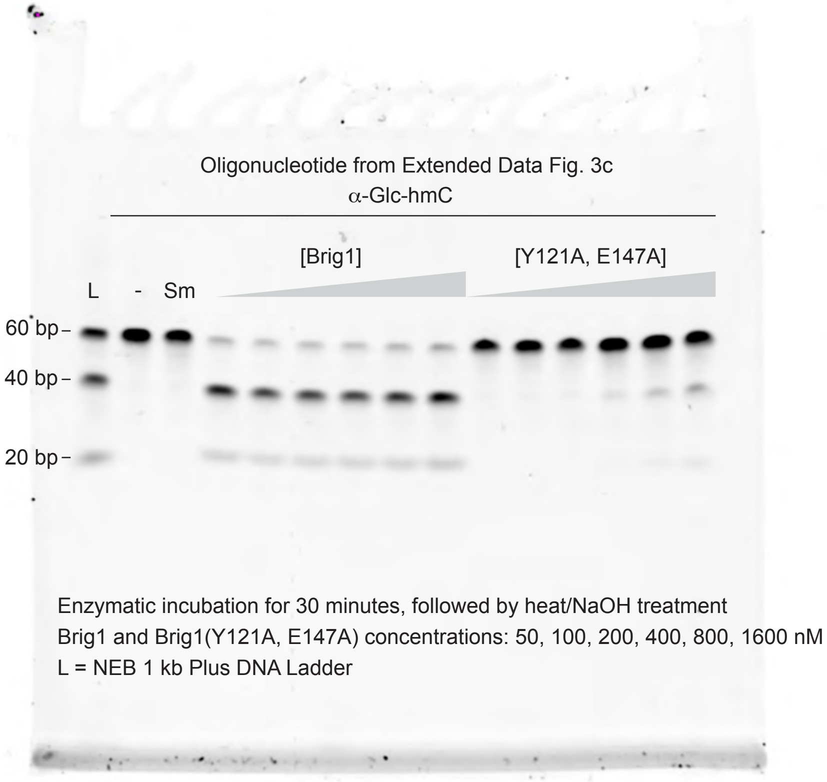


10% TBE gel

Gel image using Amersham ImageQuant 800
UV fluorescence, auto exposure

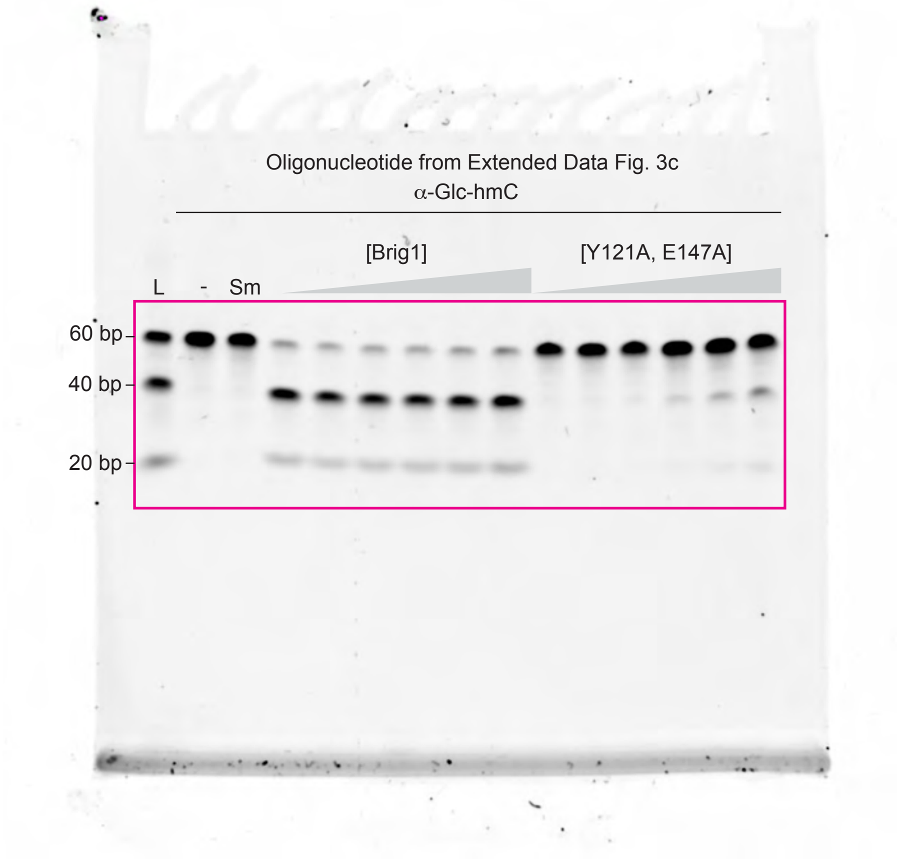


Gel image from above with brightness
and contrast adjusted on gel imager;
pink box shows cropped image

**Extended Data Fig. 7b**

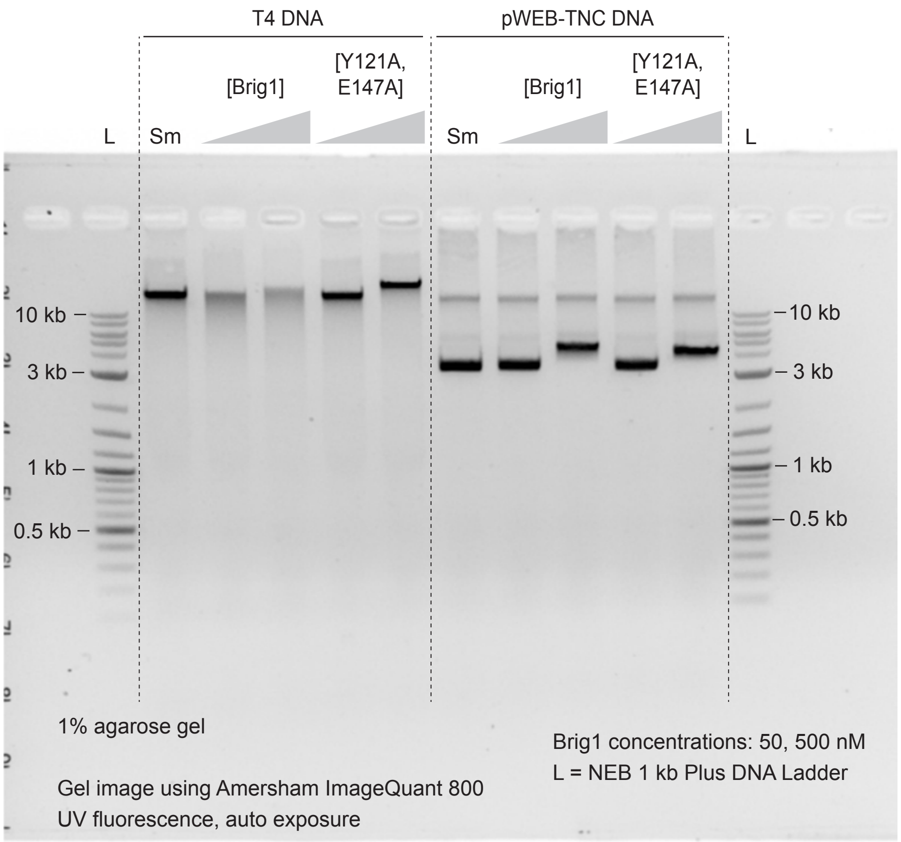


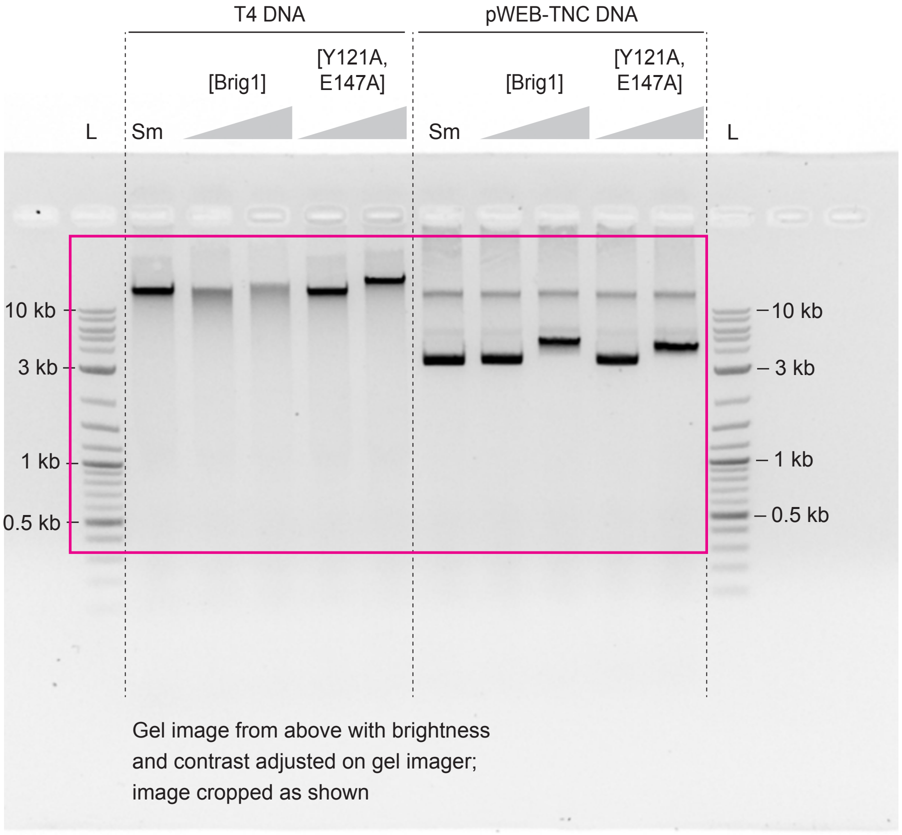


**Extended Data Fig. 7c**

wild-type *E. coli*/pAM38(*brig1*) - Arabinose

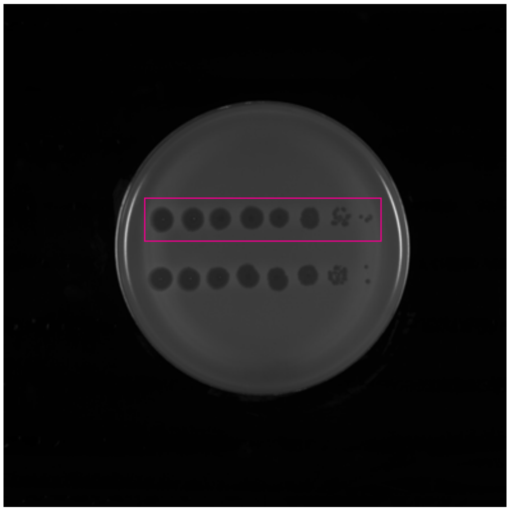

wild-type *E. coli*/pAM38(*brig1*) + Arabinose

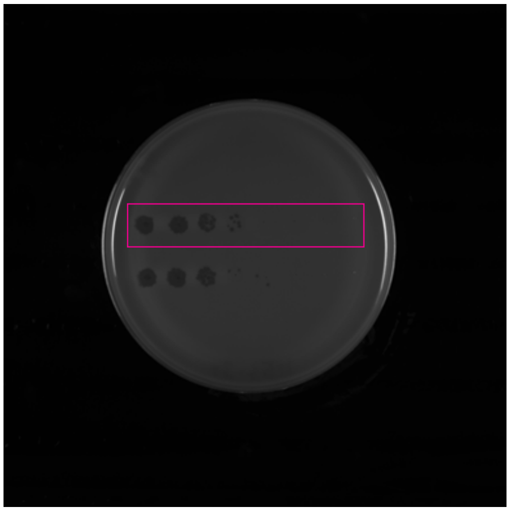

*E. coli* Δ*xthA*/pAM38(*brig1*) - Arabinose

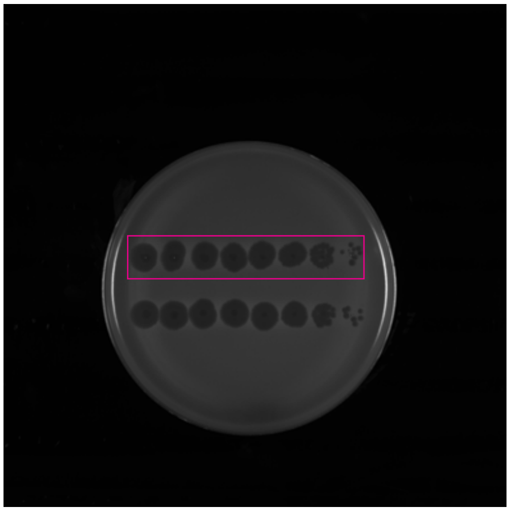

*E. coli* Δ*xthA*/pAM38(*brig1*) + Arabinose

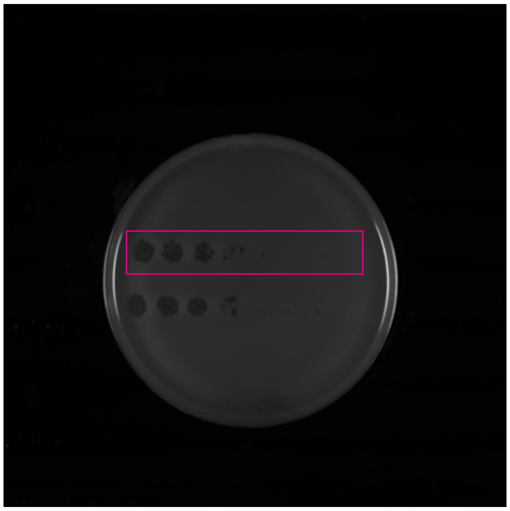

*E. coli* Δ*nfo*/pAM38(*brig1*) - Arabinose

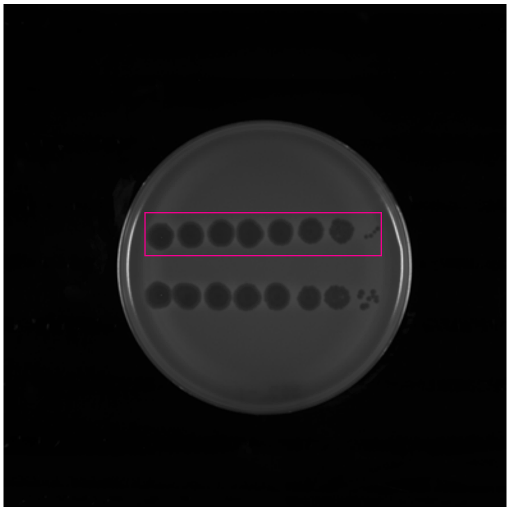

*E. coli* Δ*nfo*/pAM38(*brig1*) + Arabinose

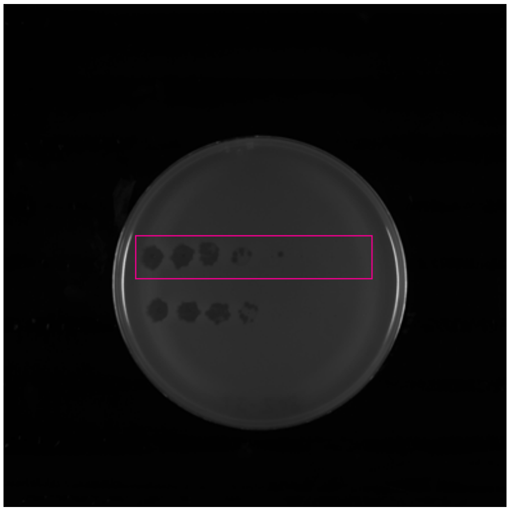

These are plate images for the representative images shown in Extended Data Fig 8a.
Images were cropped as shown and then auto contrasted on Adobe Photoshop.
Continued on next page.

# Extended Data Fig. 8a

Phage T4

*E. coli ΔxthA Δnfo*/pAM38(*brig1*) - Arabinose

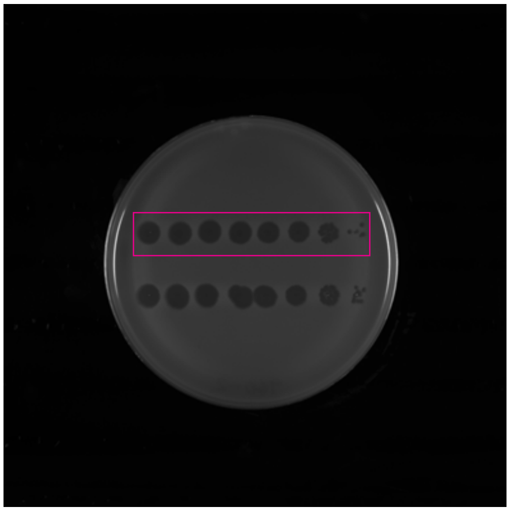

*E. coli ΔxthA Δnfo*/pAM38(*brig1*) + Arabinose

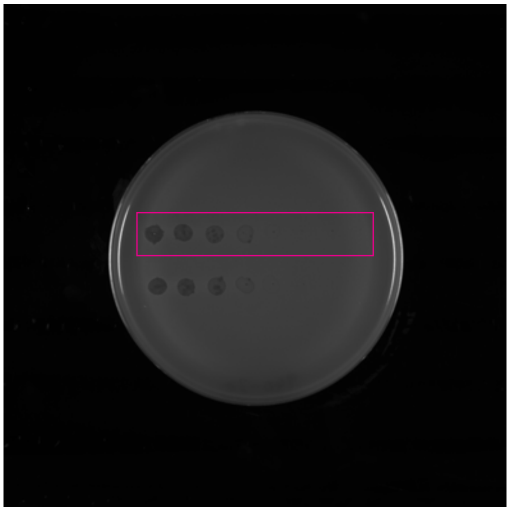

*E. coli Δnth*/pAM38(*brig1*) - Arabinose

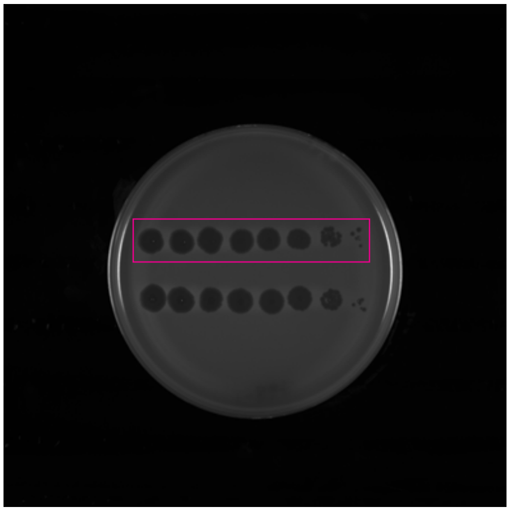

*E. coli Δnth*/pAM38(*brig1*) + Arabinose

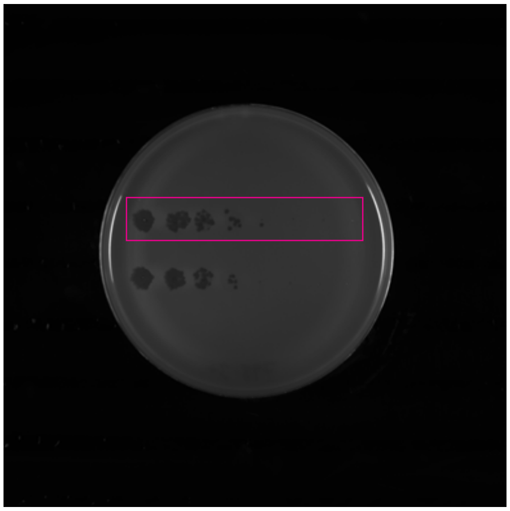

*E. coli ΔyedK*/pAM38(*brig1*) - Arabinose

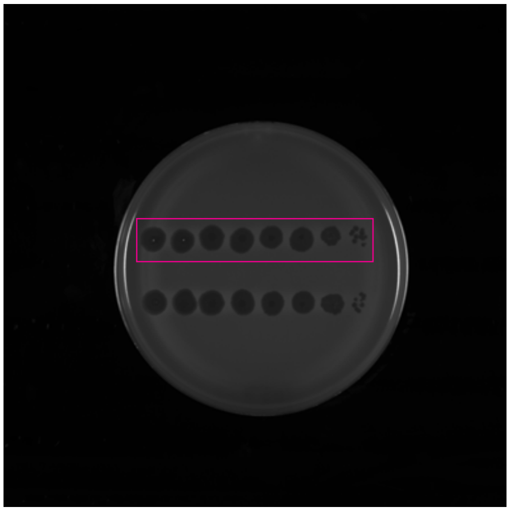

*E. coli ΔyedK*/pAM38(*brig1*) + Arabinose

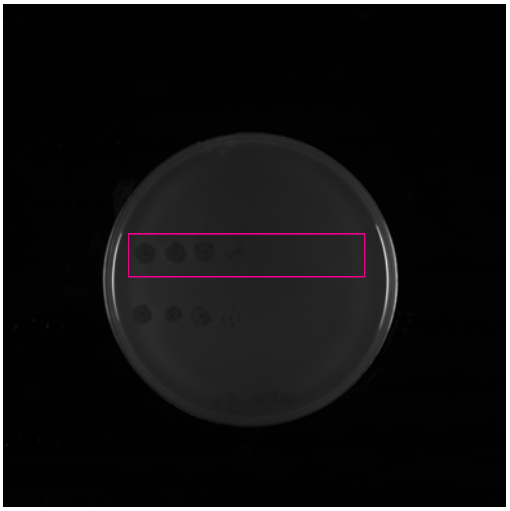

These are plate images for the representative images shown in Extended Data Fig 8a.
Images were cropped as shown and then auto contrasted on Adobe Photoshop.
Continued from previous page.

**Extended Data Fig. 8a**

pWEB-TNC + no plasmid - Arabinose

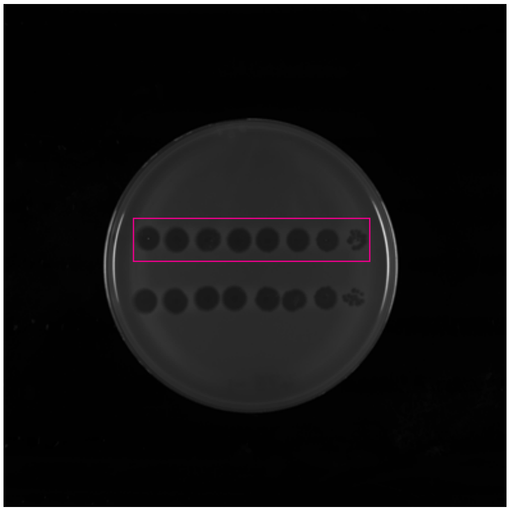

pWEB-TNC + no plasmid + Arabinose

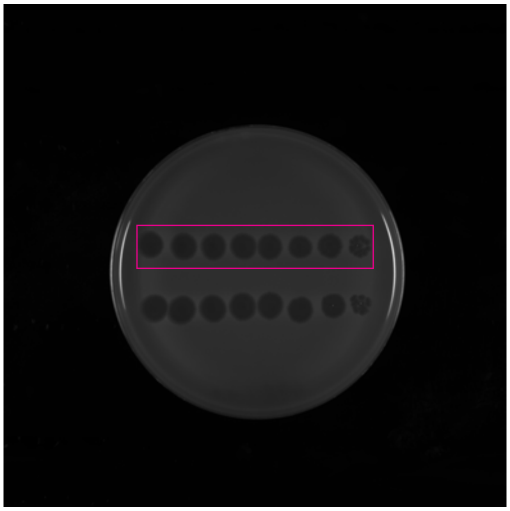

pWEB-TNC + pAM38(*xthA*) - Arabinose

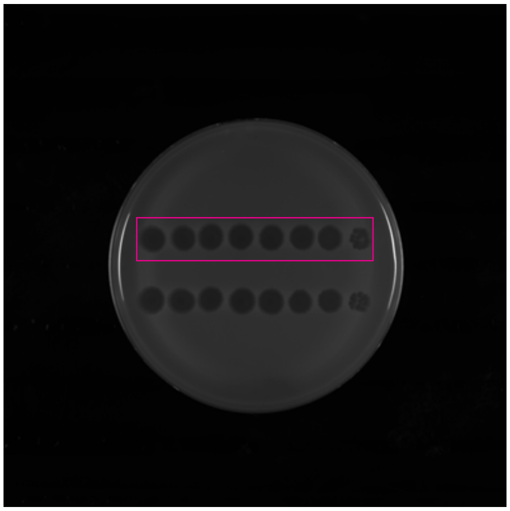

pWEB-TNC + pAM38(*xthA*) + Arabinose

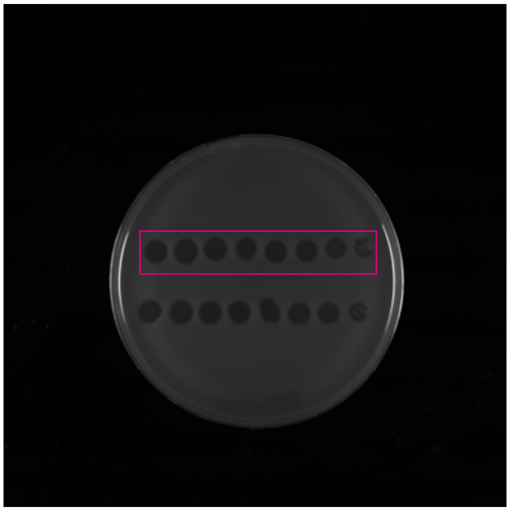

pWEB-TNC + pAM38(*nfo*) - Arabinose

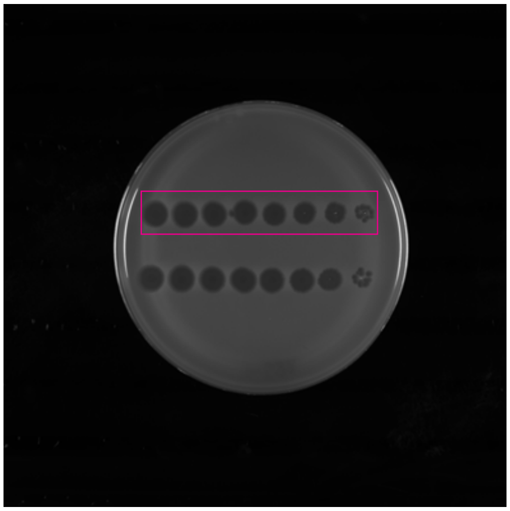

pWEB-TNC + pAM38(*nfo*) + Arabinose

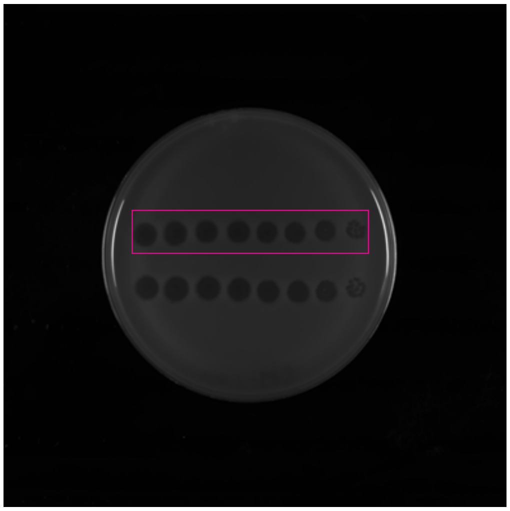

These are plate images for the representative images shown in Extended Data Fig 8b.
Images were cropped as shown and then auto contrasted on Adobe Photoshop.
Continued on next page.

# Extended Data Fig. 8b

Phage T4

pBrig1 + no plasmid - Arabinose

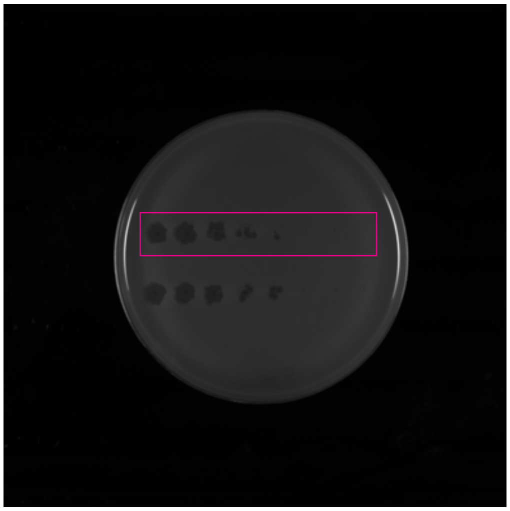

pBrig1 + no plasmid + Arabinose

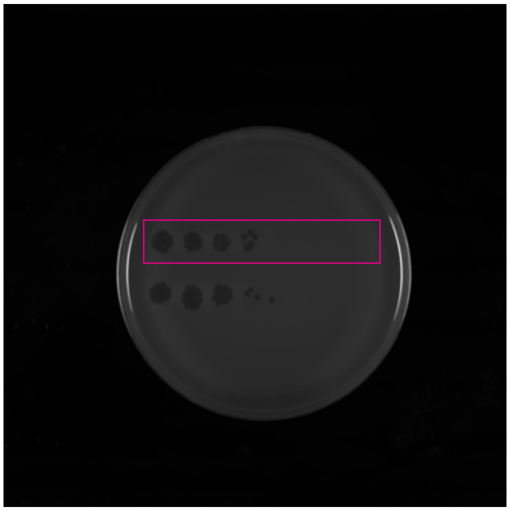

pBrig1 + pAM38(*xthA*) - Arabinose

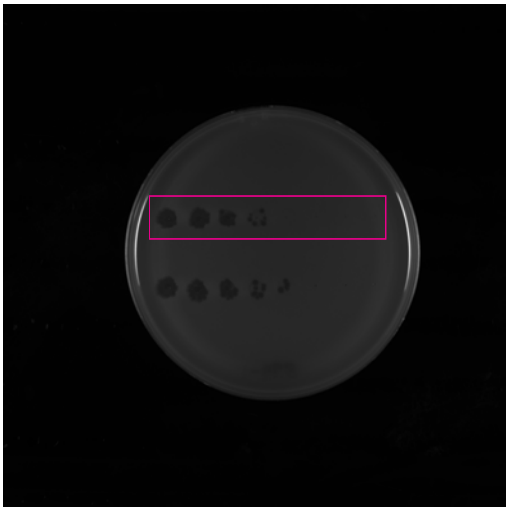

pBrig1 + pAM38(*xthA*) + Arabinose

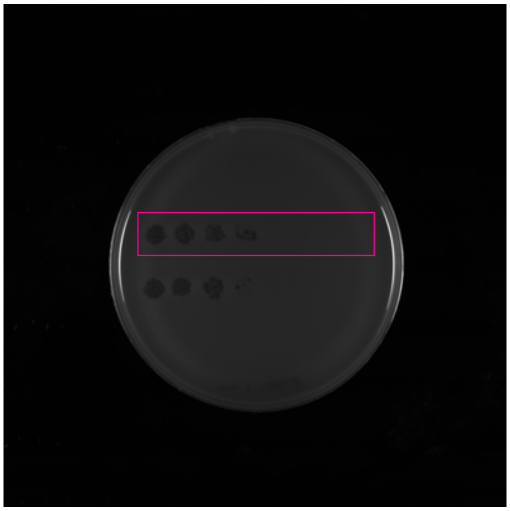

pBrig1 + pAM38(*nfo*) - Arabinose

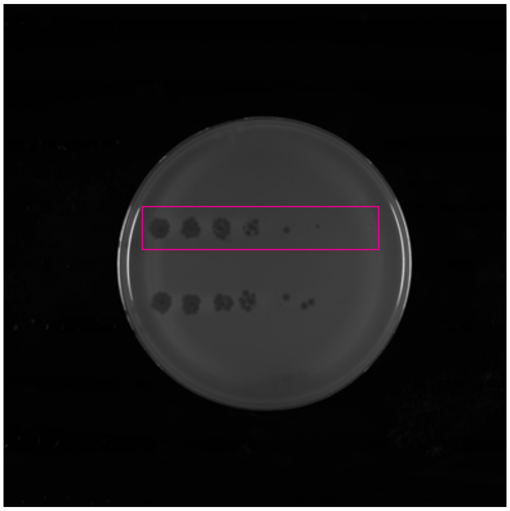

pBrig1 + pAM38(*nfo*) + Arabinose

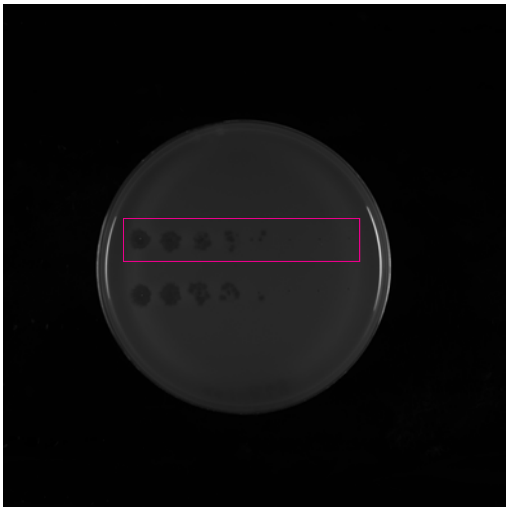

These are plate images for the representative images shown in Extended Data Fig 8b.
Images were cropped as shown and then auto contrasted on Adobe Photoshop.
Continued from previous page.

# Extended Data Fig. 8b

Phage T4

wild-type *E. coli*/pAM38(*brig1*) - Arabinose

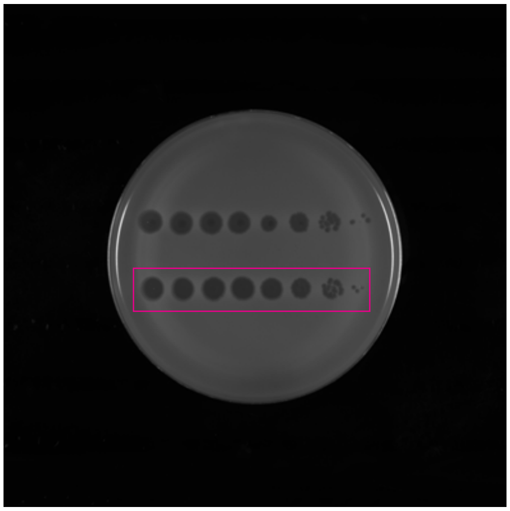

wild-type *E. coli*/pAM38(*brig1*) + Arabinose

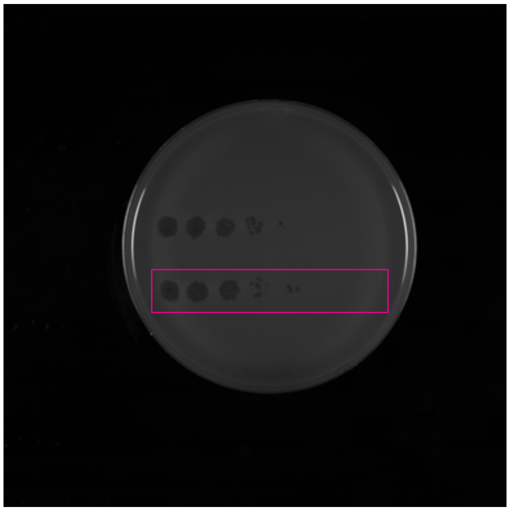

*E. coli* Δ*recB*/pAM38(*brig1*) - Arabinose

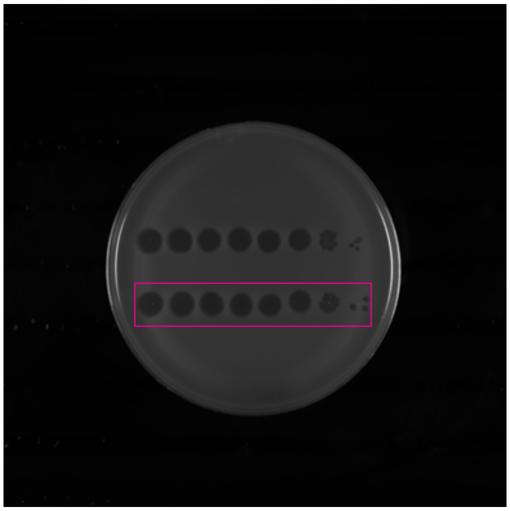

*E. coli* Δ*recB*/pAM38(*brig1*) + Arabinose

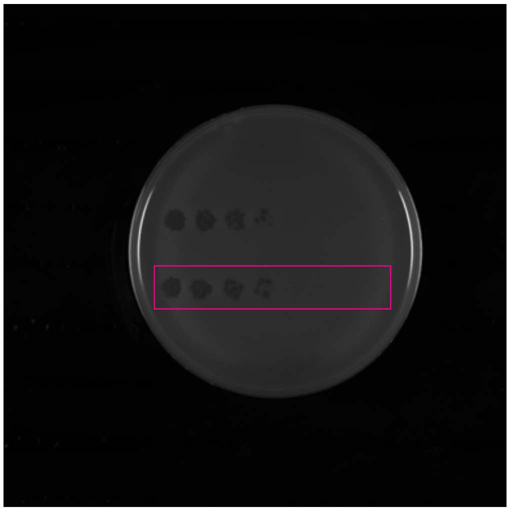

*E. coli* Δ*recC*/pAM38(*brig1*) - Arabinose

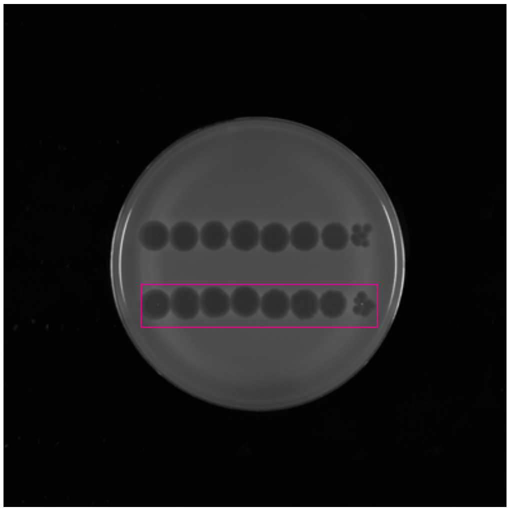

*E. coli* Δ*recC*/pAM38(*brig1*) + Arabinose

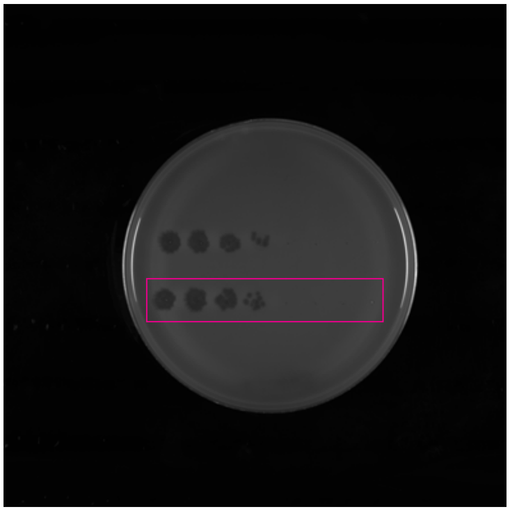

These are plate images for the representative images shown in Extended Data Fig 8c.
Images were cropped as shown and then auto contrasted on Adobe Photoshop.
Continued on next page.

**Extended Data Fig. 8c**

*E. coli* Δ*recD*/pAM38(*brig1*) - Arabinose

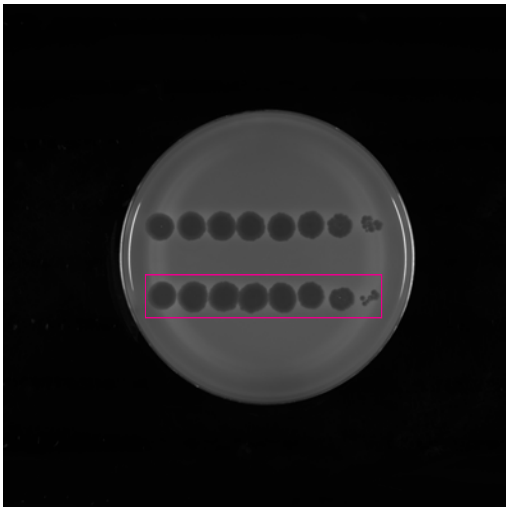

*E. coli* Δ*recD*/pAM38(*brig1*) + Arabinose

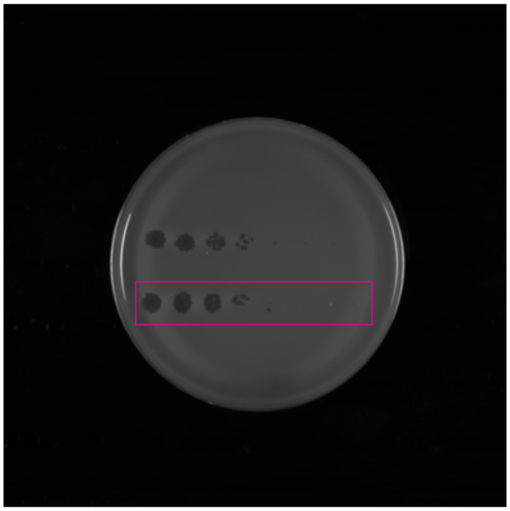

*E. coli* Δ*recQ*/pAM38(*brig1*) - Arabinose

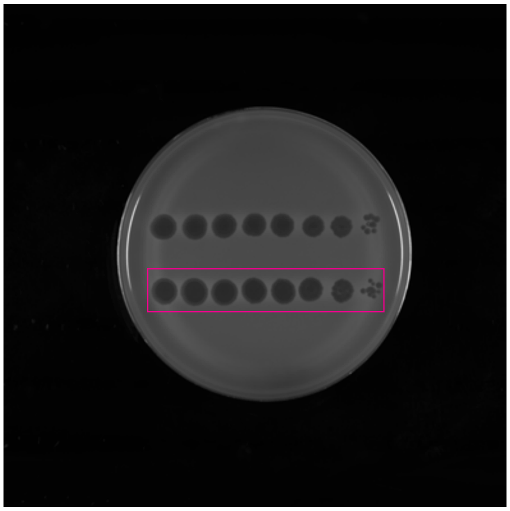

*E. coli* Δ*recQ*/pAM38(*brig1*) + Arabinose

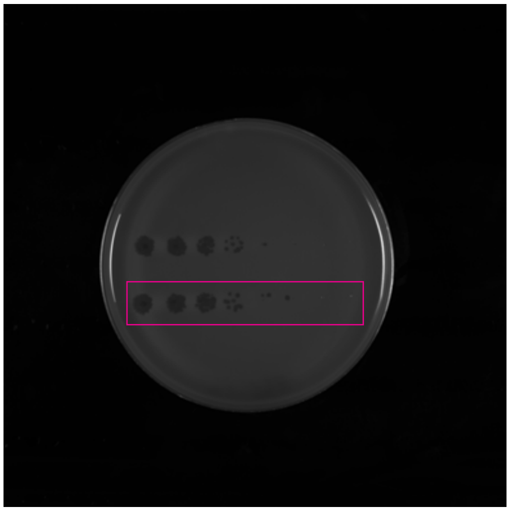

*E. coli* Δ*recJ*/pAM38(*brig1*) - Arabinose

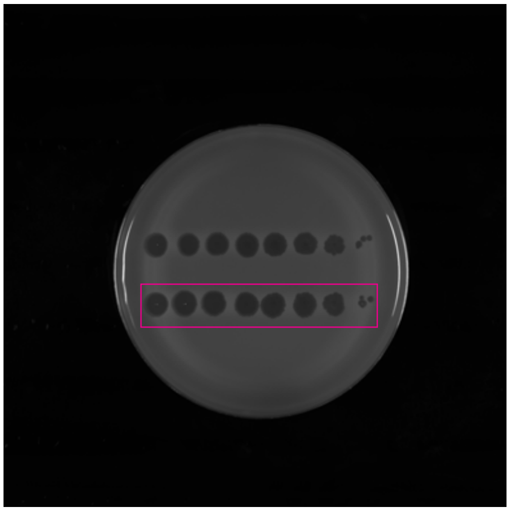

*E. coli* Δ*recJ*/pAM38(*brig1*) + Arabinose

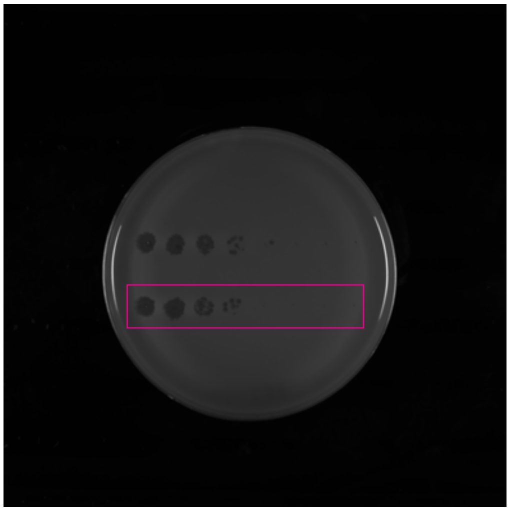

These are plate images for the representative images shown in Extended Data Fig 8c.
Images were cropped as shown and then auto contrasted on Adobe Photoshop.
Continued from previous page.

# Extended Data Fig. 8c

Phage T4

*E. coli* Δ*recA*/pAM38(*brig1*) - Arabinose

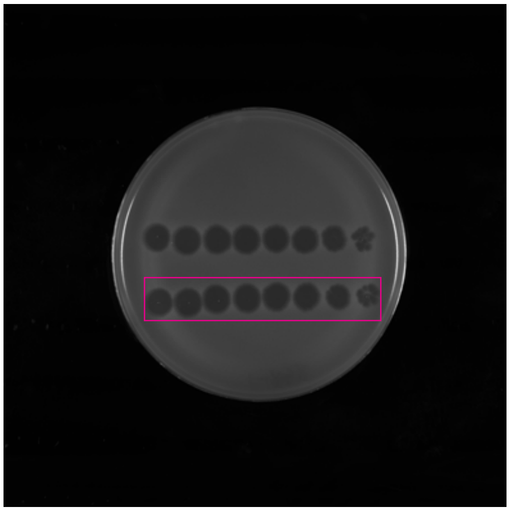

*E. coli* Δ*recA*/pAM38(*brig1*) + Arabinose

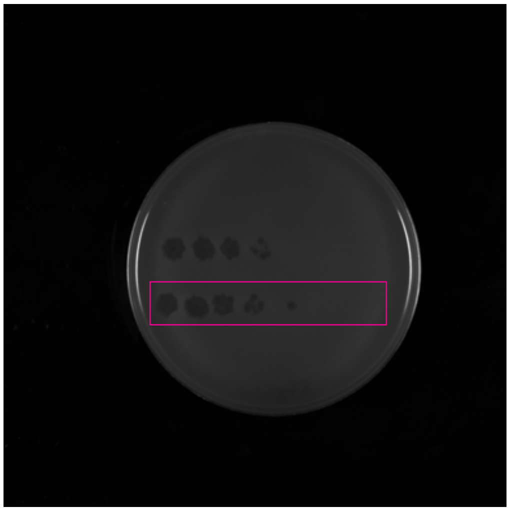

These are plate images for the representative images shown in Extended Data Fig 8c.
Images were cropped as shown and then auto contrasted on Adobe Photoshop.
Continued from previous page.

# Extended Data Fig. 8c

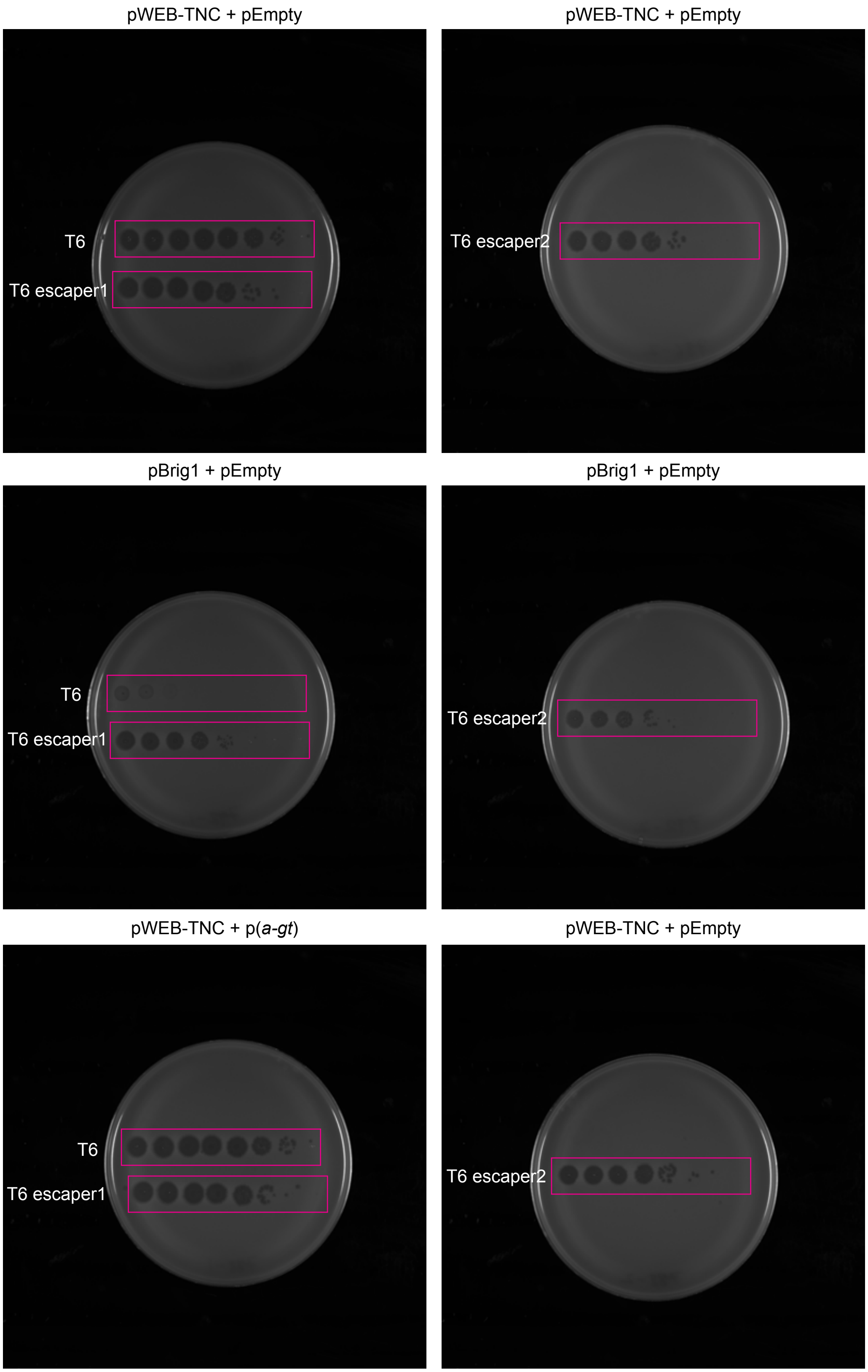


Continued on next page.
These are plate images for the representative images shown in Extended Data Fig 9b. Images were cropped as shown and then auto contrasted on Adobe Photoshop; the pBrig1 + pEmpty row for phage T6 was further enhanced for brightness to enable better visibility.

**Extended Data Fig. 9b**

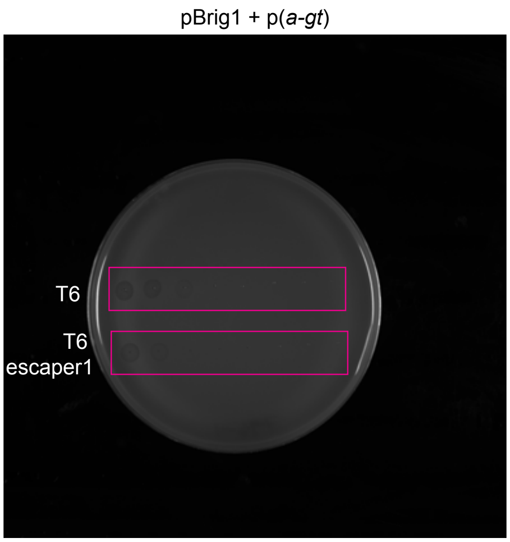


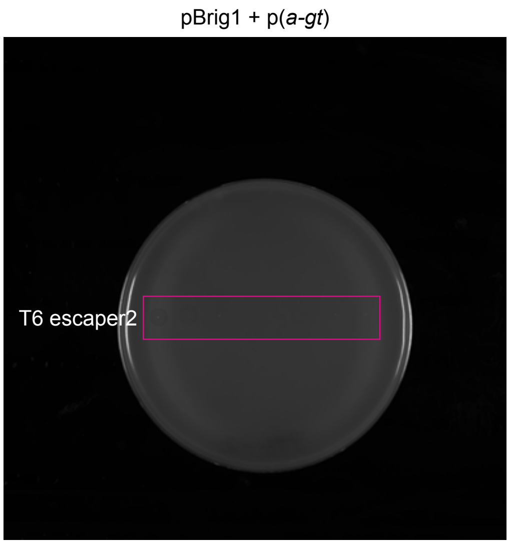


Continued from previous page.
These are plate images for the representative images shown in Extended Data Fig 9b. Images were cropped as shown and then auto contrasted on Adobe Photoshop; the pBrig1 + p(*a-gt*) rows for all phages were further enhanced for brightness to enable better visibility.

# Extended Data Fig. 9b

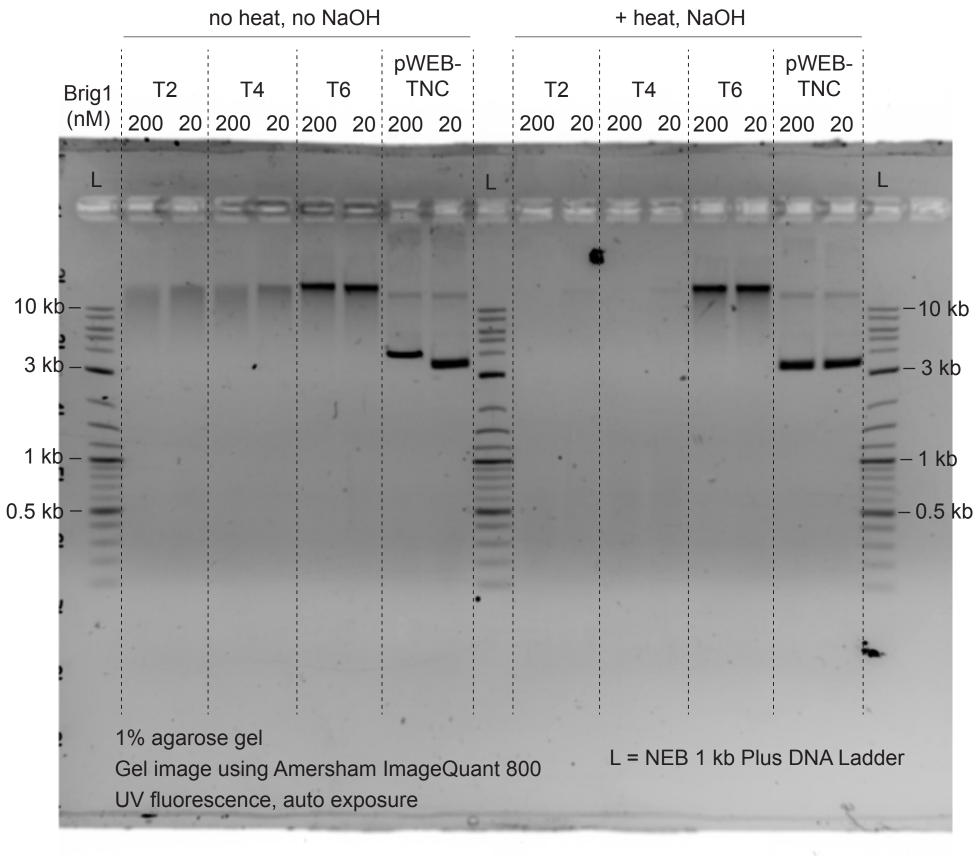


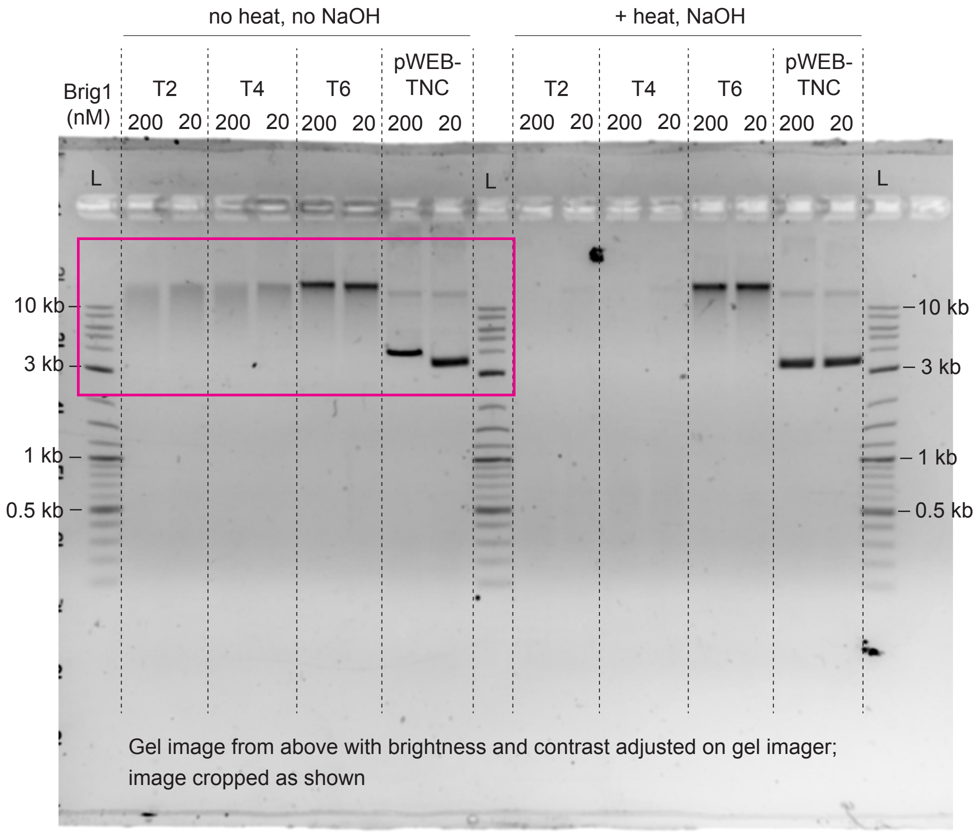


**Extended Data Fig. 9c**

pWEB-TNC Replicate 1

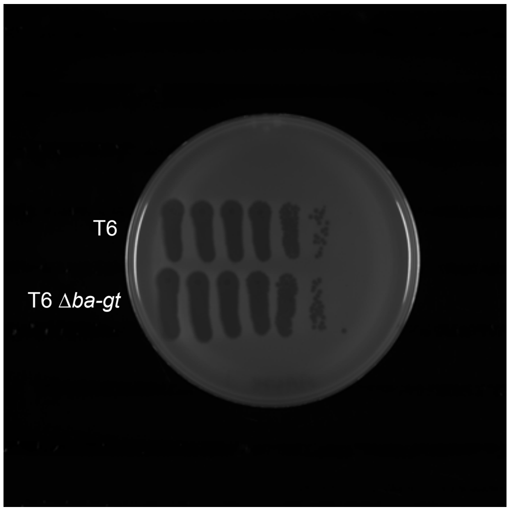


pBrig1 whole plate plaque counting
Phage T6 (~1 x $10^{8}$ PFUs added)
Replicate 1

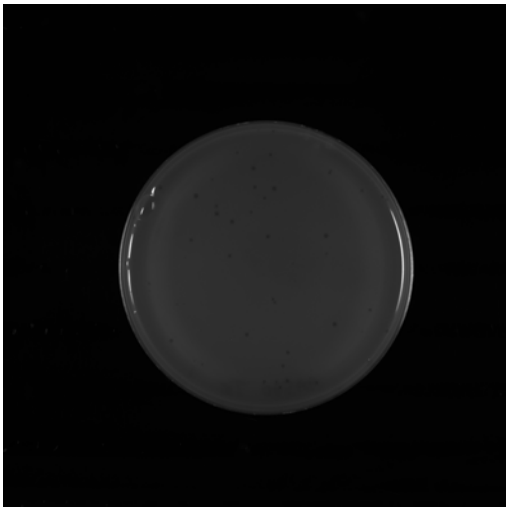

pBrig1 whole plate plaque counting
Phage T6 Δ*ba-gt* (~1 x $10^{8}$ PFUs added)
Replicate 1

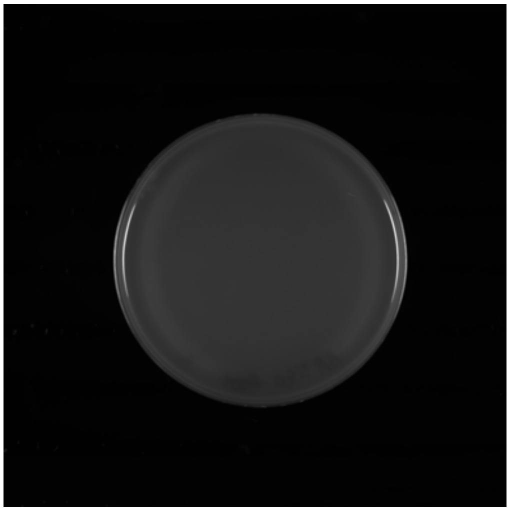

pWEB-TNC Replicate 2

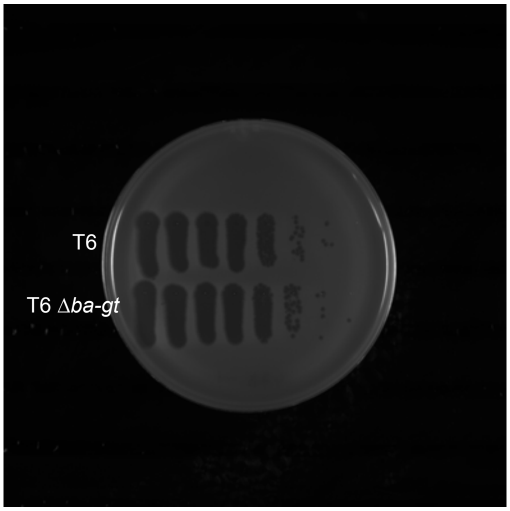


pBrig1 whole plate plaque counting
Phage T6 (~1 x $10^{8}$ PFUs added)
Replicate 2

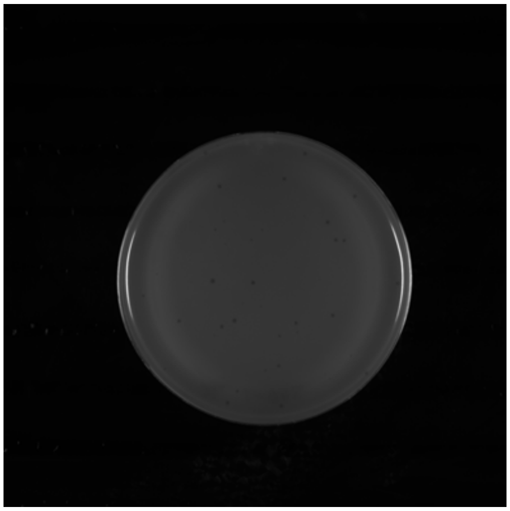

pBrig1 whole plate plaque counting
Phage T6 Δ*ba-gt* (~1 x $10^{8}$ PFUs added)
Replicate 2

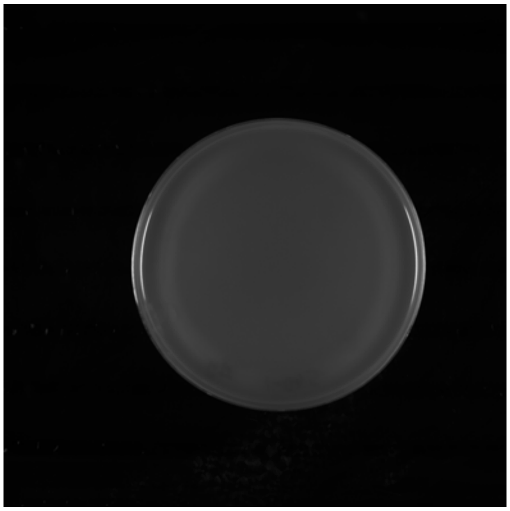

pWEB-TNC Replicate 3

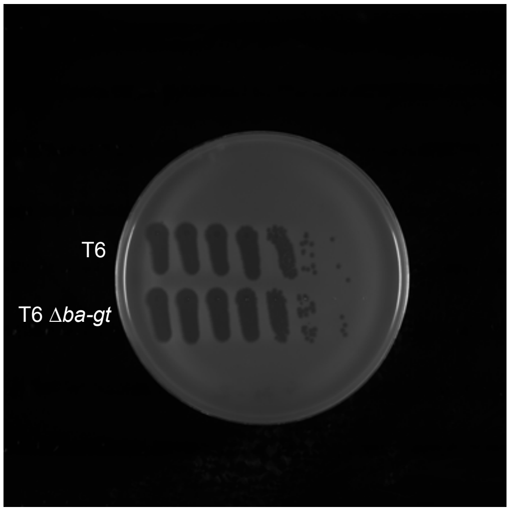


pBrig1 whole plate plaque counting
Phage T6 (~1 x $10^{8}$ PFUs added)
Replicate 3

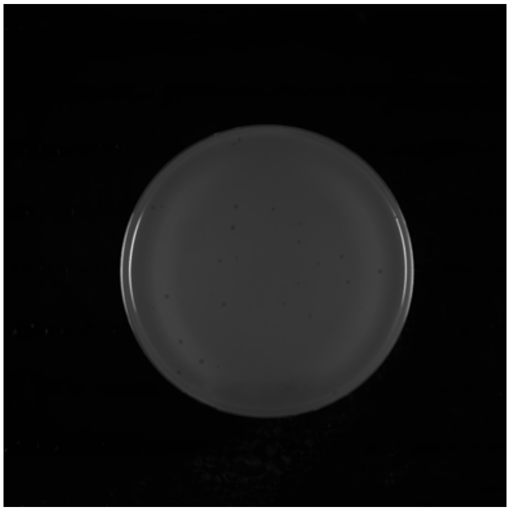

pBrig1 whole plate plaque counting
Phage T6 Δ*ba-gt* (~1 x $10^{8}$ PFUs added)
Replicate 3

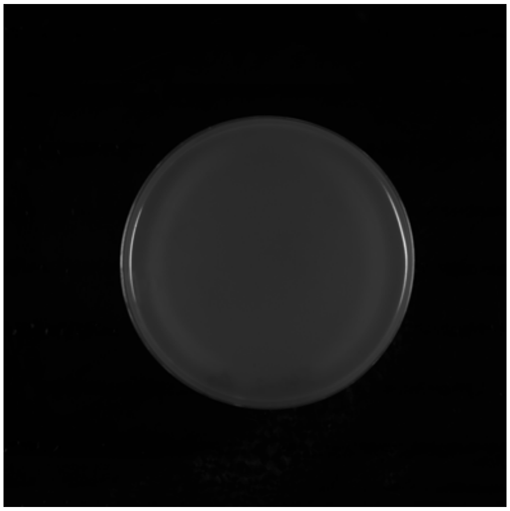

These are plate images for the quantification shown in Extended Data Fig 9d. Plaques were quantified using the counting tool in FIJI (ImageJ) and plaques on pBrig1 lawns were normalized to total plaques formed on pWEB-TNC lawns to calculate efficiency of plaquing.

**Extended Data Fig. 9d**

pWEB-TNC

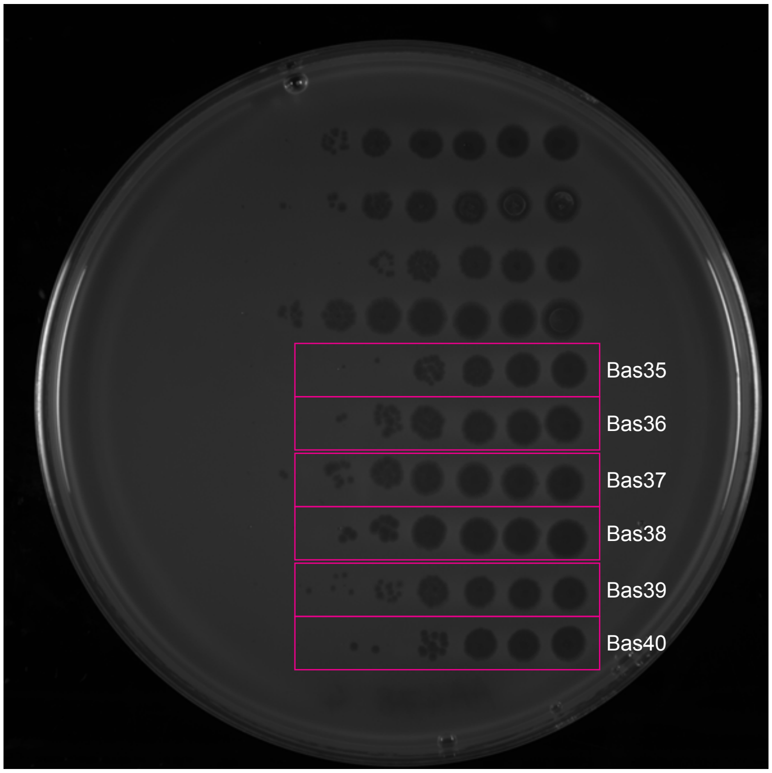


pBrig1

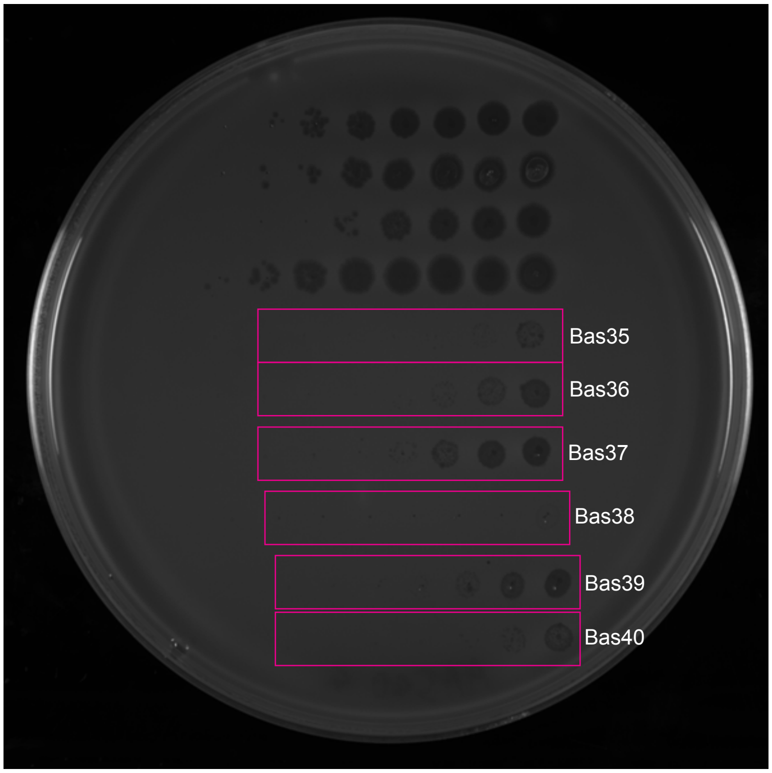


pWEB-TNC

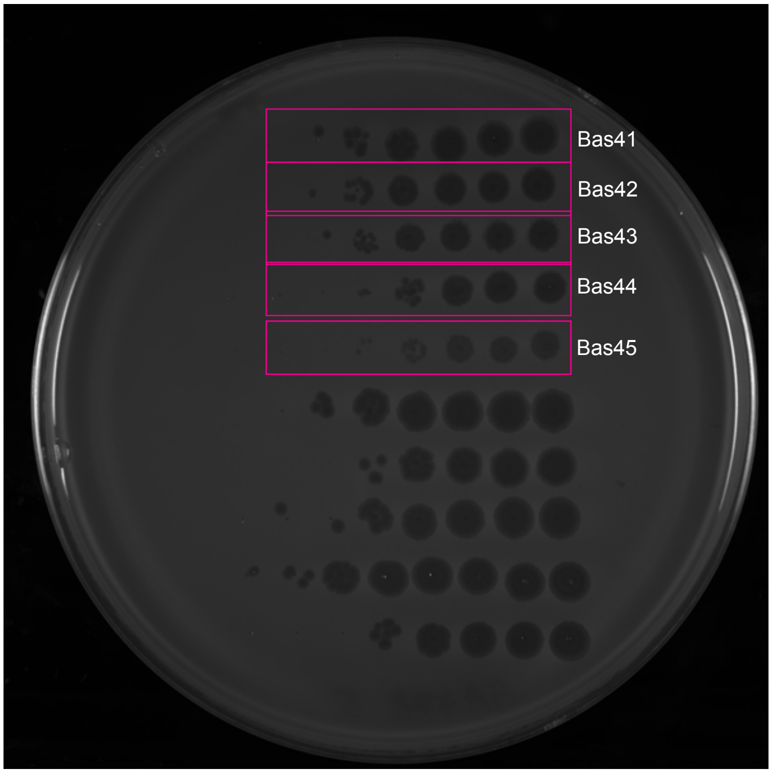


pBrig1

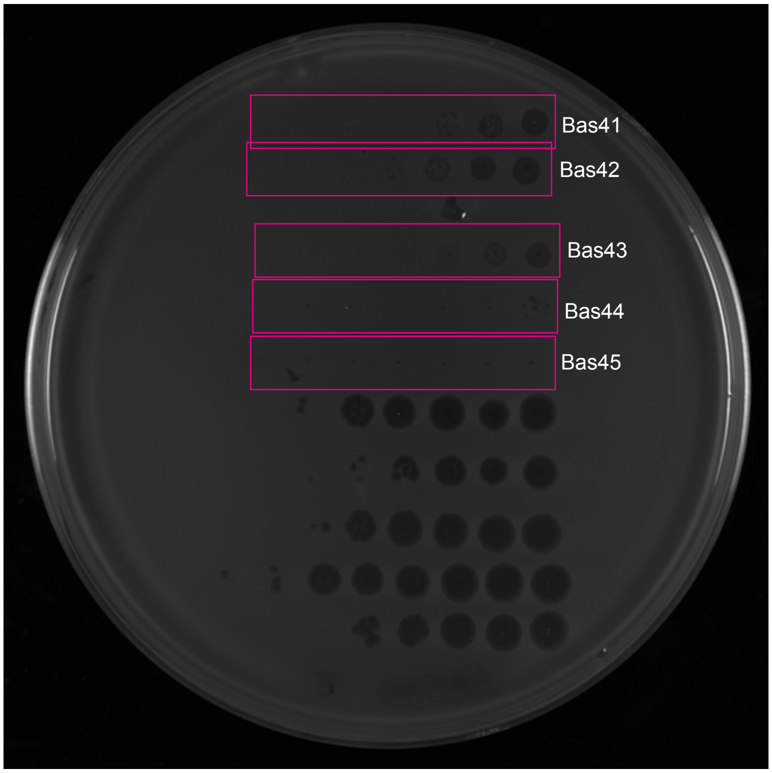


These are plate images for the representative images shown in Extended Data Fig 9e. Images were cropped as shown and then auto contrasted on Adobe Photoshop. Image reflected vertically in main figure. Continued on next page.

**Extended Data Fig. 9e**

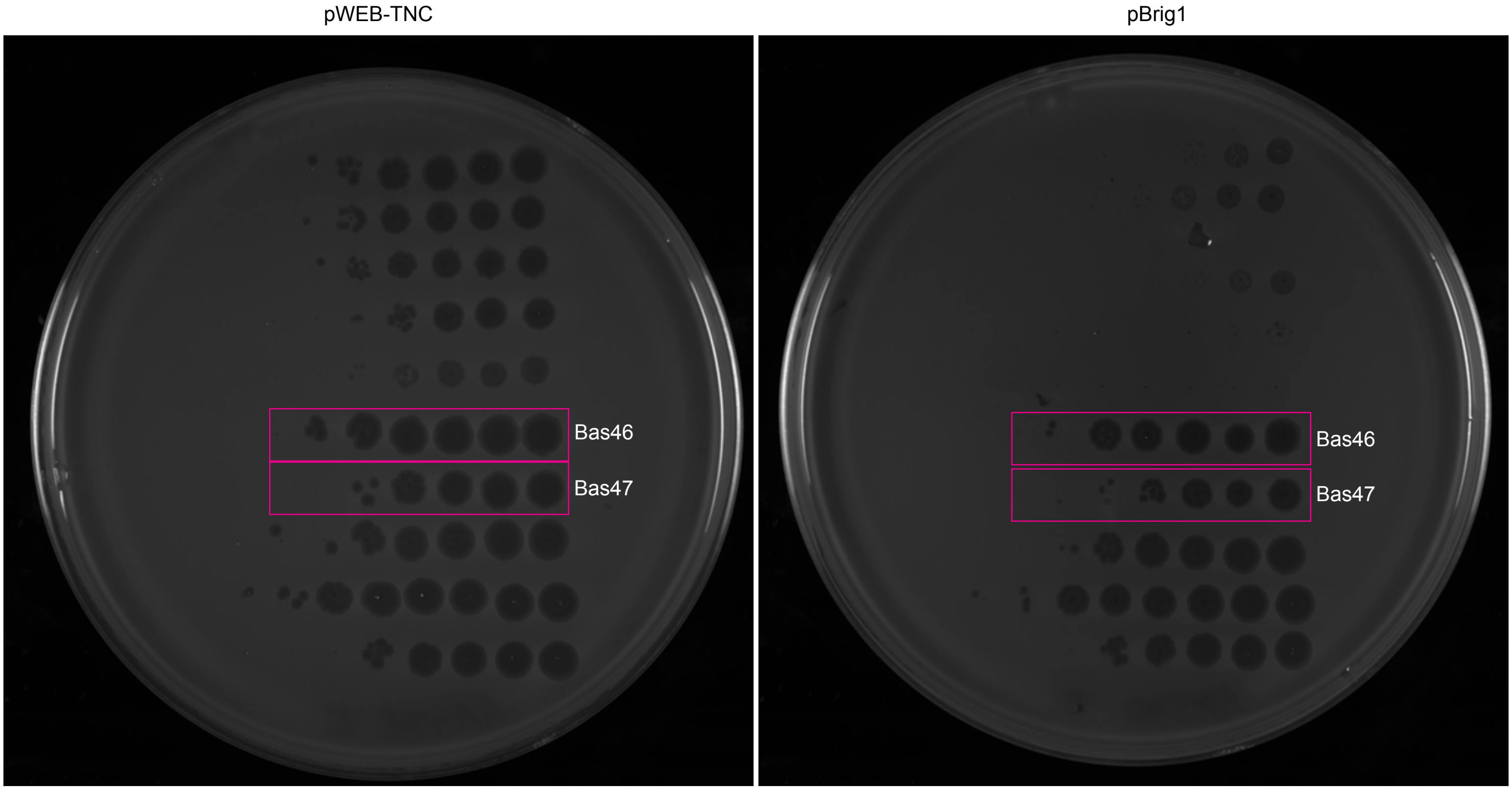


Same images as bottom row on previous page. These are plate images for the representative images shown in Extended Data Fig 9e. Images were cropped as shown and then auto contrasted on Adobe Photoshop. Image reflected vertically in main figure. Continued from previous page.

**Extended Data Fig. 9e**

**SUPPLEMENTARY FIGURE 2**

# Replicates for Fig. 1a

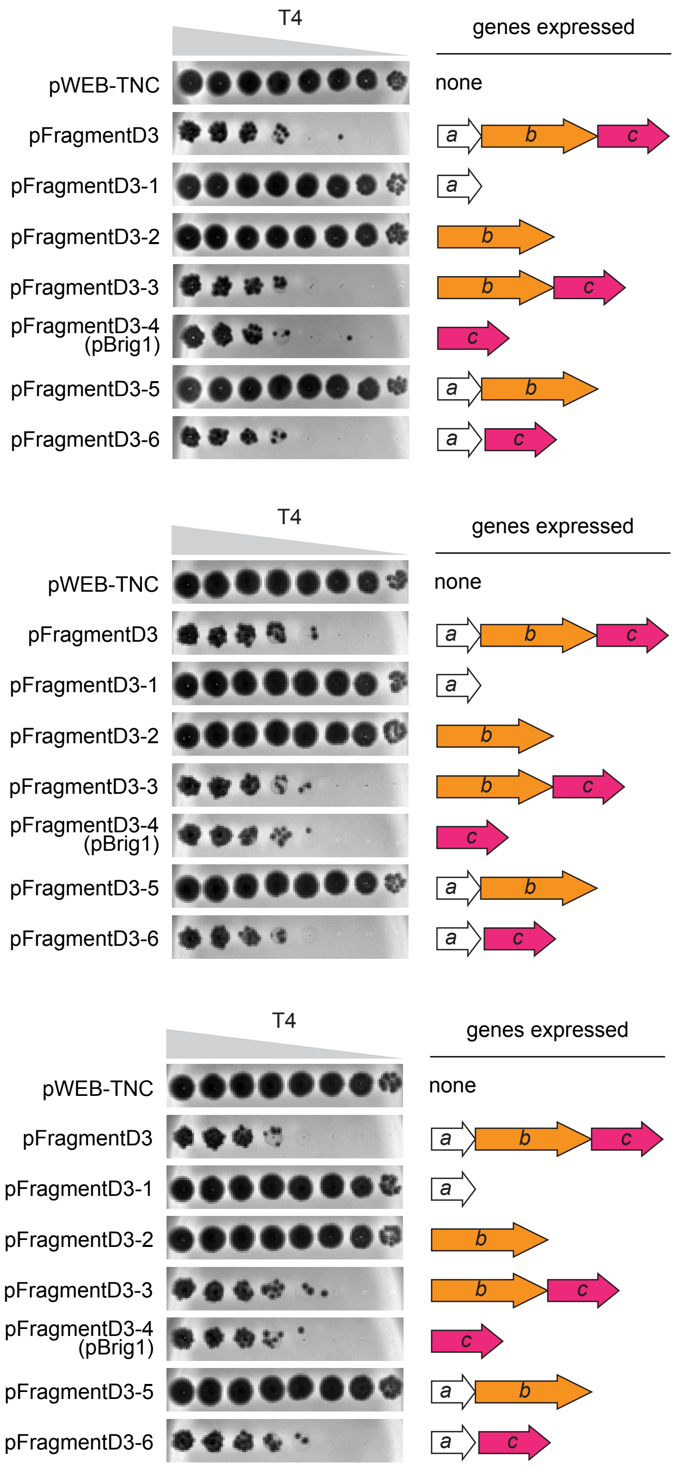

# Replicates for Fig. 2a

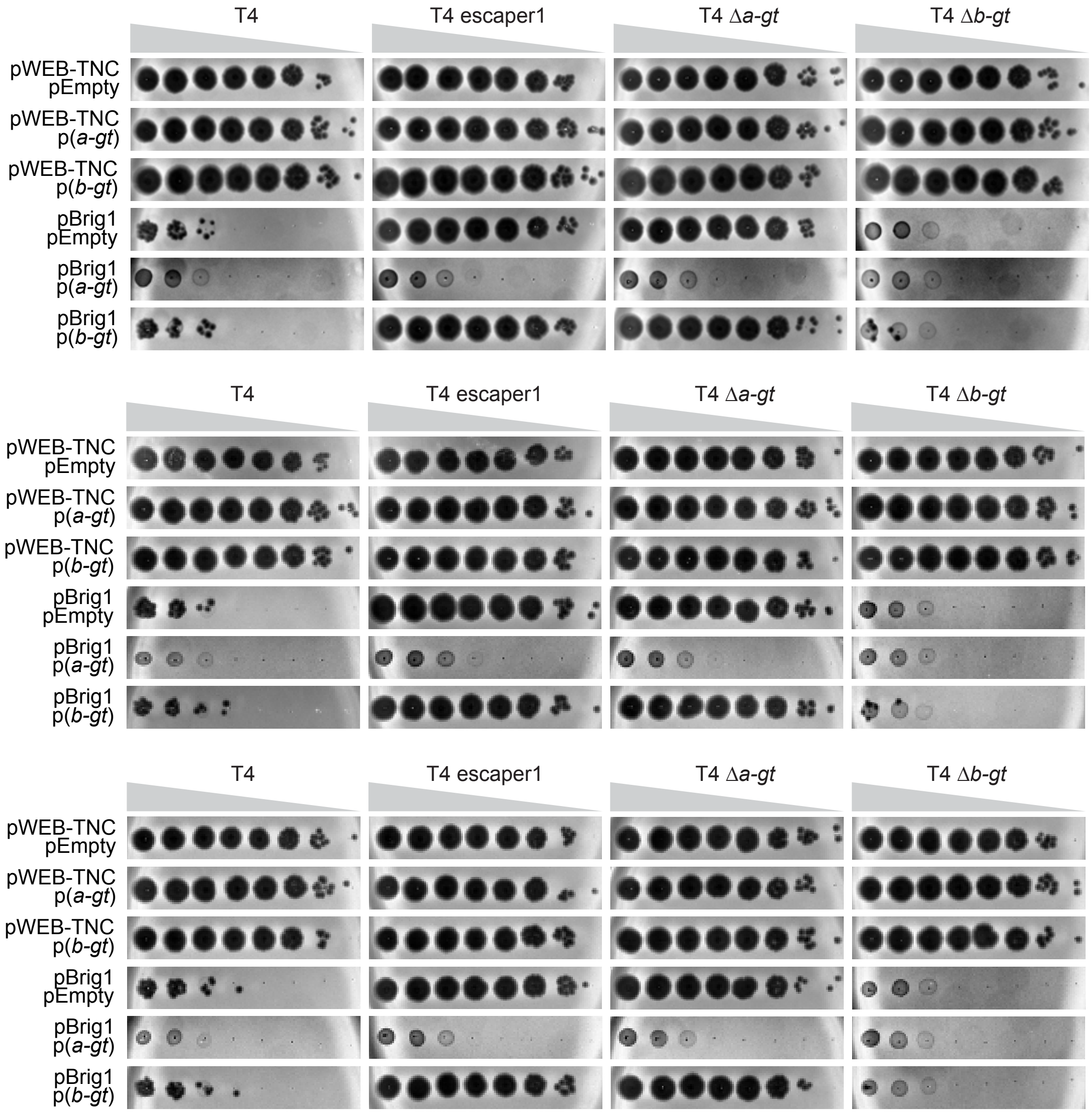

# Replicates for Fig. 2c

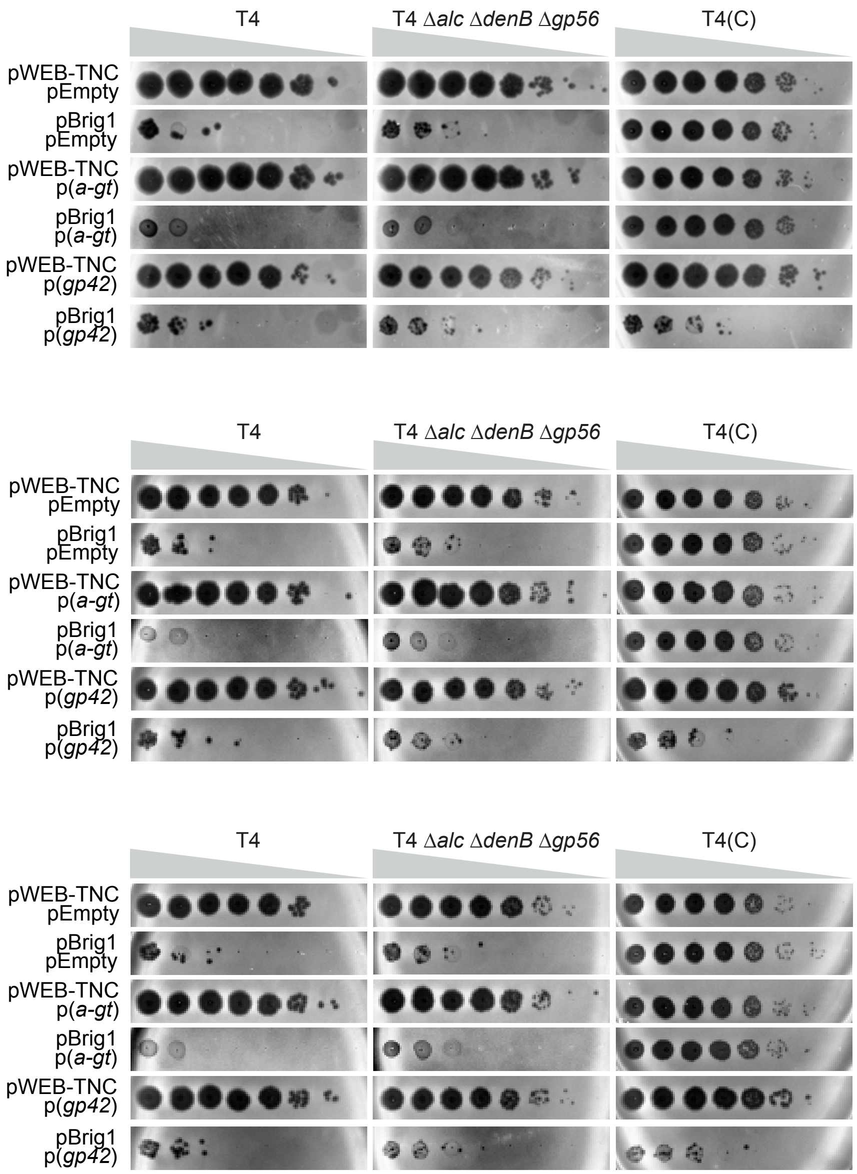

# Replicates for Fig. 4a

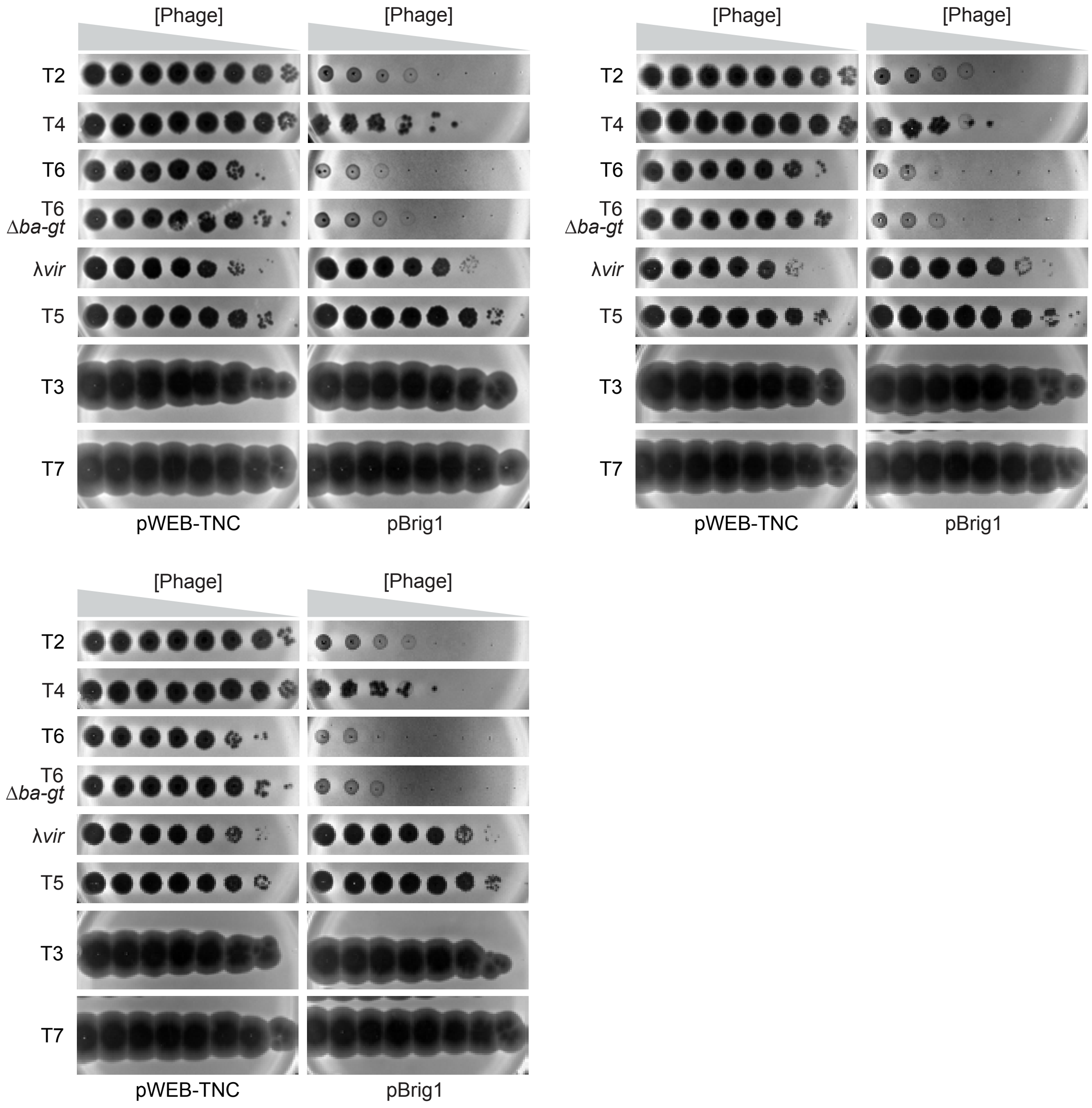

# Replicates for Fig. 5b

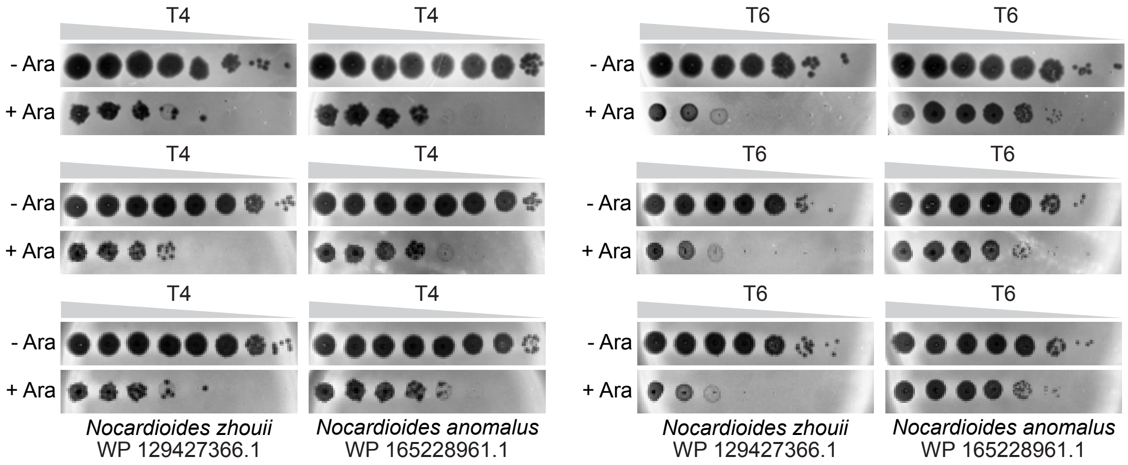

# Replicates for Extended Data Fig. 1d

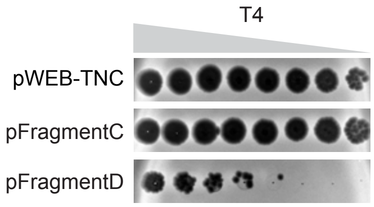


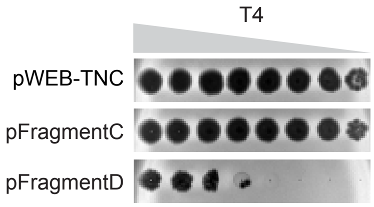

# Replicates for Extended Data Fig. 1f

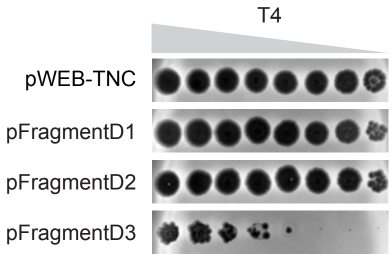


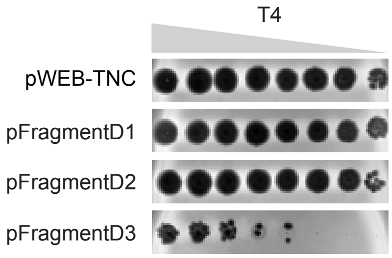

# Replicates for Extended Data Fig. 1g

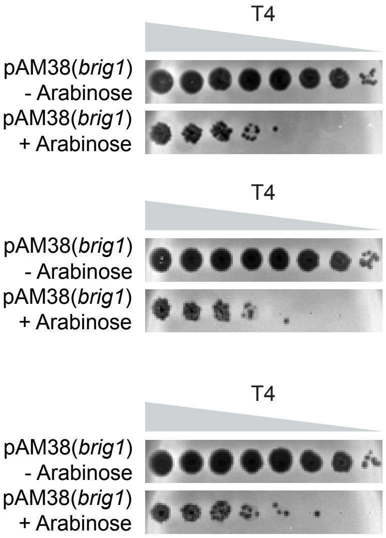

# Replicates for Extended Data Fig. 7a

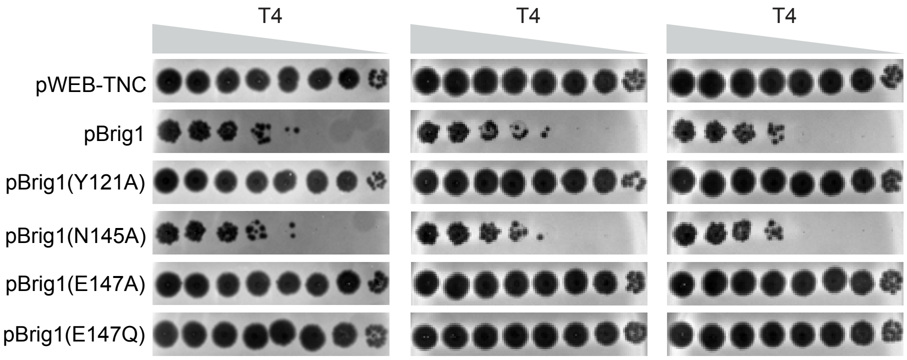

# Replicates for Extended Data Fig. 8a

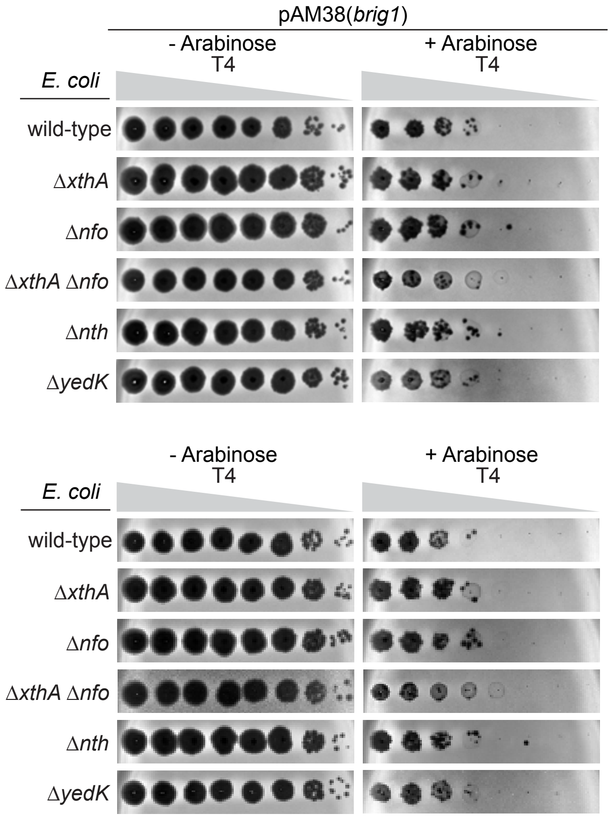

# Replicates for Extended Data Fig. 8b

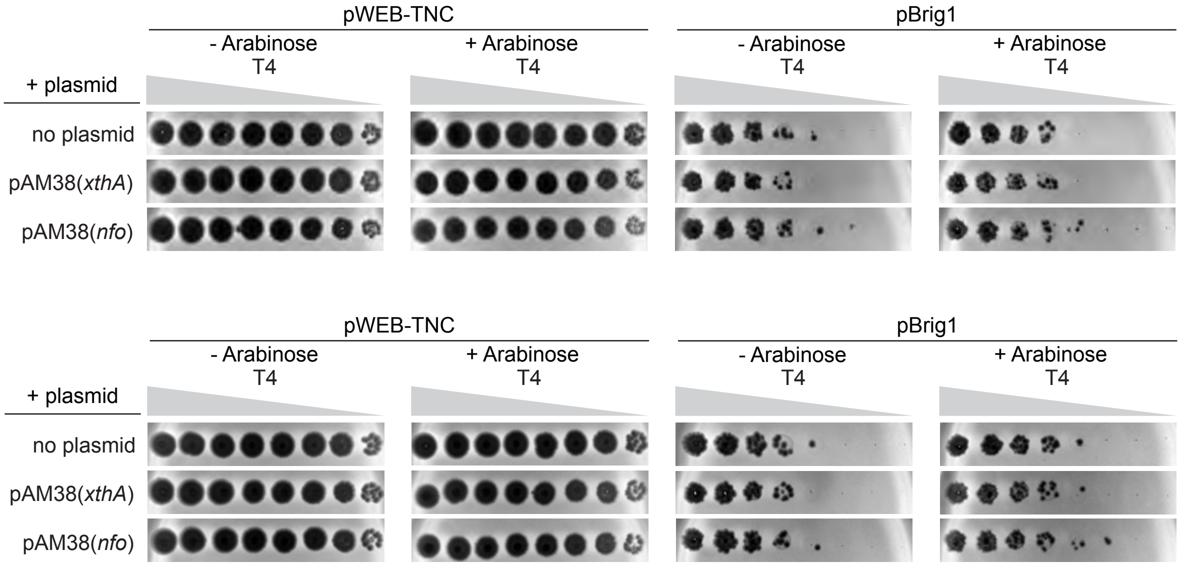

# Replicates for Extended Data Fig. 8c

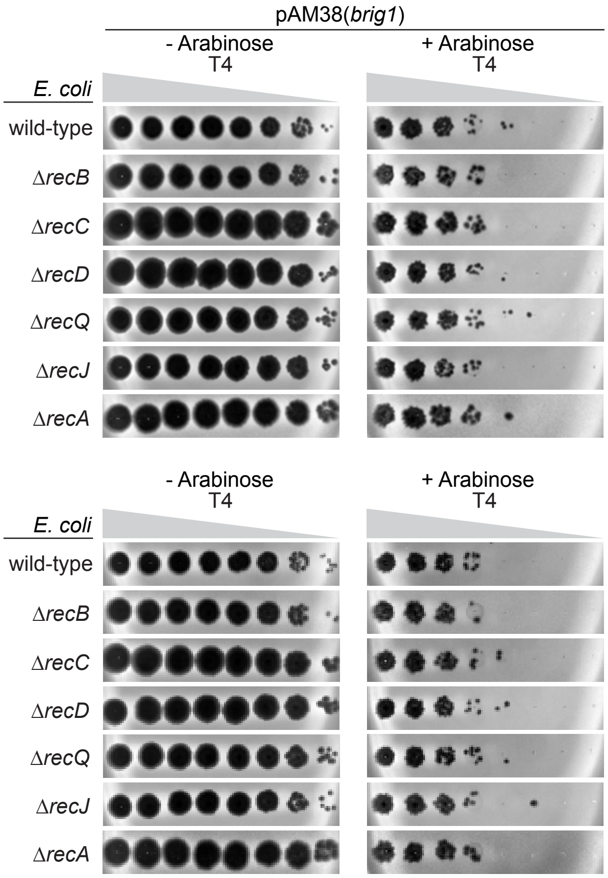

# Replicates for Extended Data Fig. 9b

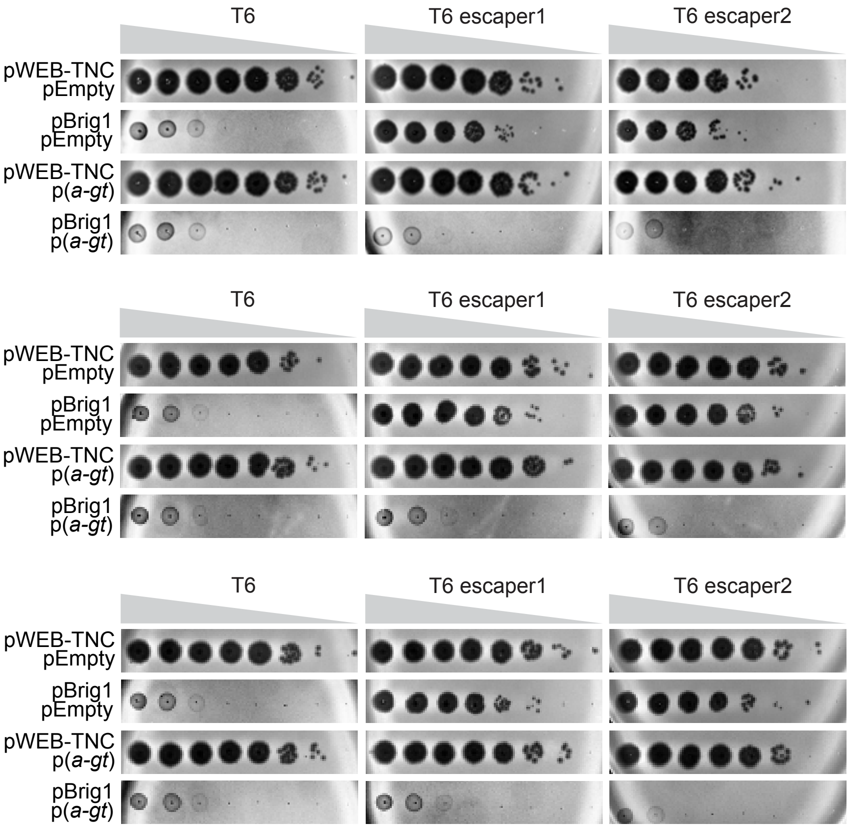

# Replicates for Extended Data Fig. 9e

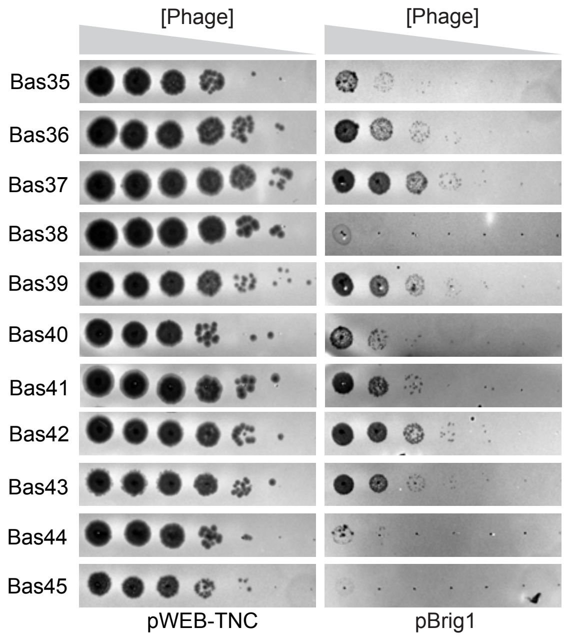


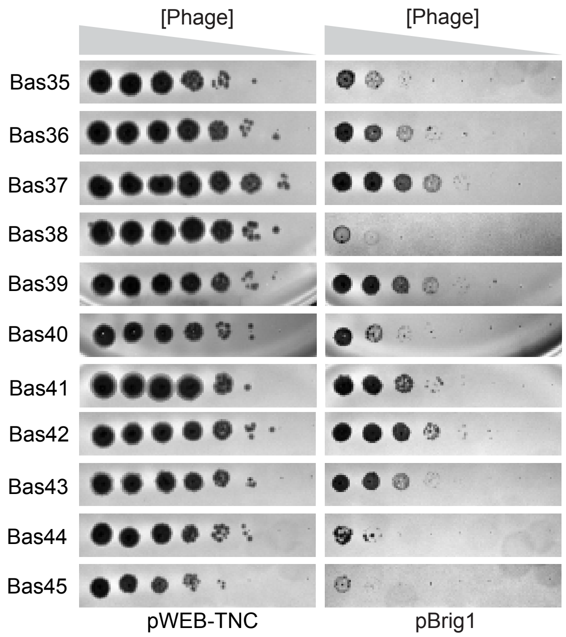


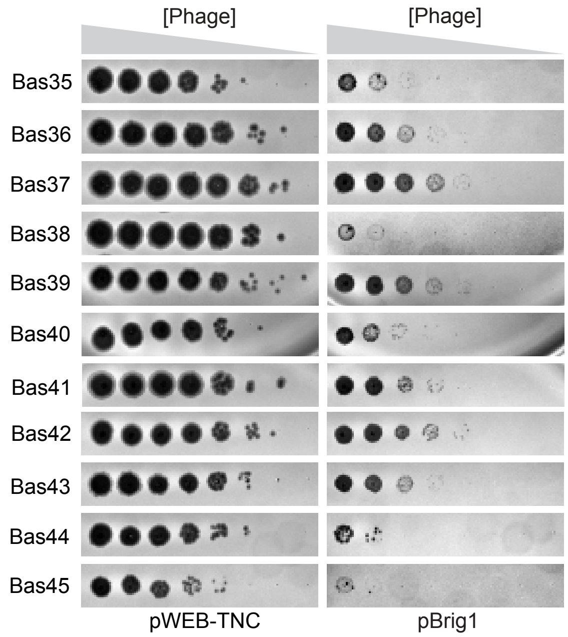

# Replicates for Extended Data Fig. 9e (continued)

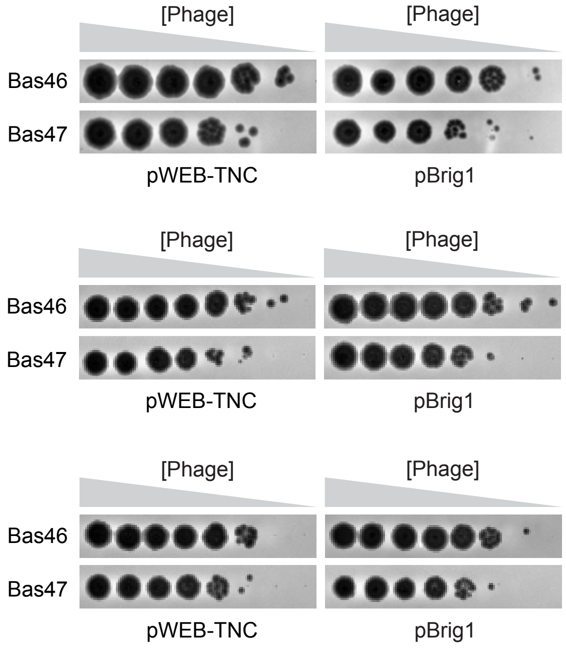

## SUPPLEMENTARY SEQUENCES

>brig1
ATGAGTGCACGCGAACGGGTGGCTGACCTCTGGGACGAGGTCGTCGCGAACTGGCAGCCCGGCC
AGGACGTCTGGCCGGGTCCCCTCGATGACTGGTTTAAGAGTTATCGAGGCCGCGGCTCGGGTGC
CGTCGACCTGGATCAATACCCCGACCCGTGGGTGGGAGATCTCCGCGGCCGCGTCCGCGAACCC
CGGCTGGTGGTCCTCGGACTCAACCCCGGGATTGGCTACGAGGAACTCCAGGGGCCCAACGGGA
CCTGGACGAAGAGGATCCTGGACACGGGCTACAGCCACTGCCTCCACCGCAGCCCACCCGAGGA
CCCTGCAGGCTGGATTCCCGTCCACAAGAGGCCGAGCGCCTACTGGGTGCGGGTCGAGCACTTT
GCCCGGCGCTGGTTGGGTGACACATCCGCCGGCCATCGGGACATTCTGAACTTCGAGCTCTACC
CTTGGCACTCGCCCAAGCTGACGGGTGGGCTCGCCTCGCCTCCCGCGATCGTTCGCGAGTTCGT
GCTGGATCCGGTCGCTGAGGTCGGCGTCCCGCAGGTTTTCGCCTTCGGTGCTGCATGGTTCAAA
GTGGCGGCGGGATTGGCGCTGCCCATCCTTGCGTCGTGGGAGACCAATCGCGGTGACTGGCCCT
CGGAGTTCACCGGCTGGCGCGTCGGCGTCTTCGGGTTACCTTCGGGACAGCAACTCGTGGTGAG
CAGTCAGCCTGGCACCGGAGCCCCTCCGTCCCAGGCGAAGGTCGACGCGCTGCGGGTGGCGCTG
CAGGGACTACTGGGCTGA

>Brig1
MSARERVADLWDEVVANWQPGQDVWPGPLDDWFKSYRGRGSGAVDLDQYPDPWVGDLRGRVREP
RLVVLGLNPGIGYEELQGPNGTWTKRILDTGYSHCLHRSPPEDPAGWIPVHKRPSAYWVRVEHF
ARRWLGDTSAGHRDILNFELYPWHSPKLTGGLASPPAIVREFVLDPVAEVGVPQVFAFGAAWFK
VAAGLALPILASWETNRGDWPSEFTGWRVGVFGLPSGQQLVVSSQPGTGAPPSQAKVDALRVAL
QGLLG*
