# Supplementary material for: DNA glycosylases provide antiviral defence in prokaryotes
Source: Nature. 2024 Apr 17;629(8011):410–6. doi: 10.1038/s41586-024-07329-9 (PMC11078745; doi:10.1038/s41586-024-07329-9)
Supplement: Supplementary file 2 — Reporting Summary [file 41586_2024_7329_MOESM2_ESM.pdf]

nature portfolio

Corresponding author(s): Luciano A. Marraffini

Last updated by author(s): 04/04/2024

Reporting Summary

Nature Portfolio wishes to improve the reproducibility of the work that we publish. This form provides structure for consistency and transparency in reporting. For further information on Nature Portfolio policies, see our Editorial Policies and the Editorial Policy Checklist.

Statistics

For all statistical analyses, confirm that the following items are present in the figure legend, table legend, main text, or Methods section.

n/a	Confirmed	
☐	☒	The exact sample size (n) for each experimental group/condition, given as a discrete number and unit of measurement
☐	☒	A statement on whether measurements were taken from distinct samples or whether the same sample was measured repeatedly
☐	☒	The statistical test(s) used AND whether they are one- or two-sided<br>*Only common tests should be described solely by name; describe more complex techniques in the Methods section.*
☒	☐	A description of all covariates tested
☒	☐	A description of any assumptions or corrections, such as tests of normality and adjustment for multiple comparisons
☐	☒	A full description of the statistical parameters including central tendency (e.g. means) or other basic estimates (e.g. regression coefficient) AND variation (e.g. standard deviation) or associated estimates of uncertainty (e.g. confidence intervals)
☐	☒	For null hypothesis testing, the test statistic (e.g. F, t, r) with confidence intervals, effect sizes, degrees of freedom and P value noted<br>*Give P values as exact values whenever suitable.*
☒	☐	For Bayesian analysis, information on the choice of priors and Markov chain Monte Carlo settings
☒	☐	For hierarchical and complex designs, identification of the appropriate level for tests and full reporting of outcomes
☒	☐	Estimates of effect sizes (e.g. Cohen's d, Pearson's r), indicating how they were calculated

Our web collection on statistics for biologists contains articles on many of the points above.

Software and code

Policy information about availability of computer code

Data collection	Thermo Fisher Design and Analysis Software, version 2.7<br>MiSeq Software v4.0
Data analysis	DefenseFinder (version as of Nov 2023) (https://defensefinder.mdmlab.fr/)<br>Prokaryotic Antiviral Defence LOCator (PADLOC), v1.0 (https://padloc.otago.ac.nz/padloc/).<br>GraphPad Prism version 10.1.0<br>PyMol version 2.5.5<br>SnapGene version 7.0<br>Adobe Photoshop 2022<br>FIJI (ImageJ), version 2.14.0/1.54f<br>Microsoft Excel, version 16.82<br>Geneious Prime version 2023.1.2<br>PyCharm 2020.1 (Community Edition)<br>Sickle (https://github.com/najoshi/sickle)<br>ABySS (https://github.com/bcgsc/abyss)<br>Medusa (http://combo.dbe.unifi.it/medusa)<br>NCBI blastn, blastp and PSI-BLAST (https://blast.ncbi.nlm.nih.gov/Blast.cgi); versions as of Nov 2023<br>HHpred, version 57c8707149031cc9f8edceba362c71a3762bdbf8 (https://toolkit.tuebingen.mpg.de/tools/hhpred)<br>AlphaFold2 (colabfold) (https://colab.research.google.com/github/sokrypton/ColabFold/blob/main/AlphaFold2.ipynb#scrollTo=kOblAo-xetgx)<br>Dali server (http://ekhidna2.biocenter.helsinki.fi/dali/)

Thermo Fisher Xcalibur Data Acquisition and Interpretation Software, version 4.3
UniDec version 6.0.4 (https://github.com/michaelmarty/UniDec/releases)
IQ-TREE 1.6.12 (http://www.iqtree.org/release/v1.6.12)
iTOL: Interactive Tree Of Life (https://itol.embl.de/)
PerkinElmer ChemDraw Version 22.0.0.22
Custom Python scripts used for data analysis are deposited at github.com/Marraffini-Lab/Hossain_etal_2024.

For manuscripts utilizing custom algorithms or software that are central to the research but not yet described in published literature, software must be made available to editors and reviewers. We strongly encourage code deposition in a community repository (e.g. GitHub). See the Nature Portfolio guidelines for submitting code & software for further information.

Data

Policy information about availability of data

All manuscripts must include a data availability statement. This statement should provide the following information, where applicable:

- Accession codes, unique identifiers, or web links for publicly available datasets
- A description of any restrictions on data availability
- For clinical datasets or third party data, please ensure that the statement adheres to our policy

Raw uncropped images of plaque assays and gels are provided in Supplementary Figure 1. Replicates of plaque assays are shown in Supplementary Figure 2. Where relevant, source data are provided for figures with graphs. Lists of strains, plasmids, bacteriophages, oligonucleotides and CRISPR spacers used in this study are available in Supplementary Data File 2. The raw FASTQ files for the next-generation sequencing experiments can be found at the NCBI Sequence Read Archive (SRA) under BioProject PRJNA1045662. Custom Python scripts used for data analysis are deposited at github.com/Marraffini-Lab/Hossain_etal_2024. The DNA sequence of the approximately 34.5 kb metagenomic DNA fragment harboring the brig1 gene is deposited in NCBI GenBank under accession code OR880862. The NCBI protein accession codes of the brig1 homologs from Nocardioides zhouii and Nocardioides anomalus reported in this study are WP_129427366.1 and WP_165228961.1, respectively. The reference T4 phage genome used for assembly of wild-type and mutant T4 phage genome sequences in this study is from NCBI (GenBank: AF158101.6). The crystal structure in Extended Data Fig. 3b is from Protein Data Bank (PDB) 4ZBY.

Research involving human participants, their data, or biological material

Policy information about studies with human participants or human data. See also policy information about sex, gender (identity/presentation), and sexual orientation and race, ethnicity and racism.

Reporting on sex and gender	N/A
Reporting on race, ethnicity, or other socially relevant groupings	N/A
Population characteristics	N/A
Recruitment	N/A
Ethics oversight	N/A

Note that full information on the approval of the study protocol must also be provided in the manuscript.

Field-specific reporting

Please select the one below that is the best fit for your research. If you are not sure, read the appropriate sections before making your selection.

☒ Life sciences ☐ Behavioural & social sciences ☐ Ecological, evolutionary & environmental sciences

For a reference copy of the document with all sections, see nature.com/documents/nr-reporting-summary-flat.pdf

Life sciences study design

All studies must disclose on these points even when the disclosure is negative.

Sample size	Sample sizes were not statistically predetermined, and chosen based on the number needed to reliably determine differences between groups. As is routine in microbiology, given large effect sizes such as the ones reported in this study, we chose to replicate most experiments in triplicate (with exceptions noted and exact sample sizes stated in figure legends).
Data exclusions	No data was excluded from the analysis.
Replication	All phage assays, excluding next-generation sequencing, were performed in 2-3 independent biological replicates, as indicated in the figure legends. The replicates of phage plaque assays are shown in Supplementary Figure 2. The number of replicates for DNA gels are indicated in the figure legends.
Randomization	Not relevant to this study as there are no animal nor human experiments, and the experimental outcome does not depend on the order in which samples were analyzed in the experiments. Additionally, due to the large effect sizes observed and the manuscript relying largely on

images of plaque assays with several log-fold effect sizes, randomization was not relevant due to the repeated large effect sizes, clear and distinct phenotypes, and the direct reporting of images over data quantifications for the most part in this study.

Blinding

Not relevant to this study as there are no animal nor human experiments, and the experimental outcome does not depend on the order in which samples were analyzed in the experiments. Additionally, due to the large effect sizes observed and the manuscript relying largely on images of plaque assays with several log-fold effect sizes, randomization was not relevant due to the repeated large effect sizes, clear and distinct phenotypes, and the direct reporting of images over data quantifications for the most part in this study.

Reporting for specific materials, systems and methods

We require information from authors about some types of materials, experimental systems and methods used in many studies. Here, indicate whether each material, system or method listed is relevant to your study. If you are not sure if a list item applies to your research, read the appropriate section before selecting a response.

Materials & experimental systems

n/a	Involved in the study	
☒	☐	Antibodies
☒	☐	Eukaryotic cell lines
☒	☐	Palaeontology and archaeology
☒	☐	Animals and other organisms
☒	☐	Clinical data
☒	☐	Dual use research of concern
☒	☐	Plants

Methods

n/a	Involved in the study	
☒	☐	ChIP-seq
☒	☐	Flow cytometry
☒	☐	MRI-based neuroimaging
